# Childhood mortality during and after acute illness in Africa and south Asia: a prospective cohort study

**DOI:** 10.1016/S2214-109X(22)00118-8

**Published:** 2022-04-12

**Authors:** The Childhood Acute Illness and Nutrition (CHAIN) Network, Abdoulaye Hama Diallo, Abu Sadat Mohammad Sayeem Bin Shahid, Ali Fazal Khan, Ali Faisal Saleem, Benson O Singa, Blaise Siezanga Gnoumou, Caroline Tigoi, Catherine Achieng Otieno, Celine Bourdon, Chris Odhiambo Oduol, Christina L Lancioni, Christine Manyasi, Christine J McGrath, Christopher Maronga, Christopher Lwanga, Daniella Brals, Dilruba Ahmed, Dinesh Mondal, Donna M Denno, Dorothy I Mangale, Emmanuel Chimezi, Emmie Mbale, Ezekiel Mupere, Gazi Md. Salauddin Mamun, Issaka Ouedraogo, James A Berkley, Jenala Njirammadzi, John Mukisa, Johnstone Thitiri, Joseph D Carreon, Judd L Walson, Julie Jemutai, Kirkby D Tickell, Lubaba Shahrin, MacPherson Mallewa, Md. Iqbal Hossain, Mohammod Jobayer Chisti, Molly Timbwa, Moses Mburu, Moses M Ngari, Narshion Ngao, Peace Aber, Philliness Prisca Harawa, Priya Sukhtankar, Robert H J Bandsma, Roseline Maimouna Bamouni, Sassy Molyneux, Shalton Mwaringa, Shamsun Nahar Shaima, Syed Asad Ali, Syeda Momena Afsana, Syera Banu, Tahmeed Ahmed, Wieger P Voskuijl, Zaubina Kazi

## Abstract

**Background:**

Mortality among children with acute illness in low-income and middle-income settings remains unacceptably high and the importance of post-discharge mortality is increasingly recognised. We aimed to explore the epidemiology of deaths among young children with acute illness across sub-Saharan Africa and south Asia to inform the development of interventions and improved guidelines.

**Methods:**

In this prospective cohort study, we enrolled children aged 2–23 months with acute illness, stratified by nutritional status defined by anthropometry (ie, no wasting, moderate wasting, or severe wasting or kwashiorkor), who were admitted to one of nine hospitals in six countries across sub-Saharan Africa and south Asia between Nov 20, 2016, and Jan 31, 2019. We assisted sites to comply with national guidelines. Co-primary outcomes were mortality within 30 days of hospital admission and post-discharge mortality within 180 days of hospital discharge. A priori exposure domains, including demographic, clinical, and anthropometric characteristics at hospital admission and discharge, as well as child, caregiver, and household-level characteristics, were examined in regression and survival structural equation models.

**Findings:**

Of 3101 children (median age 11 months [IQR 7–16]), 1120 (36·1%) had no wasting, 763 (24·6%) had moderate wasting, and 1218 (39·3%) had severe wasting or kwashiorkor. Of 350 (11·3%) deaths overall, 234 (66·9%) occurred within 30 days of hospital admission and 168 (48·0%) within 180 days of hospital discharge. 90 (53·6%) post-discharge deaths occurred at home. The proportion of children who died following discharge was relatively preserved across nutritional strata. Numerically large high-risk and low-risk groups could be disaggregated for early mortality and post-discharge mortality. Structural equation models identified direct pathways to mortality and multiple socioeconomic, clinical, and nutritional domains acting indirectly through anthropometric status.

**Interpretation:**

Among diverse sites in Africa and south Asia, almost half of mortality occurs following hospital discharge. Despite being highly predictable, these deaths are not addressed in current guidelines. A fundamental shift to a child-centred, risk-based approach to inpatient and post-discharge management is needed to further reduce childhood mortality, and clinical trials of these approaches with outcomes of mortality, readmission, and cost are warranted.

**Funding:**

The Bill & Melinda Gates Foundation.

## Introduction

Despite remarkable reductions in child mortality globally, more than 5 million children died in 2019, predominantly in low-income and middle-income countries (LMICs).^1^ Most seriously ill children access the health-care system at some point;^2^ however, the mortality risk in LMICs among children admitted to hospital and following discharge remains unacceptably high.^3^ Reported paediatric inpatient fatality varies from around 2% to more than 20%.^4^, ^5^ Although several risk scores based on clinical signs and anthropometry have been devised, none are in widespread use or have reduced case fatality at scale.^6^

Hospital admission in LMICs commonly occurs following a series of contacts with medical and traditional practitioners, and is influenced by beliefs, costs, and access.^7^, ^8^ Thus, variation in case fatality reflects how populations seek care at different health system levels, the staffing and resources available, and the local prevalence of malnutrition, HIV, and other comorbidities.

Between 2004 and 2008, children (aged <15 years) in Kenya were observed to have a more than seven times higher risk of mortality in the year following discharge from hospital than did their community counterparts.^9^ A subsequent systematic review of paediatric mortality following hospital discharge identified 24 studies, with post-discharge fatality ranging from 1–2% to more than 20%,^3^ reflecting diverse study designs, populations, and loss to follow-up.

In hospitals, syndrome-based protocols defined in national and WHO^10^ guidelines are widely adopted, although often not fully implemented. These guidelines provide some recommendations by severity for several common clinical syndromes (eg, pneumonia and dehydration). However, children with poor health commonly meet the criteria for multiple syndromes^11^ and can have concurrent undernutrition, chronic diseases, or challenging social circumstances.^12^, ^13^ Nevertheless, guidance for care and resource allocation does not consider these contexts^11^ or advise on post-discharge care and integration with community-based health services, apart from specialist services for some specific conditions (including malnutrition or HIV).

**Research in context**

**Evidence before this study**

Between Aug 1, 2016, and Sept 1, 2021, we searched PubMed without date or language restrictions for studies published from date of database inception to Sept 1, 2021, using the following search terms: ((“infant”[MeSH Terms] OR “infant”[All Fields] OR “infants”[All Fields] OR “infant s”[All Fields] OR (“child”[MeSH Terms] OR “child”[All Fields] OR “children”[All Fields] OR “child s”[All Fields] OR “children s”[All Fields] OR “childrens”[All Fields] OR “childs”[All Fields])) AND (“hospital mortality”[MeSH Terms] OR (“hospital”[All Fields] AND “mortality”[All Fields]) OR “hospital mortality”[All Fields] OR (“mortality”[All Fields] AND “hospital”[All Fields]) OR “mortality hospital”[All Fields])) AND (“acute”[All Fields] OR “acutely”[All Fields] OR “acutes”[All Fields] OR (“inpatient s”[All Fields] OR “inpatients”[MeSH Terms] OR “inpatients”[All Fields] OR “inpatient”[All Fields]) OR “post-discharge”[All Fields])) AND (“africa”[MeSH Terms] OR “africa”[All Fields] OR “africa s”[All Fields] OR “africas”[All Fields] OR (“asia”[MeSH Terms] OR “asia”[All Fields])). Epidemiological studies of children presenting to hospitals in low-income and middle-income countries (LMICs) report inpatient case fatality ranging from 2% to more than 20%, and a case fatality of 5–8% among all children discharged from hospital. However, previous studies have addressed inpatient mortality without addressing specific criteria for discharge or acknowledging post-discharge mortality. Furthermore, studies reporting post-discharge mortality are highly heterogeneous, with wide variation in inclusion criteria and duration of follow-up. Some studies of selected groups have suggested that the number of post-discharge deaths could equal or exceed that of deaths observed in hospital. Several paediatric prognostic scores exist in LMICs, predominantly from single centres or specific patient groups, yet none have achieved widespread use. Hospital admission of children with acute illness complicated by severe wasting has been consistently reported to be the predominant factor associated with mortality, with reported inpatient case fatality ranging from 8% to 16%, despite implementation of current WHO and national guidelines. Prognostic models have not generally included household or psychosocial factors, or characteristics of children at the time of discharge.

**Added value of this study**

The Childhood Acute Illness and Nutrition (CHAIN) prospective cohort study provides comprehensive data from a cohort of 3101 children admitted to nine hospitals in six countries across sub-Saharan Africa and south Asia, using harmonised procedures. Despite support to implement WHO and national guidelines, an average of 11% of children died between hospital admission and 6 months later (weighted for enrolment strata). Overall, 168 (48·0%) of a total 350 deaths occurred following hospital discharge, with the highest rates in the first month after discharge and most occurring at home. Besides nutritional status and illness severity, structural equation models showed the direct effects of HIV infection, other underlying medical conditions, adverse caregiver characteristics, and access to health care on both 30-day mortality and post-discharge mortality within 180 days. Indirect effects of child-level nutritional risk exposures, underlying medical conditions, and access to health care on mortality operated through nutritional status rather than illness severity. Post-discharge mortality within 180 days was as predictable as 30-day mortality (area under receiver operating characteristic curve 0·81 [95% CI 0·78–0·84]).

**Implications of all the available evidence**

The results from this study, together with previous findings, suggest that a fundamental change is needed for international paediatric guidelines, away from a continued incremental adaptation of syndromic guidelines and towards a structured risk-based and child-centred approach. Risks of both acute mortality (within 30 days) and post-discharge mortality include the key roles of caregiver adversities and comorbidities, and household adversities. Although anthropometry captures the effects of these risks to some extent, it does not adequately target specific caregiver and household psychosocial interventions. A risk-based approach to acute childhood illness could allow for more rational allocation of treatment and resources, specific assignment of responsibility for post-discharge care, and facilitation of access to emergency care for recently discharged children at highest risk and vulnerable families to help reduce residual child mortality.

To inform the development of interventions, we aimed to explore the epidemiology of deaths among young children with acute illness across sub-Saharan Africa and south Asia and to identify the modifiable biomedical and social factors that influence mortality within 30 days of hospital admission and during the first 180 days following hospital discharge.

## Methods

### Study design and participants

In this prospective, stratified cohort study (the Childhood Acute Illness and Nutrition [CHAIN] cohort),^14^ we systematically enrolled children aged 2–23 months with acute illness who had been admitted to one of nine hospitals in six countries across sub-Saharan Africa and south Asia (Bangladesh, Burkina Faso, Kenya, Malawi, Pakistan, and Uganda) between Nov 20, 2016, and Jan 31, 2019 (appendix pp 4–7). The following nine participating hospitals were selected to reflect various rural and urban environments, and differing prevalences of malaria and HIV: Dhaka Hospital and Matlab Hospital (Dhaka and Matlab, Bangladesh); Banfora Referral Hospital (Banfora, Burkina Faso); Kilifi County Hospital, Mbagathi County Hospital, and Migori County Referral Hospital (Kilifi, Nairobi, and Migori, Kenya); Queen Elizabeth Central Hospital (Blantyre, Malawi); Civil Hospital (Karachi, Pakistan); and Mulago National Referral Hospital (Kampala, Uganda).^15^

Nutritional status defined by anthropometry has been consistently shown to be a strong predictor of mortality.^16^, ^17^, ^18^ To increase the inclusion of children at high risk of mortality, enrolment was stratified by anthropometry using mid-upper-arm circumference (MUAC), which is more easily measured in children with poor health and is less affected by dehydration than is anthropometry based on weight-to-length ratios. Children meeting one of the following three strata were enrolled in a 2:1:2 ratio: no wasting (MUAC ≥12·5 cm [age ≥6 months] or MUAC ≥12·0 cm [age <6 months]), moderate wasting (MUAC 11·5–12·5 cm [age ≥6 months] or MUAC 11·0–12·0 cm [age <6 months]), and severe wasting (MUAC <11·5 cm [age ≥6 months] or MUAC <11·0 cm [age <6 months], or bilateral pedal oedema [kwashiorkor] unexplained by other medical causes), thus deliberately over-representing children with moderate wasting and severe wasting or kwashiorkor. Children were screened for eligibility at hospital admission for non-surgical and non-traumatic conditions. The full list of exclusion criteria are provided in the appendix (p 3).

Ethical approval was obtained from the Oxford University Tropical Research Ethics Committee and the ethics committees at all participating institutions (appendix p 4). Written informed consent was obtained from parents or caregivers of all participants.

The protocol for this study is available online.^14^

### Procedures

Standardised data on demographics, medical history, underlying medical conditions, and clinical examination were collected on paper forms by trained study clinicians and entered into a REDCap database (appendix p 8). A complete examination of blood cell count, plasma glucose concentration, rapid malaria test, and HIV testing were conducted systematically. Social, household-level, and primary caregiver information was collected within 48 h, including maternal mental health (Patient Health Questionnaire-9); education, income, employment, assets, water, and sanitation (Demographic and Health Survey questionnaires); food security (adapted Household Food Insecurity Access Scale); and means, time, and cost of travel to hospital.^19^, ^20^, ^21^, ^22^ Household coordinates were collected by GPS after discharge or death of inpatient, and distance to the study hospital and the nearest health facility was calculated. Comprehensive definitions of all study variables are given in the appendix (pp 6–7).

A pre-study audit was conducted to identify areas of divergence from national and WHO guidelines,^5^ and ongoing care audits were undertaken using the study database. CHAIN study staff were embedded within hospitals to support inpatient care, training, and adherence to national guidelines. Comprehensive internal quality control and external study monitoring were conducted with Westat (Rockville, MD, USA).

At discharge, full clinical examination and anthropometry were performed. Children with severe wasting or kwashiorkor were discharged from hospital when feeds were completed and were all linked to an outpatient therapeutic feeding programme. Although these children received therapeutic feeds as part of outpatient nutritional rehabilitation, children with moderate wasting did not routinely receive supplementary feeds following hospital discharge, reflecting current policy at the study sites. A single home visit was undertaken after discharge or death of the patient and study follow-up visits were scheduled at days 45, 90, and 180 after hospital discharge at the recruiting hospital, whereby vital status, anthropometry, and health were assessed. Non-attendees were traced through home visits or by telephone.

Further information on data collection tools and standard operating procedures can be found on the CHAIN Network website.

### Outcomes

In line with best practices on reporting mortality^23^ and unaffected by differential discharge decisions, co-primary outcomes were 30-day mortality (ie, deaths within 30 days of hospital admission) and post-discharge mortality (ie, deaths within 180 days of hospital discharge).

### Statistical analysis

Pearson's χ^2^ test suggested that 2600 participants were required after hospital discharge for 80% power to detect a difference in case fatality of 2·4% in children with no wasting versus 5·1% in children with moderate wasting (assuming α=0·05), allowing 10% loss to follow-up and a two-sided hypothesis. These values were based on previous data from Kenya.^19^

All analyses were done according to an a priori statistical analysis plan, which accounted for the stratified design, specified the handling of missing data and imputation methods, and followed the Strengthening the Reporting of Observational Studies in Epidemiology guidelines (appendix pp 56–70).

We first examined the simple proportions of deaths: during the index hospital admission, during the first 30 days of admission, and following discharge; and the proportions of post-discharge deaths, at home or at a health facility. Incidence was estimated for 30-day mortality and post-discharge mortality within 180 days.

Exposures were investigated using two analytical methods. Standard survival models and structural equation modelling (SEM) were used to describe pathways according to an a priori conceptual framework (appendix p 46) based on the well established UNICEF conceptual framework linking immediate and underlying exposures to undernutrition, illness, and mortality. We grouped exposures a priori into domains that might influence mortality according to this framework (appendix p 46). We validated the selection of variables in domains using confirmatory factor analysis (appendix p 12). Domain scores were estimated as standalone latent variables using the underlying domain variables and were categorised into tertiles (appendix p 33).

For compatibility with previous studies, we first conducted a simple survival analysis using mixed-effects Weibull regression models (site as a random effect), inverse probability weighted to reflect estimated prevalence of nutritional strata among all paediatric admissions and loss to follow-up by site, and nutritional strata (27 groups). Children lost to follow-up were censored at the time last known to be alive. We reported adjusted hazard ratios (HRs) with 95% CIs. Sensitivity analyses examined various sampling weights that were plausible in similar settings; classification of nutritional status by weight-for-age and weight-for-length rather than MUAC; the inter-relationship of effects of the two central domains, MUAC, and illness severity on mortality; and characteristics associated with earlier and later deaths that occurred following discharge (appendix pp 38–42).

To generate a more comprehensive understanding of the relationships and mediation between domains and their direct and indirect effects on mortality, we used SEM for 30-day survival and 180-day post-discharge survival according to our a priori framework using a Weibull distribution, a random intercept for site, and weighted as per the survival regression analyses. Outcomes were adjusted for age and sex. In reporting, we distinguish direct pathways (from a domain to an outcome) and indirect pathways (from a domain to an outcome via another domain). SEM findings were considered to be the primary outputs on effects of exposures.

Multivariable model performance was assessed by bootstrapped area under receiver operating characteristic curves (AUCs; 1000 replications). Statistical analyses were done using Stata (version 15.1) and R (version 3.6.2). This study is registered at ClinicalTrials.gov, NCT03208725.

### Role of the funding source

The funder of the study had no role in study design, data collection, data analysis, data interpretation, or writing of the report.

## Results

3101 children, with a median age of 11 months (IQR 7–16), were enrolled between Nov 20, 2016, and Jan 31, 2019, of whom 1120 (36·1%) had no wasting, 763 (24·6%) had moderate wasting, and 1218 (39·3%) had severe wasting or kwashiorkor (appendix p 47). 1721 (55·5%) children were admitted to hospital after meeting criteria for diarrhoea, 667 (21·5%) for severe pneumonia, 440 (14·2%) for malaria, and 443 (14·3%) for severe anaemia. 182 (5·9%) children had been exposed to HIV and 106 (3·4%) had HIV infection. Overall, 175 (5·6%) children had been admitted to a hospital within the previous week. Among the 1218 individuals with severe wasting or kwashiorkor, eight (0·7%) were admitted to hospital due to a failed appetite test only, rather than clinical signs of illness. Participant, caregiver, and household characteristics are shown by strata in table 1 and by strata and site in the appendix (pp 15–23).

**Table 1:** Baseline characteristics

		**No wasting (n=1120)**	**Moderate wasting (n=763)**	**Severe wasting or kwashiorkor (n=1218)**	**All participants (n=3101)**
**Participant demographics**					
Age, months		11·0 (7·2–16·0)	11·0 (7·0–15·0)	10·0 (6·4–16·0)	11·0 (6·8–16·0)
Sex					
	Female	432 (38·6%)	329 (43·2%)	583 (47·9%)	1344 (43·3%)
	Male	688 (61·4%)	434 (56·9%)	635 (52·1%)	1757 (56·7%)
**Clinical presentation at admission**					
Signs of shock*					
	None	652 (58·2%)	489 (64·1%)	729 (59·9%)	1870 (60·3%)
	Some (≥1)	468 (41·8%)	274 (35·9%)	489 (40·1%)	1231 (39·7%)
Systemic inflammatory response syndrome†		395 (35·3%)	284 (37·2%)	386 (31·7%)	1065 (34·3%)
Impaired consciousness (not alert, responds to voice or pain, or unresponsive)		46 (4·1%)	47 (6·2%)	53 (4·4%)	146 (4·7%)
Severe pneumonia‡		286 (25·5%)	179 (23·5%)	202 (16·6%)	667 (21·5%)
Diarrhoea		525 (46·9%)	484 (63·4%)	712 (58·5%)	1721 (55·5%)
Malaria (positive rapid diagnostic test)		202 (18·0%)	106 (13·9%)	132 (10·8%)	440 (14·2%)
Anaemia§					
	None	243 (21·7%)	131 (17·2%)	216 (17·7%)	590 (19·0%)
	Mild	279 (24·9%)	164 (21·5%)	241 (19·8%)	684 (22·1%)
	Moderate	463 (41·3%)	345 (45·2%)	576 (47·3%)	1384 (44·6%)
	Severe	135 (12·1%)	123 (16·1%)	185 (15·2%)	443 (14·3%)
Blood glucose concentration					
	Normal	1041 (92·9%)	715 (93·7%)	1101 (90·4%)	2857 (92·1%)
	Abnormal¶	79 (7·1%)	48 (6·3%)	117 (9·6%)	244 (7·9%)
Anthropometry at admission					
Nutritional oedema (kwashiorkor)		0	0	353 (29·0%)	353 (11·4%)
Mid-upper arm circumference, cm		13·6 (1·0)	11·9 (0·4)	10·6 (1·4)	12·0 (1·7)
Weight-for-length Z score‖		−0·6 (1·2)	−2·4 (1·0)	−3·1 (1·6)	−2·0 (1·7)
Weight-for-age Z score‖		−1·1 (1·1)	−2·7 (0·9)	−4·0 (1·4)	−2·6 (1·7)
Length-for-age Z score		−1·1 (1·3)	−1·9 (1·3)	−3·1 (1·7)	−2·1 (1·7)
HIV status					
	Negative	1010 (90·2%)	693 (90·8%)	1034 (85·0%)	2737 (88·3%)
	Infected	15 (1·3%)	17 (2·2%)	74 (6·1%)	106 (3·4%)
	Exposed	56 (5·0%)	39 (5·1%)	87 (7·1%)	182 (5·9%)
	Refused testing or untested	39 (3·5%)	14 (1·8%)	23 (1·9%)	76 (2·5%)
**Underlying conditions**					
Stunting					
	None (Z score −2 and above)	851 (76·0%)	413 (54·1%)	293 (24·1%)	1557 (50·2%)
	Moderate (Z score −2 to −3)	193 (17·2%)	211 (27·7%)	322 (26·4%)	726 (23·4%)
	Severe (Z score up to −3)	76 (6·8%)	139 (18·2%)	603 (49·5%)	818 (26·4%)
Small birth size**		132 (11·8%)	132 (17·3%)	245 (20·1%)	509 (16·4%)
Chronic conditions††		59 (5·3%)	51 (6·7%)	93 (7·6%)	203 (6·5%)
Previous hospitalisation					
	None	868 (77·5%)	580 (76·0%)	860 (70·6%)	2308 (74·4%)
	<1 week previously	50 (4·5%)	52 (6·8%)	73 (6·0%)	175 (5·6%)
	1 week to 1 month previously	56 (5·0%)	42 (5·5%)	109 (8·9%)	207 (6·7%)
	>1 month previously	146 (13·0%)	89 (11·7%)	176 (14·4%)	411 (13·3%)
**Child-level nutritional risk exposures**					
Recommended appropriate diet		645 (57·6%)	431 (56·5%)	390 (32·0%)	1466 (47·3%)
Reported recent weight loss		109 (9·7%)	297 (38·9%)	713 (58·5%)	1119 (36·1%)
Reported poor feeding		80 (7·1%)	91 (11·9%)	366 (30·0%)	537 (17·3%)
Reported current breastfeeding		927 (82·8%)	631 (82·7%)	674 (55·3%)	2232 (72·0%)
**Caregiver characteristics**					
Primary caregiver is biological mother		1091 (97·4%)	731 (95·8%)	1134 (93·1%)	2956 (95·3%)
Primary caregiver currently ill		152 (13·6%)	138 (18·1%)	158 (13·0%)	448 (14·4%)
Caregiver's attained education level					
	None	236 (21·1%)	220 (28·8%)	345 (28·3%)	801 (25·8%)
	Primary (8 years in total)	486 (43·4%)	305 (40·0%)	533 (43·8%)	1324 (42·7%)
	Secondary or tertiary (12 years in total)	398 (35·5%)	238 (31·2%)	340 (27·9%)	976 (31·5%)
Maternal mental health (Patient Health Questionnaire-9 score)					
	None to mild (0–9)	970 (86·6%)	614 (80·5%)	933 (76·6%)	2517 (81·2%)
	Moderate to severe (≥10)	150 (13·4%)	149 (19·5%)	285 (23·4%)	584 (18·8%)
Maternal employment status					
	Self-employed	245 (21·9%)	133 (17·4%)	258 (21·2%)	636 (20·5%)
	Employed	100 (8·9%)	80 (10·5%)	113 (9·3%)	293 (9·4%)
	No reported income	775 (69·2%)	550 (72·1%)	847 (69·5%)	2172 (70·0%)
**Household-level exposures**					
Population density, 1000 people per km^2^		4·4 (0·4–14)	4·0 (0·7–19)	5·1 (0·4–19)	4·6 (0·5–16)
Assets index					
	Quintile 1 (least assets)	256 (22·9%)	169 (22·1%)	208 (17·1%)	633 (20·4%)
	Quintile 2	221 (19·7%)	156 (20·4%)	252 (20·7%)	629 (20·3%)
	Quintile 3	213 (19·0%)	153 (20·1%)	268 (22·0%)	634 (20·4%)
	Quintile 4	215 (19·2%)	141 (18·5%)	240 (19·7%)	596 (19·2%)
	Quintile 5 (most assets)	215 (19·2%)	144 (18·9%)	250 (20·5%)	609 (19·6%)
Household food insecurity					
	Low	713 (63·7%)	473 (62·0%)	659 (54·1%)	1845 (59·5%)
	Medium	302 (27·0%)	186 (24·4%)	333 (27·3%)	821 (26·5%)
	High	105 (9·4%)	104 (13·6%)	226 (18·6%)	435 (14·0%)
Improved toilet		835 (74·6%)	606 (79·4%)	900 (74·0%)	2341 (75·5%)
Improved water source		946 (84·5%)	632 (82·8%)	986 (81·0%)	2564 (82·7%)
**Access to health care**					
Distance to study hospital, km		7·9 (3·9–18)	9·7 (4·5–21)	9·7 (5·2–22)	9·1 (4·5–21)
Distance to nearest health facility, km		1·3 (0·5–3·1)	1·2 (0·5–3·7)	1·3 (0·5–2·8)	1·3 (0·5–3·1)
Means of travel to study hospital					
	Bus, ambulance, car, or train	505 (45·1%)	315 (41·3%)	606 (49·8%)	1426 (46·0%)
	Walking, motorcycle, tuk tuk, or rickshaw	615 (54·9%)	448 (58·7%)	612 (50·2%)	1675 (54·0%)
Travel cost to study hospital, US$					
	<1	463 (41·3%)	269 (35·3%)	439 (36·0%)	1171 (37·8%)
	1–5	573 (51·2%)	402 (52·7%)	669 (54·9%)	1644 (53·0%)
	≥5	84 (7·5%)	92 (12·1%)	110 (9·0%)	286 (9·2%)
Travel time to study hospital, h					
	<1	526 (47·0%)	316 (41·4%)	447 (36·7%)	1289 (41·6%)
	1–2	414 (37·0%)	272 (35·6%)	457 (37·5%)	1143 (36·9%)
	≥2	180 (16·1%)	175 (22·9%)	314 (25·8%)	669 (21·6%)

Data are median (IQR), n (%), or mean (SD).

* Capillary refill time >3 s, upper limb temperature gradient, weak pulse.

† Presence of two of the following four criteria: heart rate low (<90 bpm) or high (>180 bpm), temperature low (<36·0°C) or high (≥38·5°C), respiratory rate high (>34 breaths per min), and white blood cell count low (<5·0 cells per μL) or high (>17·5 cells per μL).

‡ Cough or difficulty in breathing with oxygen saturation <90%, central cyanosis, or grunting; very severe chest indrawing or inability to breastfeed or drink; or lethargy, reduced level of consciousness, or convulsions.

§ Anaemia by haemoglobin defined as none (>110 g/L), mild (100–110 g/L), moderate (70–100 g/L), and severe (<70 g/L).

¶ Defined as blood glucose concentration <3 mmol/L or >10 mmol/L.

‖ Children with oedema excluded from these values.

** Defined as reported low birthweight (<2·5 kg) or premature birth.

†† Chronic conditions include thalassaemia, cerebral palsy, sickle cell disease, congenital cardiac disease, and known tuberculosis.

116 (3·7%) children were lost to follow-up; 63 (5·6%) of 1120 children with no wasting, 25 (3·3%) of 763 with moderate wasting, and 28 (2·3%) of 1218 with severe wasting or kwashiorkor (p<0·0001; appendix pp 24, 49). Loss to follow-up also varied by site (p<0·0001), with the highest proportion among children with no wasting in Malawi due to a period of community myths nationally, which included theories about use of blood drawn for research studies.

Overall, 350 children died between hospital admission and 180 days following hospital discharge. Causes of death and mortality by clinical syndromes at admission, by location, and by site are provided in the appendix (pp 27–32).

During index hospitalisation, 182 (5·9%) of all 3101 children died (52·0% of all 350 deaths): 22 (2·0%) of 1120 children with no wasting, 32 (4·2%) of 763 with moderate wasting, and 128 (10·5%) of 1218 with severe wasting or kwashiorkor (table 2; appendix p 47). Median time to death from hospital admission was 1·5 days (IQR 0·0–4·0) among children with no wasting, 2·0 days (1·0–5·5) among those with moderate wasting, and 3·0 days (1·0–8·0) among those with severe wasting or kwashiorkor (p=0·15).

**Table 2:** Mortality by time period and cohort strata

	**No wasting (n=1120)**	**Moderate wasting (n=763)**	**Severe wasting or kwashiorkor (n=1218)**
**All deaths**			
Number of deaths (%)	39 (3·5%)	62 (8·1%)	249 (20·4%)
Risk ratio (95% CI)*	1 (ref)	2·54 (1·77–3·66)	5·89 (3·68–9·43)
**Deaths during index admission**			
Number of deaths (%)	22 (2·0%)	32 (4·2%)	128 (10·5%)
Risk ratio (95% CI)*	1 (ref)	2·37 (1·64–3·41)	5·43 (3·55–8·31)
**30-day mortality**†			
Number of deaths (%)	26 (2·3%)	40 (5·2%)	168 (13·8%)
Mortality rate, deaths per 1000 child-months (95% CI)	24·1 (16·5–36·4)	55·0 (40·5–76·5)	156 (134–183)
Rate ratio (95% CI)‡	1 (ref)	2·37 (1·42–3·95)	6·38 (4·16–9·78)
**Post-discharge mortality**§			
Number of deaths (%)	17/1072 (1·6%)	30/724 (4·1%)	121/1078 (11·2%)
Mortality rate, deaths per 1000 child-months (95% CI)	2·72 (1·72–4·57)	7·32 (5·16–10·7)	21·0 (17·6–25·3)
Rate ratio (95% CI)‡	1 (ref)	2·73 (1·52–4·89)	7·05 (4·32–11·5)

* Risk ratios from multi-level mixed effects log-binomial regression models adjusted for sex, age, and site and inverse probability weighted for selection and loss to follow-up.

† Includes inpatient and post-discharge deaths within 30 days of hospital admission (fixed period).

‡ Rate ratios estimated using the Mantel-Cox method adjusted for sex, age, and site and inverse probability weighted for selection and loss to follow-up.

§ Includes deaths within 180 days of index hospital discharge (fixed period). The number of children discharged alive were 1072 children with no wasting, 724 with moderate wasting, and 1078 with severe wasting or kwashiorkor.

**Figure 1:**
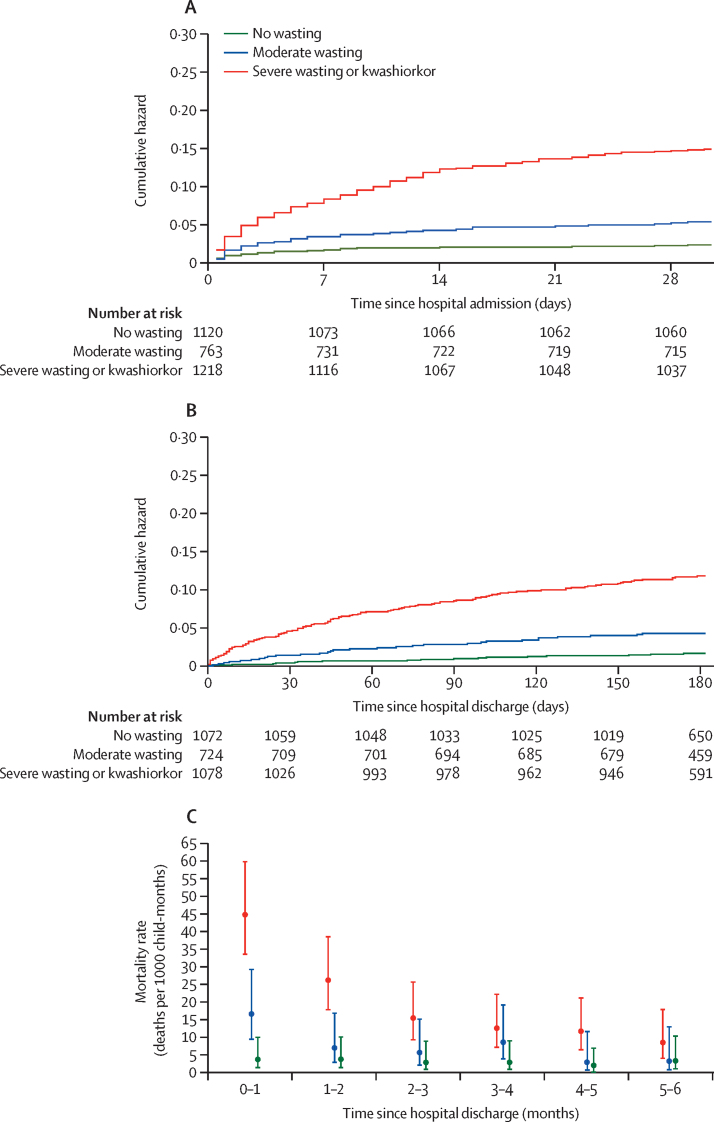
Cumulative hazard curves, stratified by nutritional status at hospital admission Data provided are adjusted for age, sex, and site. (A) Mortality in the first 30 days following hospital admission (2935 child-months). (B) Post-discharge mortality (15 999 child-months). (C) Monthly mortality rates following discharge from hospital.

234 deaths (66·9% of all 350 deaths) occurred within 30 days of hospital admission: 26 (2·3%) deaths among children with no wasting (24·1 deaths per 1000 child-months [95% CI 16·5–36·4]), 40 (5·2%) deaths among those with moderate wasting (55·0 [40·5–76·5]), and 168 (13·8%) among those with severe wasting or kwashiorkor (156·0 [134·0–183·0]; figure 1; table 2; appendix p 25).

Overall, 2874 children were discharged alive, did not withdraw from the study before discharge, and were followed up (figure 1; appendix p 47). Length of stay varied by site and strata: 3 days (IQR 2–5) among children with no wasting, 4 days (2–6) among those with moderate wasting, and 7 days (4–12) among those with severe wasting or kwashiorkor (p<0·0001; appendix p 50). 205 (7·1%) children left hospital against medical advice (appendix p 26). 22 (0·8%) discharges occurred after 30 days. Characteristics at discharge are shown in the appendix (p 26).

Overall, 168 (5·8%) of the 2874 children who were discharged from hospital alive died within 180 days of discharge (48·0% of all 350 deaths): 17 (1·6%) of 1072 children with no wasting (2·72 deaths per 1000 child-months [95% CI 1·72–4·57]), 30 (4·1%) of 724 with moderate wasting (7·32 [5·16–10·7]), and 121 (11·2%) of 1078 with severe wasting or kwashiorkor (21·0 [17·6–25·3]; figure 1; table 2; appendix pp 26, 47). Mortality was highest in the first month following hospital discharge, when 62 (36·9%) of 168 deaths occurred (figure 1). 90 (53·6%) post-discharge deaths occurred at home rather than in a health facility. Home deaths were more common among children with wasting than among those without (p=0·037; appendix p 33).

The distributions of children within exposure domain scores are shown in the appendix (p 33).

Lower nutritional strata at hospital admission (ie, moderate wasting and severe wasting or kwashiorkor), age per log month, higher signs of illness severity at admission, exposure to HIV, and HIV infection were associated with 30-day mortality (table 3). Other domains associated with 30-day mortality were underlying pre-existing medical conditions, adverse caregiver and household-level exposures, and access to health care (table 3).

**Table 3:** Domains of exposures associated with 30-day and post-discharge mortality

		**30-day mortality**		**Post-discharge mortality**	
		Adjusted HR (95% CI)*	p value	Adjusted HR (95% CI)*	p value
**Base variables**					
Nutritional stratum at hospital admission					
	No wasting	1 (ref)	..	1 (ref)	..
	Moderate wasting	2·03 (1·40–2·94)	<0·0001	2·58 (1·47–4·52)	0·0012
	Severe wasting or kwashiorkor	4·51 (2·81–7·23)	<0·0001	4·36 (2·27–8·37)	<0·0001
Age, log months		0·77 (0·65–0·91)	0·0031	0·72 (0·55–0·94)	0·017
Female		1·19 (0·87–1·65)	0·28	1·33 (0·86–2·06)	0·19
Leaving against medical advice		..	..	2·14 (1·33–3·43)	0·0021
Admission duration, log days		..	..	1·51 (0·95–2·39)	0·083
Change in anthropometry at hospital discharge					
	No change	..	..	1 (ref)	..
	Improved	..	..	0·73 (0·57–0·93)	0·010
	Worsened	..	..	0·81 (0·15–4·40)	0·80
**Exposure domains**					
Signs of illness severity at admission					
	Low	1 (ref)	..	1 (ref)	..
	Medium	1·29 (0·76–2·19)	0·34	1·36 (0·83–2·22)	0·22
	High	3·82 (2·50–5·86)	<0·0001	1·76 (0·96–3·24)	0·067
Signs of illness severity at discharge					
	Low	..	..	1 (ref)	..
	Medium	..	..	1·29 (0·91–1·84)	0·15
	High	..	..	4·00 (1·66–9·64)	0·0020
HIV status					
	Negative	1 (ref)	..	1 (ref)	..
	Untested	0·78 (0·37–1·65)	0·52	1·88 (0·78–4·55)	0·16
	Exposed	1·68 (1·29–2·18)	<0·0001	1·82 (1·19–2·80)	0·0064
	Infected	2·45 (1·24–4·81)	0·010	1·75 (0·91–3·37)	0·094
Underlying medical conditions					
	Low	1 (ref)	..	1 (ref)	..
	Medium	2·45 (1·22–4·91)	0·012	0·90 (0·61–1·33)	0·60
	High	2·08 (1·25–3·45)	0·0054	1·40 (0·99–1·97)	0·058
Child-level nutritional risk exposures					
	Low	1 (ref)	..	1 (ref)	..
	Medium	0·59 (0·36–0·97)	0·036	0·83 (0·41–1·67)	0·61
	High	1·15 (0·78–1·69)	0·47	1·20 (0·75–1·90)	0·45
Caregiver characteristics					
	Least adverse	1 (ref)	..	1 (ref)	..
	Moderately adverse	2·03 (1·36–3·04)	0·0010	1·37 (0·82–2·30)	0·22
	Most adverse	1·53 (1·01–2·32)	0·045	2·11 (1·18–3·77)	0·012
Household-level exposures					
	Least adverse	1 (ref)	..	1 (ref)	..
	Moderately adverse	2·02 (1·35–3·03)	0·0011	1·04 (0·78–1·38)	0·80
	Most adverse	2·08 (1·38–3·13)	<0·0001	1·01 (0·68–1·50)	0·95
Access to health care					
	Least adverse	1 (ref)	..	1 (ref)	..
	Moderately adverse	1·10 (0·74–1·64)	0·64	1·09 (0·68–1·77)	0·71
	Most adverse	1·77 (1·14–2·75)	0·011	1·09 (0·64–1·85)	0·74
Site variance (95% CI)		0·05 (0·003–0·93)	..	0·02 (0·001–0·75)	..
Bootstrapped AUC (95% CI)		0·80 (0·78–0·83)	..	0·81 (0·78–0·84)	..

HR=hazard ratio. AUC=area under receiver operating characteristic curve.

* Adjusted HR for all predictors in the multivariable model. All model results were weighted using sampling and lost to follow-up weights. HRs from a multi-level multivariable parametric (Weibull distribution) survival model with site as a random effect.

Lower nutritional stratum at hospital admission (ie, moderate wasting and severe wasting or kwashiorkor), most adverse caregiver characteristics, higher signs of illness severity at hospital discharge, discharge against medical advice, and HIV exposure, but not HIV infection, were significantly associated with post-discharge mortality within 180 days (table 3). Age per log month and improved anthropometry at discharge were associated with a lower post-discharge mortality rate within 180 days (table 3). Sex, signs of illness severity at admission, underlying medical conditions, child-level nutritional risk exposures, household-level exposures, access to health care, and duration of admission were not independently associated with post-discharge mortality (table 3).

Sensitivity analyses across various plausible sampling weights in similar settings (appendix pp 34–40) and using different anthropometric indices, rather than MUAC (appendix p 52), provided results similar to the primary models. Examining the effects of MUAC on a continuous scale and admission illness severity together, we observed that the relationship between MUAC and mortality is highly dependent on illness severity (appendix p 53). Unlike later post-discharge deaths, deaths during the first month following discharge were associated with age and leaving against medical advice (appendix pp 41–42).

At hospital admission, the lowest risk quintile predicted by the regression models had a 0·8% risk of 30-day mortality and the highest quintile had a 24·0% risk (appendix p 42). At discharge, the lowest two risk quintiles (40% of discharged children) had a 0·9–1·2% risk of post-discharge mortality, and the highest risk quintile (20% of discharged children) had an 18·0% risk (appendix pp 42–43).

SEM full estimation and model performance results are provided in the appendix (pp 44–45), as are the confirmatory factor analysis results (pp 12–13).

**Figure 2:**
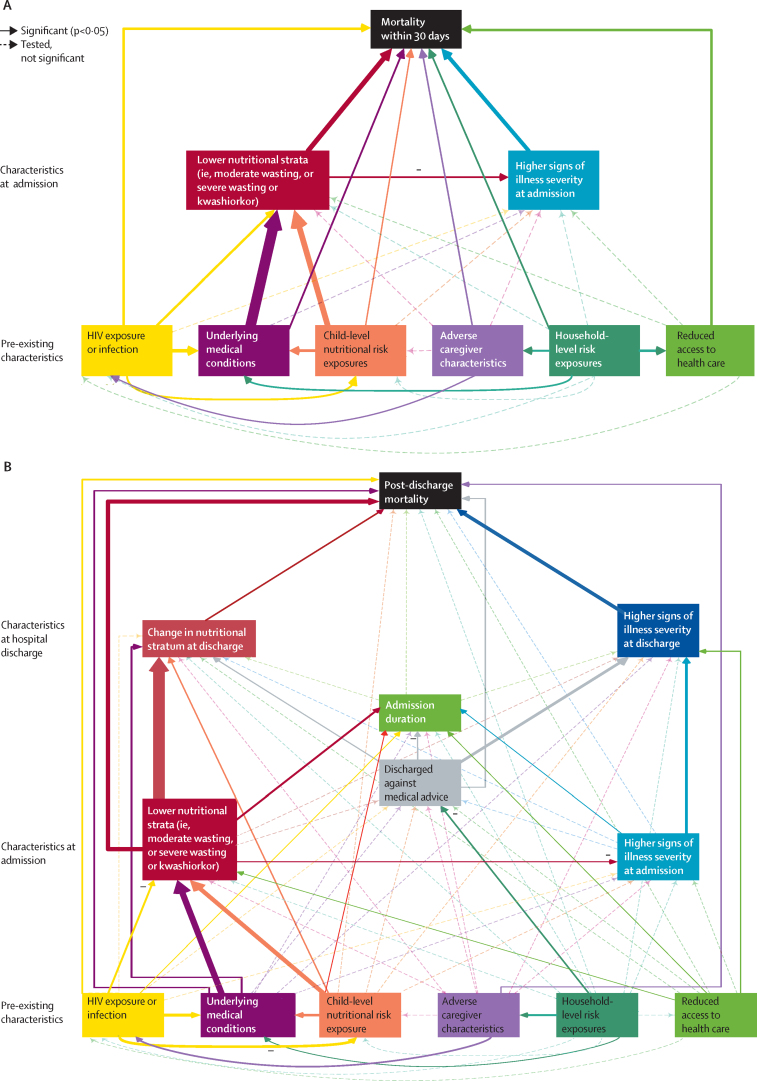
Structural equation models of relationships between domains of characteristics and 30-day mortality and post-discharge mortality (A) Relationships between domains of hospital admission and pre-existing characteristics and 30-day mortality. (B) Relationships between domains of hospital discharge, hospital admission, and pre-existing characteristics and post-discharge mortality. Significant associations (p<0·05) are indicated by solid arrows and non-significant associations by dashed arrows. Significant negative associations are indicated by the – symbol. For significant associations, the arrow thickness corresponds to the effect size. Each outcome was adjusted for age and sex and was modelled with a random intercept at the site level. The full estimation results are provided in the appendix (pp 44–45). Model results were weighted using sampling and lost to follow-up weights.

In the SEM, both lower nutritional strata (adjusted HR 2·07 [95% CI 1·42–3·04] for moderate wasting and 4·56 [2·63–7·90] for severe wasting or kwashiorkor *vs* no wasting) and high signs of illness severity at admission (3·67 [2·35–5·75]) at hospital admission were directly associated with 30-day mortality (figure 2A; appendix p 44). HIV infection (2·27 [1·25–4·12]), HIV exposure (1·51 [1·11–2·06]), underlying medical conditions, and child-level nutritional risk exposures directly influenced 30-day mortality, as well as acting through nutritional status (figure 2A; appendix p 44).

At hospital discharge, high signs of illness severity (adjusted HR 3·68 [95% CI 1·56–8·67]), improved anthropometry (0·70 [0·56–0·89]), and leaving against medical advice (2·05 [1·25–3·35]) were directly associated with post-discharge mortality within 180 days (appendix p 45). Many exposure domains directly affected duration of admission; however, duration of admission itself was not directly associated with post-discharge mortality, thus acting as a so-called sink (figure 2B; appendix p 45).

For the SEM models, bootstrapped AUCs (1000 replications) were 0·81 (95% CI 0·78–0·84) for 30-day mortality and 0·81 (0·77–0·84) for post-discharge mortality within 180 days. Removing all pre-existing characteristics from the models resulted in bootstrapped AUCs of 0·77 (0·74–0·79) for 30-day mortality and 0·78 (0·75–0·82) for post-discharge mortality.

Of the characteristics at hospital admission, nutritional stratum (adjusted HR 2·65 [95% CI 1·49–4·70] for moderate wasting and 4·45 [2·21–8·96] for severe wasting or kwashiorkor), but not signs of illness severity, directly affected post-discharge mortality within 180 days (figure 2B; appendix p 45). However, signs of illness severity at hospital admission influenced signs of illness severity at discharge, thereby being indirectly associated.

Of the six domains of pre-existing characteristics, HIV exposure (adjusted HR 1·63 [95% CI 1·09–2·44]), high underlying medical conditions (1·37 [1·04–1·82]), and most adverse caregiver characteristics (2·00 [1·16–3·46]) were directly and independently associated with post-discharge mortality within 180 days (figure 2B; appendix p 45).

Several important indirect effects were observed. Household-level exposures influenced leaving against medical advice, caregiver characteristics, and underlying medical conditions. Reduced access to health care, underlying medical conditions, and child-level nutritional risk exposures affected admission nutritional status, and child-level nutritional risk exposure was also associated with underlying medical conditions. Additionally, underlying medical conditions affected the change in nutritional status from hospital admission to discharge. Pre-existing characteristics were not associated with signs of illness severity at hospital admission or discharge. In the confirmatory factor analysis, caregiver employment, maternal mental health, and caregiver education were the main measures of the caregiver characteristics domain (appendix p 12). In both the 30-day mortality and post-discharge mortality models, lower nutritional strata were associated with less severe signs of illness at hospital admission.

## Discussion

Many children in low-resource settings are at increased risk of morbidity and mortality, despite increasing (although inequitable) access to interventions, including vaccination and primary health care.^24^ In the children admitted to hospital in this study, we observed initially steep mortality curves, reducing in gradient after around 3 weeks, but not flattening over 6 months. Across different geographical settings, almost half of all deaths occurred after discharge from hospital, commonly within a few weeks of discharge, and around half of post-discharge deaths occurred at home. The ratio of inpatient to post-discharge deaths was preserved across diverse geographical settings and anthropometric strata, matching the highest estimates previously reported.^3^

Low nutritional status defined by anthropometry (ie, malnutrition) was associated with increased mortality, even though these children received medical and nutritional care in line with WHO and national guidelines. In the context of severe acute illness, our data show that low MUAC represents a high-risk state associated with mechanisms beyond reduced nutrient intake in the short term. Importantly, children with low MUAC admitted with low illness severity had a far lower risk of mortality, as seen in programmes for uncomplicated severe malnutrition, than did those with higher illness severity. These findings suggest that acutely ill children with wasting would benefit from greater risk stratification to target interventions beyond therapeutic and supplemental feeding in the short term, including before hospitalisation. General risk stratification among paediatric admissions might permit children at low risk to be identified according to formal criteria and discharged from hospital earlier, redirecting staff and resources to care for children at increased risk and saving costs to families.

Post-discharge mortality was as predictable as 30-day mortality using information at hospital admission, hospital discharge, and social circumstances. Clinical features at hospital discharge have not been previously reported^17^, ^18^ and, unsurprisingly, these were more strongly associated with post-discharge death than were admission features. Importantly, although multiple pathways converged on length of hospital stay, this was not associated with post-discharge mortality, suggesting that extending inpatient treatment is unlikely to reduce mortality for children who are judged to be ready for discharge. As expected, leaving hospital against medical advice was associated with an increased risk of mortality, suggesting a need to identify these families early to address concerns and provide support to avoid premature discharge. Biomarkers might provide additional information on risk at hospital admission and at the time of clinically apparent readiness for discharge,^25^ but this needs further research.

Caregiver characteristics, a domain in which adversity was dominated by maternal employment and mental health, directly influenced post-discharge mortality and was a more important domain than were household-level exposures or access to health care. These findings support earlier work investigating the roles, support, and finances of caregivers^12^, ^26^ and suggest that interventions specifically targeting maternal wellbeing, agency, and independent income are needed. These findings might also partly explain the observed inefficacy of individual interventions against post-discharge mortality in recent clinical trials.^3^, ^27^, ^28^, ^29^

Given that just over half of post-discharge deaths occur at home,^3^, ^30^ so-called down referral to the specific responsibility of a named community health worker could be a valuable strategy, such as the role of health visitors in high-income settings, which is not included in current WHO or national guidelines. Training for mothers and family members in recognising danger signs in infants and continued structured contact with a health professional could be provided at low cost by telephone and SMS to deliver advice and to allow for early recognition of children who need face-to-face evaluation or readmission. Facilitating access to emergency rooms for recently admitted children, avoiding queueing, and waiving hospital costs (including bed charges for the caregiver), could help to overcome hesitancy in re-presenting to hospital.^12^

Most findings of the simple regression analysis were replicated in the SEM analysis. Importantly, indirect effects of child-level nutritional risk exposures, underlying conditions, and household characteristics operated via anthropometric status rather than illness severity. Surprisingly, neither model found an association between access to health care and post-discharge mortality. The association between access to health care and 30-day mortality probably reflects delays in initial presentation and its correlates. HIV exposure and infection were associated with 30-day mortality in both models; however, HIV infection was not associated with post-discharge mortality in either model, potentially because of low numbers.

Strengths of the CHAIN cohort study include its size, geographical diversity, standardised care, and comprehensive data collection across a range of geographical and epidemiological settings. Limitations include potential bias by children admitted overnight and dying before enrolment in the morning, and the fact that weekend admissions were not targeted. We faced challenges in differentiating situational stressors and experiences from long-term psychosocial strain, not using a biochemical method to determine nutrient status, achieving full guideline adherence, and obtaining some data for enrolled children who died early on in the study. Study follow-up visits might have resulted in health service contact that would not normally occur. Causes of death are based on all available information, but might be unreliable. We chose to retain sepsis as a cause of death because a primary focus of infection was not always evident or children met the criteria for multiple syndromes, or both. Although the current analysis focuses on understanding broad pathways involved in mortality, prognostic models for individual clinical features and models of biological systems will be published in separate reports, as will analyses of post-discharge growth and readmission to hospital.

Acute illness and wasting in children represents a high-risk state, in which various biological and social vulnerabilities converge to drive child mortality independent of clinical syndrome at presentation to hospital, and despite support to provide treatment in line with guidelines. Almost half of all deaths observed in this study occurred following hospital discharge and were as predictable as deaths within 30 days of hospital admission. A fundamental shift towards a child-centred, risk-based approach to guidelines with a structured assessment at hospital admission and at discharge to inform appropriate care and social support care is needed to further reduce childhood mortality in low-resource settings.

## Data sharing

The CHAIN cohort data and analysis code are deposited and may be requested at the Harvard Dataverse website.

## Declaration of interests

All members of the writing group declare no competing interests.
