# Supplementary material for: Childhood mortality during and after acute illness in Africa and south Asia: a prospective cohort study
Source: Lancet Glob Health. 2022 Apr 12;10(5):e673–84. doi: 10.1016/S2214-109X(22)00118-8 (PMC9023747; doi:10.1016/S2214-109X(22)00118-8)
Supplement: Supplementary appendix [file mmc1.pdf]

THE LANCET
Global Health

Supplementary appendix

This appendix formed part of the original submission and has been peer reviewed. We post it as supplied by the authors.

Supplement to: The Childhood Acute Illness and Nutrition (CHAIN) Network. Childhood mortality during and after acute illness in Africa and south Asia: a prospective cohort study. *Lancet Glob Health* 2022; **10:** e673–84.

Childhood mortality during and after acute illness in sub-Saharan Africa and South Asia - The CHAIN cohort study

The Childhood Acute Illness and Nutrition (CHAIN) Network

Supplementary Appendix

Table of Contents

Supplementary Methods **3**

Participating sites **3**

Inclusion & exclusion criteria **3**

Ethics approval **4**

Data collection **4**

Anthropometry 4

Household and caregiver data 4

Clinical definitions **6**

Laboratory procedures and definitions **7**

Causes of deaths **7**

Data management **8**

Data Processing 8

Missing data 8

Table S1. List of variables with missing data and methods of imputation. 9

Statistical methods **9**

Table S2. Inverse sample weights. 10

Table S3. Variables included in the multivariable regression models. 11

Table S4. Confirmatory Factor Analysis of Exposure Domains. 12

Table S5. Summary of Information criteria used to select parametric survival regression model. 13

Sensitivity Analyses **13**

Structural Equation Modelling **13**

Supplementary results ***14***

Table S6. Enrolment by site. 14

Table S7. Kilifi County Hospital, Kenya baseline characteristics. 15

Table S8. Mbagathi Hospital, Kenya baseline characteristics. 16

Table S9. Migori County Hospital, Kenya baseline characteristics. 17

Table S10. Mulago National Referral Hospital, Uganda baseline characteristics. 18

Table S11. Queen Elizabeth Central Hospital, Malawi baseline characteristics. 19

Table S12. Banfora Regional Referral Hospital, Burkina Faso baseline characteristics. 20

Table S13. Dhaka Hospital, Bangladesh baseline characteristics. 21

Table S14. Matlab Hospital, Bangladesh baseline characteristics. 22

Table S15. Karachi Civil Hospital, Pakistan baseline characteristics. 23

Table S16. Lost-to-follow-up and withdrawal from admission to 180 days after discharge. 24

Table S17. Lost-to-follow-up and withdrawals from admission to 180 days after discharge by site. 24

Table S18. Follow-up time and mortality rates at pre-specified time points by enrolment strata. 25

Table S19. Participants selected characteristics at index discharge. 26

Table A20-A. Attributed causes of death (all deaths). 27

Table S20-B. Attributed causes of death (death during index admission). 28

Table S20-C. Attributed causes of death (death within 30 days of date of index admission) ... 29
Table S20-D. Attributed causes of death (death after discharge from index admission) ... 30
Table S21. Deaths by admission clinical syndrome ... 31
Table S22. Location of post-discharge deaths. ... 31
Table S23. Inpatient, 30-day and post-discharge deaths stratified by site and anthropometric strata. ... 32
Table S24. Distribution of domain classifications by admission anthropometry. ... 33
Table S25. Base model controlling for both sampling and LTFU weights. ... 34
Table S26. Base model without sample weights, with LTFU weights only. ... 34
Table S27. Base model with nutritional status defined using weight-for-length z score. ... 34
Table S28. Base model with nutritional status defined using weight-for-age z score. ... 35
Table S29. Base model with nutritional status defined using length-for-age z score. ... 35
Table S30. Characteristics associated with 30 day and post discharge mortality without sampling weights, but with LTFU weights only. ... 36
Table S31. Characteristics associated with 30 day and post discharge mortality with low sampling weights (NW=82%, MW=8% and SWK=10%). ... 38
Table S32. Characteristics associated with 30 day and post discharge mortality with high sampling weights (NW=46%, MW=24% and SWK=30%). ... 39
Table S33. Characteristics associated with 30 day and post discharge mortality with anthropometric strata. ... 40
Table S34: Characteristics associated with early (within 30 days from discharge) and late (after 30 days from discharge) post-discharge mortality. ... 41
Table S35. 30-day mortality across quintiles of predicted mortality from the final 30-day regression model including sample weights and LTFU. ... 42
Table S36. Post-discharge mortality across quintiles of predicted mortality from the final post-discharge regression model including sample weights and LTFU. ... 42
Table S37. Discharge against medical advice across quintiles of predicted mortality from the final post-discharge regression model including sample weights and LTFU. ... 43
Table S38. Structural Equation Model full estimation results of the relationships between pre-existing characteristics, characteristics at admission, and mortality within 30 days ... 44
Table S39. Structural Equation Model results for the relationships between pre-existing characteristics, characteristics at admission and discharge, and post-discharge mortality. ... 45

Supplementary Figures ... 46

Figure S1. CHAIN causal framework adapted from UNICEF. ... 46
Figure S2. Study Flow ... 47
Figure S3. Cox-Snell residual plots. ... 48
Figure S4. Cumulative hazard curves for LTFU and withdrawals combined. ... 49
Figure S5. Length of inpatient stay. ... 50
Figure S6. Proportions of index admission and post-discharge deaths by anthropometric strata. ... 51
Figure S7. Cumulative hazard curves of mortality in the first 180 days after admission ... 52
Figure S8. Predicted mortality by admission illness severity across continuous MUAC values. ... 53
Figure S9. Admission Characteristics across quintiles of 30-day mortality regression predictions. ... 54
Figure S10. Discharge Characteristics across quintiles of post-discharge mortality regression predictions. ... 55

Statistical analysis plan ... 56

STROBE Statement ... 69

Supplementary References ... 71

Supplementary Methods

Participating sites

We enrolled infants from nine hospitals in six countries across Africa and South Asia (**Table S6**). Four sites were predominantly rural: Matlab hospital in Bangladesh, Kilifi County Hospital in Kenya, Migori sub-county hospital in Kenya and Banfora regional hospital in Burkina Faso. The under-5s mortality rate in all the sites in 2017 were: 38, 38, 81, 49, 49, 49, 55, 64 and 89 deaths/10000 live births at Dhaka Hospital (Bangladesh), Matlab Hospital (Bangladesh), Karachi Civil Hospital (Pakistan), Kilifi County Hospital (Kenya), Mbagathi sub-County Hospital (Kenya), Migori sub-County Hospital (Kenya), Mulago Hospital (Uganda), Queen Elizabeth Central Hospital Blantyre (Malawi) and Banfora Regional Hospital (Burkina Faso) respectively. Details of the site population and disease epidemiology and the hospital characteristics are described in a separate paper[1]

Sites were asked to recruit approximately five children per week, 2 severely wasted/kwashiorkor (SWK), 2 moderately wasted (MW) and 1 not wasted (NW) by recruiting the first eligible child admitted in each stratum beginning on a fixed day each week. Written informed consent was obtained in participants' local language.

Inclusion & exclusion criteria

Inclusion criteria

- Age 2 to 23 months.
- Admitted to hospital with a medical illness
- Meeting criteria for one of three strata:
  - **Not wasted (NW)**: MUAC ≥12·5cm (age ≥6mo) or MUAC ≥12cm (age <6mo).
  - **Moderately wasted (MW)**: MUAC 11·5 to <12·5cm (age ≥6mo) or MUAC 11 to <12cm (age <6mo).
  - **Severely wasted or kwashiorkor (SWK)**: MUAC <11·5cm (age ≥6mo) or MUAC<11cm (age <6mo) or bilateral pedal oedema unexplained by other medical causes.

Exclusion criteria

- Currently undergoing CPR or imminent cardiac arrest
- Hospitalization for trauma
- Hospitalization for a condition requiring surgery within 6 months
- Known terminal illness expected to result in death within 6 months
- Suspected chromosomal abnormality
- Unable to tolerate oral feeds prior to current illness
- Previous inclusion of this child or a sibling in this study
- Lack of willingness to participate in follow-up visits for 6 months
- Lack of caregiver informed consent

Ethics approval

The study was approved by the following ethical committees from all the recruiting and coordinating countries:

Data collection

Country	Ethics committees
UK	Oxford Tropical Research Ethics Committee
Kenya	Scientific & Ethical Review Unit (SERU), Kenya Medical Research Institute
USA	University of Washington Institutional Review Board<br>Oregon Health and Science University Institutional Review Board
Uganda	Makerere University School of Biomedical Sciences Research Ethics Committee
Pakistan	Ethical Review Board, Aga Khan University
Bangladesh	International Centre for Diarrheal Disease Research: Research Review Committee (RRC) and Ethical Review Committee (ERC)
Malawi	COMREC, Kamuzu University of Health Sciences, Malawi
Burkina Faso	Comité d'éthique institutionnel du Centre MURAZ
Canada	Research Ethics Board of the Hospital for Sick Children
The Netherlands	Medical Ethics Review Committee, Amsterdam UMC, The Netherlands

All CRFs and SOPs are available at https://chainnetwork.org/resources/.

Anthropometry

Anthropometry was performed by trained clinical assistants, including MUAC to the nearest mm using a non-stretch insertion tape (TALC, St. Albans, UK), length to the nearest mm (Seca 416 infantometer (Birmingham, UK)) and weight (Seca 825 electronic scale (Birmingham, UK) calibrated monthly) to the nearest 10g. Caregiver MUAC utilized adult insertion tapes, weight (Seca 825 scales) and height (Seca 215 stadiometer). All anthropometric measurement z-scores were calculated using the WHO 2006 growth reference.[2]

Household and caregiver data

Caregivers were interviewed during admission on their physical and mental health, social, household and access to healthcare. Physical health included their anthropometric measurements and whether the mother was also sick at the time of the interview. Caregiver's mental health was assessed using Patient Health Questionnaire 9-item (PHQ-9) tool with an additional tenth question on overall functional impairment.[3,4] The PHQ-9 tool has been validated and applied in LMICs context[5–8] including when translated to local languages such as in East Africa.[8] PHQ-9 total scores range from 0 to 27 because each 9 items have responses from 0 ("not at all") to 3 ("nearly every day"). We created categories based on cut-offs at 0, 4, 9, 14 and 20 to represent screening of depression symptom as none, minimal, mild, moderate, moderately severe and severe. We also created another variable with a single screening cut-off point of greater than 10 to represent major depression.[3]

A set of eight questions from Food Insecurity Experience Scale (FIES) was adapted and asked to caregivers to assess household food insecurity.[9,10] A total score for each participant was derived by adding the responses to (yes/no) the

questions. A categorical variable defining food insecurity was created with a score of 0-3, 4-6 and 7-8 defined as low, moderate and severe food insecurity respectively.[11]

Child dietary diversity was assessed by identifying the different food groups that the child ate on a typical day. Breast milk plus 7 expected food groups (grains, roots and tubers, legumes and nuts, dairy products, flesh foods, eggs, fruits and vegetables) responses were summed to obtain total scores. Fruits and vegetables in the study were separated and not grouped in Vitamin A rich fruits and vegetables vs. other fruits and vegetables therefore combined them to form one food group.

Recommended adequate diet was defined as exclusively breastfed for children <6 months, more than or equal to two food groups and breastmilk for children 6 to 9 months and more than or equal to four food groups plus breastmilk for children 10 to 23 months.[12]

Water hygiene and sanitation (WASH) facilities were further categorized into improved and unimproved sources based on WHO guidelines as described below:[13]

**IMPROVED TECHNOLOGIES**		**UNIMPROVED TECHNOLOGIES**	
Improved sources of drinking water	***Improved sanitation facilities***	***Unimproved sources of drinking water***	***Unimproved sanitation***
Piped water into dwelling, yard or plot	Flush/pour-flush to:	Unprotected dug well	Public or shared latrine
Public tap/standpipe	piped sewer system	Unprotected spring	Pit latrine without slab or open pit
Tubewell/borehole	septic tank	Vendor-provided water	Hanging toilet or hanging latrine
Protected dug well	pit (latrine)	Tanker truck water	Bucket latrine
Protected spring	Ventilated improved pit latrine	Surface water (river, stream, dam, lake, pond, canal, irrigation channel)	No facilities (so people use any area, for example a field)
Rainwater collection	Pit latrine with slab		
Bottled water*	Composting toilet		

* Bottled water is considered an "improved" source of drinking water only where there is a secondary source that is "improved".

WASH improved and unimproved sources and facilities.

Assessment of household ownership of assets such as televisions and bicycles, and housing structure were adapted from the Demographic and Health Survey (KDHS). House structure were also further categorized into improved and unimproved floor, wall and roof type as described in Lia et.al[14] (described in the box below) and cooking fuel as defined by World Bank.[15] Assets and housing structure variables were then used to derive the household asset index using principal component analysis (PCA).[16] Variables with missing data were imputed using the iterative PCA method before running PCA on complete observations.[17,18] Separate asset indices were not developed for rural and urban population. Asset quintiles were expressed in terms of quintiles with five categories depicting from the poorest to the least poor with each category representing approximately 20% of the participants.[19]

	Flooring Types	Wall Types	Roof Types
**Unimproved Materials**	Earth, sand, clay, mud<br>Dung	No wall<br>Cane/palm/trunks<br>Dirt<br>Mud and sticks<br>Tin/ cardboard/ paper/ bags<br>Thatched/straw<br>Bamboo with mud<br>Stone with mud<br>Uncovered adobe<br>Plywood<br>Cardboard<br>Reused wood<br>Trunks with mud<br>Unburnt bricks<br>Unburnt bricks with plaster<br>Unburnt bricks with mud	No roof<br>Grass/thatch/palm leaf<br>Sod<br>Straw<br>Rustic mat<br>Palm/bamboo<br>Wood planks<br>Cardboard<br>Tarpaulin, plastic
**Improved Materials**	Tablets/wood planks<br>Palm, bamboo<br>Mat<br>Adobe<br>Parquet, polished wood<br>Vinyl, asphalt strips, floor mat,<br>Linoleum<br>Ceramic tiles, mosaic<br>Cement<br>Carpet<br>Stone<br>Bricks	Cement<br>Stone with lime/cement<br>Bricks<br>Cement blocks<br>Covered adobe<br>Wood planks/shingles<br>Burnt bricks with cement	Metal<br>Wood<br>Calamine/cement fiber<br>Ceramic tiles<br>Cement<br>Roofing shingles<br>Asbestos/slate roofing sheets

Improved and unimproved floor, wall and roof type.

Means of travel to hospital was further collapsed to a binary variable either using bus/ambulances/train/car and walking/using motorbike/ rickshaw/tuktuk. Travel cost to hospital was converted to US dollars using individual country historical exchange (average) rate for each year of admission to hospital (2016-*2019)*[20,21].

During household visit GPS coordinates of a participant was taken. Euclidian distances to the nearest health facilities and to the study hospital were calculated. Health facilities locations were mined from secondary sources as shown below.

Country	Source of Health Facility Location Data
Bangladesh (Matlab and Dhaka)	https://data.humdata.org/dataset/bangladesh-healthsites
Pakistan (Karachi)	https://data.humdata.org/dataset/pakistan-healthsites
African Sites (Kilifi, Nairobi, Migori, Kampala, Blantyre, Banfora)	https://data.humdata.org/dataset/health-facilities-in-sub-saharan-africa/resource/52e95479-b85a-4cbb-b9c4-8783ff0c9713

A raster file with population density at 1 km^2 was downloaded from https://www.worldpop.org/geodata/summary?id=24776 . Point pattern analysis was done to extract population densities for participant using GPS coordinates.

Clinical definitions

- SIRS – Systemic Inflammatory Response Syndrome was defined in accordance with the International Consensus Conference on Paediatric Sepsis,[22] and includes: The presence of at least two of the following four criteria; heart rate low (<90) or high (>180)/min; temperature low (<36°C) or high (≥38·5°C); respiratory rate high (>34 breaths per minute) and WBC low (<5 $x10^9$/l) or high (>17·5 $x10^9$/l).
- Severe pneumonia – Defined by using the WHO (2013) guideline; cough/difficulty breathing with either central cyanosis or oxygen saturation <90% or lower chest wall indrawing or inability to drink/breast fed/vomiting everything or impaired consciousness.[23]

- Diarrhoea – Defined by using the WHO (2013) guideline; passage of at least three loose or watery stools in a 24hrs period.[23]
- Hypoglycaemia and hyperglycaemia were defined as blood glucose <3mmol/L and >10mmol/L respectively.
- Malaria – Defined as positive rapid Malaria test (CareStart HRP2/pLDH).
- Anaemia – Defined following WHO guidelines as: none (haemoglobin >11g/dl), mild (haemoglobin ≥10 to 11g/dl), moderate (haemoglobin ≥7 to <10g/dl) and severe (haemoglobin <7g/dl).[24]
- Dehydration – Defined by the Integrated Management of Childhood Illness criteria: Some (two of: restless/irritable, sunken eyes, drinks eagerly/thirsty, skin pinch goes back slowly), severe (lethargic/unconscious, sunken eyes, not able to drink or drinking poorly, skin pinch goes back very slowly)[23]
- Tuberculosis – Defined as on TB treatment
- Children reported to have been born with weight <2·5kg or premature (gestational age <37 weeks) were classified as having been born small for gestational age (SGA)
- Discharged against medical advice was defined as leaving hospital against medical advice or absconding from hospital.

Laboratory procedures and definitions

All children enrolled to the CHAIN cohort had a complete blood count (done at hospital laboratories), and HIV, malaria and glucose rapid tests done at enrolment. Children who were known to be HIV positive as shown in the child's health record book were not tested for HIV rapid tests. All caregivers were also offered an HIV rapid test. Sample collection area was cleaned thoroughly using an alcohol swab and dried. Blood were obtained at the time of cannulation, blood draw, through a heel or finger prick and used for the tests. All tests were done as per manufacturer's instructions while adhering to high biosafety standards and use of personal protective equipment. All test kits were used before expiry dates.

Glucose
OneTouch glucometer was used for glucose testing. Blood was applied on the glucometer test strip immediately after collection. The confirmation window was given time to fill up completely. The blood glucose reading was shown in the display window after 5-10 seconds and recorded on the Case Report Form (CRF).

Malaria Rapid Test
Malaria testing was done using CARESTART or SD Bioline Ag Pf-Pan rapid test kits depending on what was available on the site. Blood was dropped onto the round specimen well marked "S" for specimen. Four drops of diluent were added, and results read after 15 minutes. Results were interpreted based on manufacturer's instructions for the two kits used. All invalid results were repeated using a new kit.

HIV testing
All the CHAIN sites used Alere 2, Determine HIV 1 and 2 or Uni-gold HIV 1 and 2 rapid tests for HIV testing. The test was explained clearly to all care givers/participants and any positive results were kept confidential, and the family referred to the HIV service. The rapid test strip was prepared by opening and placing it on a flat surface. Blood was applied on the absorbent tip of the strip after collection. This was followed by applying 3 drops of buffer solution. Results were interpreted after 15 minutes. All tests were expected to have a line in the control section. If the line in the patient section was visible, then the test was interpreted as positive. If there was no line in the patient section, the test was negative. The test was repeated if the results were invalid. The results were shared with the caregiver and local guidelines followed for any positive results. Positive had PCR confirmatory testing according to national guidelines.

Causes of deaths

Causes of death were assigned by two independent paediatricians using all available information including all study CRF records, lab results and verbal. The procedure was the same at all sites.

Data management

Data Processing

Since the study was powered by a network of nine sites across six different countries, harmonization of the data entry process was critical for uniformity. The study was preceded by a normalized design that had factored the longitudinal set up of the study with repeating instruments to avoid anomalies associated with data integrity, i.e., insert and update anomalies. The data entry systems were hosted centrally in Nairobi and published to all sites via a secure internet gateway. The electronic database, based on REDCap system,[25] contained validations that were built into the forms. Checks for eligibility, data ranges and limits, date formats and validity, and enforcement of required fields were all made at the database level. At the site level, a manual verification step was implemented where a second clinician checked the paper CRF's before data entry into electronic database. Backlogs were monitored from a central reporting application that was published so that all sites could see their progress. This approach avoided many of the problems associated with multiple databases, where migration and merging tasks cause data consistency bottlenecks.

Data cleaning was done continuously throughout the study period. The central data management team built a visualization application and hosted it on the network for all sites to access. Queries were written and updated on the application which refreshed every 30 minutes and posted identified anomalies automatically to the dashboard application. All checks generated queries which were posted on the dashboard visualization application and sites were asked to confirm and resolve them. An additional task management application was also utilized to provide bi-directional communication on the queries, and this helped with visibility of how the queries were being resolved. This provided the much-needed insights into the data as it was being entered hence query resolution cycles were efficient. For instance, laboratory samples' data detailing timing, such as time taken to process and store samples, benefited from this process and the coordination team was able to point out violations of target times early. Clinical data were treated in a similar way. Through the app, it was possible to see follow up visits and if they had happened within target window periods as well as at variable level if the data entered was in line with the protocol.

During the study, final data curation activities were initiated to support preliminary analyses as they became due. Each data curation sprint took two to three weeks of analysis and query resolution at sites and culminated in a release of a dataset that could be used for analysis. Each release of the data was versioned and marked and placed in a read only environment for future reference. Cleaning and analysis scripts were archived at a secure shared folder in Microsoft OneDrive to allow replication. Overall, nine versions were created and finalize between 5th November 2018 and 14th September 2020.

Cleaning criteria were devised, and final cleaning executed according to statistical analysis plan. Notably, continuous variables were mapped and compared longitudinally to identify systematic issues. Missing data were queried, categorical variables were compared for consistency, and related clinical and laboratory data was correlated to identify outliers and dissimilarities. Transformations were also made on the data including variables renaming for ease of readability, calculation of processed fields based on clinical and laboratory definitions of conditions for onward analysis, and creation of flat files, including spreading of multiple instances of the same subject to create a one-record per subject structure, as well as gathering to create a deep structure i.e., multiple records per subject.

Missing data

Social, household and caregiver characteristics were not collected within 6-hours of admission and therefore these data were not available for deaths before six hours of admission. Similarly, all inpatient deaths were missing home GPS coordinates that were collected after discharge. These data were assumed not to be missing at random and were therefore excluded in the first 30 days mortality analysis but were included in the post-discharge analysis. Missing data at admission and discharge, and how they were handled in the analysis are shown in the **Table S1**.

Table S1. List of variables with missing data and methods of imputation.

Variable	Proportion missing N (%)	Imputation method
HIV	76 (2·5)	`Not tested' category was added in the analysis as a separate group.
Admission Blood glucose	59 (1·9)	Predicted blood glucose were estimated after a linear regression of the blood glucose with age, site and sex stratified by the 3 enrolment strata. Missing values were replaced with the mean predicted values in each group.
Admission height-for-age z-scores	10 (0·3)	Predicted height-for-age z-score (HAZ) were estimated after a linear regression of the HAZ with age, site and sex stratified by the 3 enrolment strata. Missing values were replaced with the mean predicted values in each group.
Birth size[a]	40 (1·4)	Predicted probability were estimated after a logistic regression of each variable with age, site and sex stratified by the 3 enrolment strata. Since these were binary variables, missing values were replaced by zero category (attribute not present) if the mean predicted values <0.5 and one category (attribute present) if the mean predicted values ≥0.5 in each group.
Recommended appropriate diet[a]	10 (0·4)	
Travel cost[a]	56 (1·9)	Predicted travel cost were estimated after a linear regression of the travel cost with age, site and sex stratified by the 3 enrolment strata. Missing values were replaced with the mean predicted values in each group.
Household GPS coordinates	3 (0·1)	Replaced with the median value in the same nutrition strata and within the same site.
Population density	3 (0·1)	Replaced with the median value in the same nutrition strata and within the same site.
Water availability[a]	6 (0·2)	Predicted probability were estimated after a logistic regression of each variable with age, site and sex stratified by the 3 enrolment strata. For binary variables, missing values were replaced by zero category (attribute not present) if the mean predicted values <0.5 and one category (attribute present) if the mean predicted values ≥0.5 in each group.
Type of toilet[a]	1 (0.03)	
Mother mental health[a]	34 (1·2)	
Mother sick[a]	13 (0·5)	
Mother working[a]	30 (1·0)	
Caregiver education level[a]	17 (0·6)	
Biological mother as primary caregiver[a]	17 (0·6)	

[a]These variables were excluded from the 30 day analysis; therefore, imputation was done on the participants included in the post-discharge models only.

Statistical methods

Baseline characteristics at the time of recruitment to the study were summarized as: N and proportions for categorical variables and means/medians and standard deviation/interquartile range for continuous variables.

Total participants completing study as planned and those lost-to-follow-up/withdrawals were reported per enrolment strata, their incidence rates and rates ratio with the NW group as the reference adjusted for recruiting site, age and sex. Follow-up time in the study was calculated and reported as child-months.

Deaths overall, during index admission and post-discharge deaths were compared between strata by calculating risk ratios adjusted for sex, age and site, inverse probability weighted for the deliberate over-sampling of higher-risk anthropometric strata, and differential loss to follow-up across sites and strata (see below).

For the post-discharge analysis, time-at-risk began at the index discharge and ended 180 days later or date of death or lost-to-follow-up (LTFU)/withdrawal.

Because of the non-proportional stratified sampling in this study, we created sampling weights (3 weights for the 3 strata) proportion to inverse of sampling fraction of the respective group from a typical hospital admission in Africa and south Asia. Hospital Paediatric admission surveillance data (for children 2 to 23 months old) during the period of CHAIN study from four site hospitals were used to estimate the mean proportions across the three nutrition strata. There was no ongoing Paediatric admission surveillance in the other five sites. Using the estimated proportions and the actual proportion of children recruited in CHAIN study we calculated the inverse probability of children being recruited in each nutrition strata and standardized the inverse probability by diving with the NW group probability (e**Table 2**).

Table S2. Inverse sample weights.

	Proportions admitted in each nutrition strata in CHAIN sites					Proportion recruited to CHAIN	Proportion of CHAIN enrolments to hospital admissions	Inverse probability weights (1/proportion of CHAIN enrolments to hospital admissions	Inverse selection weights (standardized by NW)
	Kilifi	Migori	Banfora	Dhaka[a]	Average				
Not wasted	65%	56%	58%	57%	59%	36·1%	0·61	1·63	1
Moderately wasted	13%	17%	16%	19%	16%	24·6%	1·54	0·65	0·40
Severely wasted or Kwashiorkor	22%	27%	26%	24%	25%	39·3%	1·57	0·64	0·39

[a]Defined using weight-for-length z-scores, other sites were defined using MUAC

The LTFU/withdrawals were systematically different across the three recruitment strata and the sites (e**Table 16** and **eTable 17**), we used the above approach to create 27 inverse probability weights (3 strata * 9 sites) of LTFU/withdrawal from the study. Since the sampling and LTFU inverse weights were independent, we created an overall weight to be used in the regression models by multiplying the two weights. All the regression models included the sampling and LTFU/withdrawals inverse weights.

The 30-day and post-discharge mortality regression models were built using two steps, starting with a *base model* including the enrolment strata as the main exposure and *a priori* confounders: age and sex (step 1). For the post-discharge regression, days of hospitalization, discharge against medical advice, and change in anthropometric strata from admission were included as additional *a priori* confounders (step 1).

Additional variables to be examined in the final multivariable regression models (step 2) were grouped into pre-conceived domains (**Table S3**). For each pre-conceived domain (except signs of illness severity at discharge), we performed confirmatory factor analysis where we estimated the domain score as a standalone latent variable using the set of underlying variables as presented in **Table S4**. Subsequently, we computed a predicted latent variable categorized into tertiles. The signs of illness severity at discharge score/tertile were calculated using the signs of illness severity at admission score/tertile, in such a way that each observed combination of the underlying variables at discharge yielded the same score/tertile as this combination at admission. If an underlying variable could theoretically go into two domains (e.g., HAZ could go into underlying medical conditions and child-level nutritional risk exposures), we chose the domain that fit best according to exploratory factor analysis, based on overall correlations, Bartlett's test, KMO, and the factor loadings.[26,27]

In the second step, these domain tertile scores were included in the final multivariable regression models, rather than the underlying individual variables. The final multivariable regression models include all the variables in the *base model* plus all the domain score (as tertiles).

Table S3. Variables included in the multivariable regression models.

	0 to 30 days	Post-discharge
**Base model variables**		
Age in months	Log CONT	Log CONT
Sex (male, female)	BIN	BIN
Nutritional status (NW, MW, SWK)	CAT	CAT
**Individual variables included in multivariable model**		
Change in anthropometric category at discharge[a]	-	CAT
Discharged against medical advice (AMA)[b]	-	BIN
Admission duration (days)	-	Log CONT
HIV status (negative, untested, exposed, infected)	CAT	CAT
**Domain scores**		
**Underlying medical conditions**		
Small size at birth size (no, yes)[c]	BIN	BIN
HAZ (<-3, -3 to -2, ≥-2)	CAT	CAT
Prior hospitalization (no, >1 month, <1 month)	CAT	CAT
Chronic conditions (none, suspected/confirmed)[d]	BIN	BIN
**Child-level nutritional risk exposures**		
Recommended appropriate diet (yes, no)	BIN	BIN
Recent weight loss (none, suspected, confirmed)	CAT	CAT
Poor feeding (no, yes)	BIN	BIN
**Signs of illness severity at admission**		
SIRS (no, yes)	BIN	BIN
Respiratory distress (none, moderate, severe)	CAT	CAT
Circulation (none, some signs, all signs)	CAT	CAT
Conscious level (A, VPU)	BIN	BIN
Dehydration (none, some, severe)	CAT	CAT
Blood glucose (normal, abnormal)	BIN	BIN
Severe anaemia (no, yes)	BIN	BIN
**Signs of illness severity at discharge**		
SIRS (no, yes)	BIN	BIN
Respiratory distress (none, moderate, severe)	CAT	CAT
Circulation (none, some signs)	BIN	BIN
Dehydration (none, some, severe)	CAT	CAT
Severe anaemia (no, yes)	BIN	BIN
**Access to health care**		
Distance to the nearest health facility (km)	Log CONT	Log CONT
Means of travel to hospital	BIN	BIN
Travel cost	CAT	CAT
Travel time	CAT	CAT
**Household-level exposures**		
Assets quintiles	CAT	CAT
Food insecurity (low, medium, high)	CAT	CAT
Type of toilet (improved, not improved)	BIN	BIN
Water availability (yes, no)	BIN	BIN
**Caregiver characteristics**		
Biological mother as primary caregiver (yes, no)	BIN	BIN
Caregiver education level	CAT	CAT
Mother mental health	CAT	CAT
Mother sick (no, yes)	BIN	BIN
Mother working	CAT	CAT

Abbreviations: NW; Not wasted, MW; Moderate wasting, SWK; Severe wasting/Kwashiorkor, Log CONT; natural logarithm of Continuous variable, BIN; Binary, CAT; Categorical, HAZ; Length for age z-score, SIRS; systemic Inflammatory Response Syndrome. [a]change in nutritional status at discharge was defined as no change (no change in the NW, MW, SWK groups), improved (moved from SWK to MW/NW or from MW to NW) and worsened (moved from NW to MW/SWK or from MW to SWK. [b]AMA was defined as leaving hospital against medical advice or absconding. [c]Reported birth weight <2.5kg or born premature (before 37 weeks of gestation) [d]Chronic conditions included diagnosis of thalassemia, known TB, cerebral palsy, sickle cell disease or congenital cardiac disease at admission or discharge.

Table S4. Confirmatory Factor Analysis of Exposure Domains.

	factor loading	95% CI	*P*
Signs of illness severity at admission →			
Blood glucose	0·56	0·38-0·73	<·001
Dehydration	1·04	0·81-1·27	<·001
Reduced consciousness	2·05	1·37-2·72	<·001
Respiratory distress	1	(constrained)	
Severe anaemia	0·08	-0·04-0·20	0·20
Shock	0·51	0·37-0·65	<·001
SIRS	0·71	0·55-0·87	<·001
Underlying medical conditions →			
Chronic conditions	0·45	0·21-0·68	<·001
HAZ	2·24	1·04-3·43	<·001
Prior hospitalization	0·42	0·28-0·57	<·001
Small size at birth	1	(constrained)	
Child-level nutritional risk exposures →			
Recommended appropriate diet	0·41	0·21-0·60	<·001
Poor feeding	0·76	0·34-1·18	<·001
Recent weight loss	1	(constrained)	
Caregiver characteristics →			
Biological mother as primary caregiver	1·95	0·61-3·29	0
Caregiver education level	0·53	0·08-0·99	0·02
Mother mental health	1	(constrained)	
Mother sick	-0·84	-1·55 to -0·12	0·02
Mother working	2·54	0·22-4·86	0·03
Household-level exposures →			
Asset quintiles	0·54	0·28-0·79	<·001
Food insecurity	0·14	0·08-0·19	<·001
Type of toilet	1	(constrained)	
Water availability	0·17	0·10-0·24	<·001
Access to health care →			
Distance to the nearest health facility	0·43	0·36-0·50	<·001
Means of travel to hospital	0·55	0·42-0·68	<·001
Travel cost	3·89	2·69-5·08	<·001
Travel time	1	(constrained)	

Notes: Confirmatory Factor Analysis results, where each domain was estimated separately as a latent variable that gives rise to the observed underlying measures of the respective domain. The more intense the colour the stronger the factor loading of the respective domain. The strongest factor loading of a domain is the best measure of that domain. The constrained factor loading (set to 1) in each domain was chosen using exploratory factor analysis. The signs of illness severity at discharge domain was calculated using the signs of illness severity at admission domain score, such that the similar combinations of underlying measures between admission and discharge result in similar domain scores at admission and discharge.

To be able to account for the inverse sampling and LTFU weights and control for the recruiting site shared unobserved characteristics and lack of independence of observations within sites, we used parametric multilevel survival regression models with site as a random effect component. Schoenfeld residuals test was used to test for proportional hazard assumption for variables included in the models (no violation of proportional hazard assumption was noted). We assessed four parametric probability survival distributions on their fit to our data using the Akaike information criterion (AIC), the Bayesian Information Criterion (BIC), Log likelihood and Cox–Snell residuals. The distribution with the lowest AIC and BIC provides the best fit and was thus selected.[28] We also visually assessed the four parametric distribution by plotting the model-based cumulative hazards against the predicated Cox-Snell residuals and selected

the one closest to the diagonal line. Weibull probability distribution fitted our data as shown in the **Table S5** and Cox-Snell residuals plots (**Figure S3**).

Table S5. Summary of Information criteria used to select parametric survival regression model.

Information criteria	Exponential	Weibull	Lognormal	Loglogistic	Best model
AIC	3830·9	3320·7	3386·1	3406·2	Weibull
BIC	3867·1	3363·0	3422·3	3442·4	Weibull
Log likelihood	-1909·4	-1653·4	-1687·1	-1697·1	Weibull
Abbreviations: AIC; Akaike information criterion, BIC; Bayesian Information Criterion					

We therefore used a multilevel mixed-effects parametric survival regression models with a Weibull distribution, site as random effect component including sampling and LTFU weights and reported adjusted Hazard Ratios (aHR) with 95% Confidence intervals. The final multivariable model goodness-of-fit was assessed using the area under receiver operating characteristic curves (AUC) and internally validated through bootstrapping using 1000 resampling with replacement.[29]

Sensitivity Analyses

As sensitivity analysis, multivariable regression models were performed without sampling weights and visually compared with the models accounting for sampling weights. Because the risk of mortality among severely wasted and kwashiorkor children could be different, we performed separate regression model with four anthropometric strata at admission: NW, MW, SW and Kwashiorkor. We also compared survival multivariable regression models using different hypothetical sampling weights (High: SWK=30%, MW=24% and NW=46% and Low: SWK=10%, MW=8% and NW=82%). We explored effect modification of HIV status and SIRS on the effect of nutrition status on mortality using likelihood ratio test and examined mortality by continuous MUAC by admission signs of illness severity strata.

Structural Equation Modelling

To investigate the pathways leading to mortality, we applied Structural Equation Modelling (SEM). The conceptual framework for the determinants of mortality in children in **Figure S1** formed the basis for building our empirical SEM. To explore the relationships between the underlying and immediate determinants, a survival SEM model was built according to Figure 2a and 2b, respectively, using the predicted latent scores as domain variables (**Table S4**). Survival within the generalized SEMs was modelled using the Weibull distribution. Predicted latent variables were fitted using ordinal logit models, and abnormal discharge was fitted using a Bernoulli logit model. Each outcome within the SEM was adjusted for age and sex and was modelled with a random intercept at the site level. Models were weighted using sampling and loss to follow up weights as per the survival analysis. Goodness-of-fit was assessed using the bootstrapped AUC, with 1,000 replications.

Supplementary results

Table S6. Enrolment by site.

	Started Enrolling	Last Follow up	Number Enrolled			
			**NW**	**MW**	**SWK**	**Total**
Kilifi County Hospital - Kenya	15Nov2016	06Aug2019	117	52	76	245
Mbagathi Hospital - Kenya	28Nov2016	05Aug2019	88	81	110	279
Migori County Hospital - Kenya	19Jan2016	11Jul2019	106	51	123	280
Mulago National Referral Hospital -Uganda	16Nov2016	26Aug2019	131	109	236	476
Queen Elizabeth Central hospital - Malawi	12Jan2017	01Aug2019	174	52	107	333
Dhaka Hospital - Bangladesh	17Jan2017	06Sep2019	125	109	160	394
Matlab Hospital - Bangladesh	29Jan2017	22Aug2019	95	123	96	314
Karachi Civil Hospital - Pakistan	11Feb2017	19Aug2019	134	75	140	349
Banfora Regional Referral Hospital – Burkina Faso	15Jan2018	05Sep2019	150	111	170	431
Total	15Nov2016	06Sep2019	1120	763	1218	3101

Participant characteristics at hospital admission by site

Table S7. Kilifi County Hospital, Kenya baseline characteristics.

	NW (N=117)	MW (N=52)	SWK (N=76)	Site population (N=245)
Age — months median (IQR)	11·9 (7·7–17·5)	10·5 (6·1–16·8)	12·7 (6·9–17·1)	12·0 (6·9–17·2)
Sex (female) — no. (%)	49 (42)	22 (42)	35 (46)	106 (43)
Distance to study hospital (Km) median (IQR)	19·0 (8·31–28·5)	30·4 (15·7–35·9)	32·3 (17·4–39·1)	22·7 (11·2–36·3)
SIRS — no. (%)	50 (43)	25 (48)	35 (46)	110 (45)
Severe Pneumonia — no. (%)	38 (32)	22 (42)	18 (24)	78 (32)
Diarrhoea — no. (%)	30 (26)	20 (38)	33 (43)	83 (34)
Malaria (RDT positive) — no. (%)	21 (18)	6 (12)	5 (6·6)	32 (13)
Severe anaemia — no. (%)[a]	12 (10)	7 (13)	7 (9·2)	26 (11)
Abnormal blood glucose — no. (%)[b]	6 (5·1)	3 (5·8)	11 (14)	20 (8·2)
HIV — no. (%)				
Negative	96 (82)	46 (88)	56 (74)	198 (81)
Untested	17 (15)	3 (5·8)	4 (5·3)	24 (9·8)
HIV infected	0	1 (1·9)	11 (14)	12 (4·9)
HIV exposed	4 (3·4)	2 (3·9)	5 (6·6)	11 (4·5)
Chronic conditions — no. (%)[c]	10 (8·6)	8 (15)	13 (17)	31 (13)
Assets index — no. (%)				
Quintile 1 (Lowest)	50 (43)	29 (56)	36 (47)	115 (47)
Quintile 2	34 (29)	5 (9·6)	17 (22)	56 (23)
Quintile 3	12 (10)	10 (19)	11 (14)	33 (13)
Quintile 4	12 (10)	4 (7·7)	8 (11)	24 (9·8)
Quintile 5 (Highest)	9 (7·7)	4 (7·8)	4 (5·3)	17 (6·9)
Household food insecurity — no. (%)				
Low	71 (61)	24 (46)	39 (51)	134 (55)
Medium	33 (28)	16 (31)	15 (20)	64 (26)
High	13 (11)	12 (23)	22 (29)	47 (19)
Distance to the nearest health facility (km)	2·0 (1·3–3·7)	1·7 (1·1–2·5)	2·2 (1·4–3·0)	2·1 (1·3–3·4)
Means of travel to hospital — no. (%)				
Bus/ambulance/car/train	70 (60)	40 (77)	61 (80)	171 (70)
Walking/Motorcycle/Tukutuku/rickshaw	47 (40)	12 (23)	15 (20)	74 (30)
Travel cost — no. (%)				
<1 US dollar	49 (42)	14 (27)	30 (39)	93 (38)
1 to 5 US dollars	56 (48)	35 (67)	40 (53)	131 (53)
≥ 5 US dollars	12 (10)	3 (5·8)	6 (7·9)	21 (8·6)
Travel time — no. (%)				
< 1 hour	56 (48)	14 (27)	25 (33)	95 (39)
1 to 2 hours	46 (39)	29 (56)	30 (39)	105 (43)
≥ 2 hours	15 (13)	9 (17)	21 (28)	45 (18)

Abbreviation: SIRS; Systemic inflammatory response syndrome, IQR; Interquartile range.

[a]Severe anaemia: <7•0 g/dL,

[b]Abnormal blood glucose defined as blood glucose <3 or 10mmol/l,

[c]Chronic conditions includes thalassemia, cerebral palsy, sickle cell disease, congenital cardiac disease and known TB (on treatment)

Table S8. Mbagathi Hospital, Kenya baseline characteristics.

	NW (N=88)	MW (N=81)	SWK (N=110)	Site population (N=279)
Age — months median (IQR)	11·5 (7·0–16·3)	9·6 (5·8–14·2)	9·9 (6·6–13·6)	10·5 (6·3–14·3)
Sex (female) — no. (%)	28 (32)	38 (47)	62 (56)	128 (46)
Distance to study hospital (Km) median (IQR)	7·2 (4·2–10·3)	7·8 (3·9–11·7)	8·0 (5·4–10·9)	7·8 (4·6–10·9)
SIRS — no. (%)	29 (33)	40 (49)	50 (45)	119 (43)
Severe Pneumonia — no. (%)	49 (57)	49 (60)	44 (40)	142 (51)
Diarrhoea — no. (%)	32 (36)	37 (46)	51 (46)	120 (43)
Malaria (RDT positive) — no. (%)	8 (9·1)	5 (6.2)	2 (1·8)	15 (5·4)
Severe anaemia — no. (%)[a]	7 (8·0)	12 (15)	12 (11)	31 (11)
Abnormal blood glucose — no. (%)[b]	8 (9·1)	7 (8·6)	16 (15)	31 (11)
HIV — no. (%)				
Negative	80 (91)	75 (93)	94 (85)	249 (89)
Untested	3 (3·4)	1 (1·2)	3 (2·7)	7 (·5)
HIV infected	1 (1·1)	2 (2·5)	7 (6·4)	10 (3·6)
HIV exposed	4 (4·6)	3 (3·7)	6 (5·5)	13 (4·7)
Chronic conditions — no. (%)[c]	2 (2·3)	5 (6·2)	3 (2·7)	10 (3·6)
Assets index — no. (%)				
Quintile 1 (Lowest)	0	2 (2·5)	0	2 (0·7)
Quintile 2	6 (6·8)	3 (3·7)	11 (10)	20 (7·2)
Quintile 3	19 (22)	20 (25)	35 (32)	74 (27)
Quintile 4	45 (51)	28 (35)	40 (36)	113 (41)
Quintile 5 (Highest)	18 (20)	28 (35)	24 (22)	70 (25)
Household food insecurity — no. (%)				
Low	40 (45)	37 (46)	37 (34)	114 (41)
Medium	37 (42)	31 (38)	48 (44)	116 (42)
High	11 (12)	13 (16)	25 (23)	49 (17)
Distance to the nearest health facility (km)	0·3 (0·2–0·6)	0·4 (0·2–0·6)	0·4 (0·2–0·6)	0·4 (0·2–0·6)
Means of travel to hospital — no. (%)				
Bus/ambulance/car/train	72 (82)	72 (89)	99 (90)	243 (87)
Walking/Motorcycle/Tukutuku/rickshaw	16 (18)	9 (11)	11 (10)	36 (13)
Travel cost — no. (%)				
<1 US dollar	45 (51)	38 (47)	51 (46)	134 (48)
1 to 5 US dollars	38 (43)	36 (44)	52 (47)	126 (45)
≥ 5 US dollars	5 (5·7)	7 (8·6)	7 (6·4)	19 (6·8)
Travel time — no. (%)				
< 1 hour	23 (26)	23 (28)	30 (27)	76 (27)
1 to 2 hours	46 (52)	37 (46)	45 (41)	128 (46)
≥ 2 hours	19 (22)	21 (26)	35 (32)	75 (27)

Abbreviation: SIRS; Systemic inflammatory response syndrome, IQR; Interquartile range.

[a]Severe anaemia: <7•0 g/dL,

[b]Abnormal blood glucose defined as blood glucose <3 or 10mmol/l,

[c]Chronic conditions includes thalassemia, cerebral palsy, sickle cell disease, congenital cardiac disease and known TB (on treatment)

Table S9. Migori County Hospital, Kenya baseline characteristics.

	NW (N=106)	MW (N=51)	SWK (N=123)	Site population (N=280)
Age — months median (IQR)	9·8 (5·1–15·0)	9·2 (6·9–13·4)	12·7 (6·5–18·0)	10·6 (6·4–16·5)
Sex (female) — no. (%)	36 (34)	25 (49)	60 (49)	121 (43)
Distance to study hospital (Km) median (IQR)	19·6 (5·2–28·5)	20·9 (12·9–26·9)	21·8 (15·2–31·5)	21·0 (12·2–28·6)
SIRS — no. (%)	46 (43)	26 (51)	52 (42)	124 (44)
Severe Pneumonia — no. (%)	15 (14)	6 (12)	11 (8·9)	32 (11)
Diarrhoea — no. (%)	49 (46)	29 (57)	68 (55)	146 (52)
Malaria (RDT positive) — no. (%)	34 (32)	16 (31)	17 (14)	67 (24)
Severe anaemia — no. (%)[a]	22 (21)	9 (18)	31 (25)	62 (22)
Abnormal blood glucose — no. (%)[b]	15 (14)	7 (14)	16 (13)	38 (14)
HIV — no. (%)				
Negative	87 (82)	39 (76)	78 (63)	204 (73)
Untested	1 (0·9)	1 (2·0)	1 (0·8)	3 (1·1)
HIV infected	2 (1·9)	1 (2·0)	21 (17)	24 (8·6)
HIV exposed	16 (15)	10 (20)	23 (19)	49 (18)
Chronic conditions — no. (%)[c]	2 (1·9)	0	2 (1·6)	4 (1·4)
Assets index — no. (%)				
Quintile 1 (Lowest)	58 (55)	26 (51)	81 (66)	165 (59)
Quintile 2	24 (23)	13 (25)	22 (18)	59 (21)
Quintile 3	15 (14)	7 (14)	13 (11)	35 (13)
Quintile 4	8 (7·6)	4 (7·8)	6 (4·9)	18 (6·4)
Quintile 5 (Highest)	1 (0·9)	1 (2·0)	1 (0·8)	3 (1·1)
Household food insecurity — no. (%)				
Low	56 (53)	21 (41)	38 (31)	115 (41)
Medium	35 (33)	16 (31)	42 (34)	93 (33)
High	15 (14)	14 (27)	43 (35)	72 (26)
Distance to the nearest health facility (km)	1·6 (0·8–3·0)	2·0 (1·2–3·4)	2·1 (1·3–2·9)	2·0 (1·1–3·0)
Means of travel to hospital — no. (%)				
Bus/ambulance/car/train	42 (40)	16 (31)	49 (40)	107 (38)
Walking/Motorcycle/Tukutuku/rickshaw	64 (60)	35 (69)	74 (60)	173 (62)
Travel cost — no. (%)				
<1 US dollar	27 (25)	18 (35)	25 (20)	70 (25)
1 to 5 US dollars	77 (73)	32 (63)	94 (76)	203 (73)
≥ 5 US dollars	2 (1·9)	1 (2·0)	4 (3·3)	7 (2·5)
Travel time — no. (%)				
< 1 hour	52 (49)	26 (51)	50 (41)	128 (46)
1 to 2 hours	42 (40)	20 (39)	61 (50)	123 (44)
≥ 2 hours	12 (11)	5 (9·8)	12 (9·8)	29 (10)

Abbreviation: SIRS; Systemic inflammatory response syndrome, IQR; Interquartile range.

[a]Severe anaemia: <7•0 g/dL,

[b]Abnormal blood glucose defined as blood glucose <3 or 10mmol/l,

[c]Chronic conditions includes thalassemia, cerebral palsy, sickle cell disease, congenital cardiac disease and known TB (on treatment)

Table S10. Mulago National Referral Hospital, Uganda baseline characteristics.

	NW (N=131)	MW (N=109)	SWK (N=236)	Site population (N=476)
Age — months median (IQR)	10·6 (7·5–15·8)	11·0 (8·3–13·8)	12·9 (8·8–16·8)	11·8 (8·4–15·8)
Sex (female) — no. (%)	57 (44)	50 (46)	108 (46)	215 (45)
Distance to study hospital (Km) median (IQR)	5·4 (2·7–8·5)	5·2 (2·7–8·9)	7·9 (4·8–11·1)	6·5 (3·5–10·0)
SIRS — no. (%)	47 (36)	37 (34)	61 (26)	145 (30)
Severe Pneumonia — no. (%)	37 (28)	16 (15)	28 (12)	81 (17)
Diarrhoea — no. (%)	69 (53)	75 (69)	93 (39)	237 (50)
Malaria (RDT positive) — no. (%)	12 (9·2)	11 (10)	15 (6·4)	38 (8·0)
Severe anaemia — no. (%)[a]	6 (4·6)	6 (5·5)	26 (11)	38 (8·0)
Abnormal blood glucose — no. (%)[b]	7 (5·3)	8 (7·3)	22 (9·3)	37 (7·8)
HIV — no. (%)				
Negative	116 (89)	90 (83)	188 (80)	394 (83)
Untested	4 (3·0)	3 (2·8)	3 (1·3)	10 (2·1)
HIV infected	4 (3·1)	4 (3·7)	19 (8·1)	27 (5·7)
HIV exposed	7 (5·3)	12 (11)	26 (11)	45 (9·5)
Chronic conditions — no. (%)[c]	5 (3·8)	5 (4·6)	6 (2·5)	16 (3·4)
Assets index — no. (%)				
Quintile 1 (Lowest)	3 (2·3)	2 (1·8)	14 (5·9)	19 (4·0)
Quintile 2	10 (7·6)	18 (17)	51 (22)	79 (17)
Quintile 3	53 (40)	42 (39)	92 (39)	187 (39)
Quintile 4	51 (39)	32 (29)	67 (28)	150 (32)
Quintile 5 (Highest)	14 (11)	15 (14)	12 (5·1)	41 (8·6)
Household food insecurity — no. (%)				
Low	59 (45)	40 (37)	92 (39)	191 (40)
Medium	59 (45)	50 (46)	96 (41)	205 (43)
High	13 (9·9)	19 (17)	48 (20)	80 (17)
Distance to the nearest health facility (km)	0·78 (0·45–1·47)	0·81 (0·45–1·46)	1·08 (0·64–1·66)	1·0 (0·5–1·6)
Means of travel to hospital — no. (%)				
Bus/ambulance/car/train	66 (50)	57 (52)	167 (71)	290 (61)
Walking/Motorcycle/Tukutuku/rickshaw	65 (50)	52 (48)	59 (29)	186 (39)
Travel cost — no. (%)				
<1 US dollar	61 (47)	64 (59)	106 (45)	231 (49)
1 to 5 US dollars	67 (51)	43 (39)	125 (53)	235 (49)
≥ 5 US dollars	3 (2·3)	2 (1·8)	5 (2·1)	10 (2·1)
Travel time — no. (%)				
< 1 hour	74 (56)	59 (54)	80 (34)	213 (45)
1 to 2 hours	46 (35)	41 (38)	101 (43)	188 (40)
≥ 2 hours	11 (8·4)	9 (8·3)	55 (23)	75 (16)

Abbreviation: SIRS; Systemic inflammatory response syndrome, IQR; Interquartile range.

[a]Severe anaemia: <7•0 g/dL,

[b]Abnormal blood glucose defined as blood glucose <3 or 10mmol/l,

[c]Chronic conditions includes thalassemia, cerebral palsy, sickle cell disease, congenital cardiac disease and known TB (on treatment)

Table S11. Queen Elizabeth Central Hospital, Malawi baseline characteristics.

	NW (N=174)	MW (N=52)	SWK (N=107)	Site population (N=333)
Age — months median (IQR)	12·5 (7·7–17·0)	10·7 (6·5–14·4)	12·2 (7·5–17·9)	11·9 (7·6–17·0)
Sex (female) — no. (%)	78 (45)	26 (50)	44 (41)	148 (44)
Distance to study hospital (Km) median (IQR)	6·8 (4·3–9·4)	6·5 (4·5–12·2)	7·3 (5·0–8·9)	6·9 (4·5–9·4)
SIRS — no. (%)	60 (34)	25 (40)	22 (21)	107 (32)
Severe Pneumonia — no. (%)	20 (11)	10 (19)	9 (8·4)	39 (12)
Diarrhoea — no. (%)	58 (33)	30 (58)	60 (56)	148 (44)
Malaria (RDT positive) — no. (%)	33 (19)	2 (3·9)	10 (9·4)	45 (14)
Severe anaemia — no. (%)[a]	3 (1·7)	4 (7·7)	15 (14)	22 (6·6)
Abnormal blood glucose — no. (%)[b]	14 (8·1)	1 (1·9)	5 (4·7)	20 (6·0)
HIV — no. (%)				
Negative	131 (75)	31 (60)	66 (62)	228 (68)
Untested	11 (6·3)	2 (3·9)	3 (2·8)	16 (4·8)
HIV infected	8 (4·6)	8 (15)	12 (11)	61 (18)
HIV exposed	24 (14)	11 (21)	26 (24)	28 (8·4)
Chronic conditions — no. (%)[c]	5 (2·9)	6 (12)	9 (8·4)	20 (6·0)
Assets index — no. (%)				
Quintile 1 (Lowest)	48 (28)	15 (29)	35 (33)	98 (29)
Quintile 2	48 (28)	14 (27)	33 (31)	95 (29)
Quintile 3	39 (22)	8 (15)	25 (23)	72 (22)
Quintile 4	21 (12)	8 (15)	9 (8·4)	38 (11)
Quintile 5 (Highest)	18 (10)	7 (13)	5 (4·7)	30 (9·0)
Household food insecurity — no. (%)				
Low	78 (45)	18 (35)	35 (33)	131 (39)
Medium	59 (34)	14 (27)	36 (34)	109 (33)
High	37 (21)	20 (38)	36 (34)	93 (28)
Distance to the nearest health facility (km)	2·49 (1·30–3·33)	2·66 (1·81–3·43)	2·42 (1·43–3·55)	2·5 (1·4–3·4)
Means of travel to hospital — no. (%)				
Bus/ambulance/car/train	167 (96)	52 (100)	103 (96)	322 (97)
Walking/Motorcycle/Tukutuku/rickshaw	7 (4·0)	0	4 (3·7)	11 (3·3)
Travel cost — no. (%)				
<1 US dollar	133 (76)	38 (73)	88 (82)	259 (78)
1 to 5 US dollars	40 (23)	12 (23)	14 (13)	66 (20)
≥ 5 US dollars	1 (0·6)	2 (3·9)	5 (4·7)	8 (2·4)
Travel time — no. (%)				
< 1 hour	98 (56)	32 (62)	65 (61)	195 (59)
1 to 2 hours	63 (36)	15 (29)	31 (29)	109 (33)
≥2 hours	13 (7·5)	5 (9·6)	11 (10)	29 (8·7)

Abbreviation: SIRS; Systemic inflammatory response syndrome, IQR; Interquartile range.

[a]Severe anaemia: <7•0 g/dL,

[b]Abnormal blood glucose defined as blood glucose <3 or 10mmol/l,

[c]Chronic conditions includes thalassemia, cerebral palsy, sickle cell disease, congenital cardiac disease and known TB (on treatment)

Table S12. Banfora Regional Referral Hospital, Burkina Faso baseline characteristics.

	NW (N=150)	MW (N=111)	SWK (N=170)	Site population (N=431)
Age — months median (IQR)	13.2 (9.6–18.1)	12.0 (8.3–16.9)	12.9 (7.6–18.7)	12.9 (8.5–18.1)
Sex (female) — no. (%)	56 (37)	51 (46)	78 (46)	185 (43)
Distance to study hospital (Km) median (IQR)	43.6 (9.40–66.1)	48.8 (13.7–74.0)	42.9 (15.8–65.8)	44.9 (13.6–68.0)
SIRS — no. (%)	66 (44)	55 (50)	43 (25)	164 (38)
Severe Pneumonia — no. (%)	24 (16)	23 (21)	22 (13)	69 (16)
Diarrhoea — no. (%)	64 (43)	47 (42)	74 (44)	185 (43)
Malaria (RDT positive) — no. (%)	87 (58)	63 (57)	72 (42)	222 (52)
Severe anaemia — no. (%)[a]	77 (51)	69 (62)	65 (38)	211 (50)
Abnormal blood glucose — no. (%)[b]	13 (8.7)	9 (8.1)	10 (5.9)	32 (7.4)
HIV — no. (%)				
Negative	147 (98)	109 (98)	164 (96)	420 (97)
Untested	3 (2.0)	1 (0.9)	2 (1.2)	6 (1.4)
HIV infected	0	1 (0.9)	4 (2.4)	5 (1.2)
HIV exposed	0	0	0	0
Chronic conditions — no. (%)[c]	5 (3.3)	4 (3.6)	5 (2.9)	14 (3.3)
Assets index — no. (%)				
Quintile 1 (Lowest)	49 (33)	48 (43)	71 (42)	168 (39)
Quintile 2	63 (42)	40 (36)	65 (38)	168 (39)
Quintile 3	24 (16)	13 (12)	26 (15)	63 (15)
Quintile 4	5 (3.3)	6 (5.4)	6 (3.5)	17 (3.9)
Quintile 5 (Highest)	9 (6.0)	4 (3.6)	2 (1.2)	15 (3.5)
Household food insecurity — no. (%)				
Low	137 (91)	99 (89)	144 (85)	380 (88)
Medium	12 (8.0)	11 (9.9)	24 (14)	47 (11)
High	1 (0.7)	1 (0.9)	2 (1.2)	4 (0.9)
Distance to the nearest health facility (km)	4.63 (1.03–10.5)	5.24 (1.16–11.0)	6.15 (1.36–12.6)	5.36 (1.12–11.0)
Means of travel to hospital — no. (%)				
Bus/ambulance/car/train	31 (21)	29 (26)	50 (29)	110 (26)
Walking/Motorcycle/Tukutuku/rickshaw	119 (79)	82 (74)	120 (71)	321 (74)
Travel cost — no. (%)				
<1 US dollar	17 (11)	16 (14)	16 (9.4)	49 (11)
1 to 5 US dollars	109 (73)	72 (65)	124 (73)	305 (71)
≥ 5 US dollars	24 (16)	23 (21)	30 (18)	77 (18)
Travel time — no. (%)				
< 1 hour	48 (32)	30 (27)	30 (18)	108 (25)
1 to 2 hours	48 (32)	25 (23)	48 (28)	121 (28)
≥ 2 hours	54 (36)	56 (50)	92 (54)	202 (47)

Abbreviation: SIRS; Systemic inflammatory response syndrome, IQR; Interquartile range.
[a]Severe anaemia: <7.0 g/dL,
[b]Abnormal blood glucose defined as blood glucose <3 or 10mmol/l,
[c]Chronic conditions includes thalassemia, cerebral palsy, sickle cell disease, congenital cardiac disease and known TB (on treatment)

Table S13. Dhaka Hospital, Bangladesh baseline characteristics.

	NW (N=125)	MW (N=109)	SWK (N=160)	Site population (N=394)
Age — months median (IQR)	9·2 (6·7–13·7)	9·4 (6·4–14·3)	6·5 (3·9–10·2)	8·0 (5·5–12·6)
Sex (female) — no. (%)	41 (33)	35 (32)	74 (46)	150 (38)
Distance to study hospital (Km) median (IQR)	6·45 (4·65–9·93)	6·42 (4·07–10·1)	6·61 (4·29–9·99)	6·45 (4·22–9·98)
SIRS — no. (%)	41 (33)	36 (33)	59 (37)	136 (35)
Severe Pneumonia — no. (%)	25 (20)	9 (8·3)	23 (14)	57 (14)
Diarrhoea — no. (%)	115 (92)	108 (99)	159 (99)	382 (97)
Malaria (RDT positive) — no. (%)	1 (0·8)	0	1 (0·6)	2 (0·5)
Severe anaemia — no. (%)[a]	1 (0·8)	3 (2·8)	3 (1·9)	7 (1·8)
Abnormal blood glucose — no. (%)[b]	7 (5·6)	7 (6·4)	19 (12)	33 (8·4)
HIV — no. (%)				
Negative	125 (100)	107 (98)	160 (100)	392 (99)
Untested	0	1 (0·9)	0	1 (0·3)
HIV infected	0	0	0	0
HIV exposed	0	1 (0·9)	0	1 (0·3)
Chronic conditions — no. (%)[c]	1 (0·8)	4 (3·7)	1 (0·6)	6 (1·5)
Assets index — no. (%)				
Quintile 1 (Lowest)	0	0	0	0
Quintile 2	2 (1·6)	5 (4·6)	11 (6·9)	18 (4·6)
Quintile 3	10 (8·0)	16 (15)	29 (18)	55 (14)
Quintile 4	34 (27)	33 (30)	54 (34)	121 (31)
Quintile 5 (Highest)	79 (63)	55 (50)	66 (41)	200 (51)
Household food insecurity — no. (%)				
Low	102 (82)	77 (71)	116 (73)	295 (75)
Medium	19 (15)	19 (17)	27 (17)	65 (17)
High	4 (3·2)	13 (12)	17 (11)	34 (8·6)
Distance to the nearest health facility (km)	0·56 (0·23–1·12)	0·61 (0·28–1·12)	0·60 (0·27–1·26)	0·59 (0·26–1·15)
Means of travel to hospital — no. (%)				
Bus/ambulance/car/train	28 (22)	31 (28)	53 (33)	112 (28)
Walking/Motorcycle/Tukutuku/rickshaw	97 (78)	78 (72)	107 (67)	282 (72)
Travel cost — no. (%)				
<1 US dollar	23 (18)	29 (27)	43 (27)	95 (24)
1 to 5 US dollars	96 (77)	75 (69)	111 (69)	282 (72)
≥ 5 US dollars	6 (4·8)	5 (4·6)	6 (3·8)	17 (4·3)
Travel time — no. (%)				
< 1 hour	35 (28)	32 (29)	49 (31)	116 (29)
1 to 2 hours	62 (50)	56 (51)	63 (39)	181 (46)
≥2 hours	28 (22)	21 (19)	47 (29)	97 (25)

Abbreviation: SIRS; Systemic inflammatory response syndrome, IQR; Interquartile range.

[a]Severe anaemia: <7•0 g/dL,

[b]Abnormal blood glucose defined as blood glucose <3 or 10mmol/l,

[c]Chronic conditions includes thalassemia, cerebral palsy, sickle cell disease, congenital cardiac disease and known TB (on treatment)

Table S14. Matlab Hospital, Bangladesh baseline characteristics.

	NW (N=95)	MW (N=123)	SWK (N=96)	Site population (N=314)
Age — months median (IQR)	10·9 (7·9–15·2)	11·8 (8·2–14·8)	8·6 (6·1–11·4)	10·3 (7·7–14·1)
Sex (female) — no. (%)	34 (36)	45 (36)	53 (55)	132 (42)
Distance to study hospital (Km) median (IQR)	10·2 (5·19–18·9)	14·2 (8·96–21·8)	17·7 (13·1–26·1)	15·0 (8·4–22·4)
SIRS — no. (%)	17 (18)	18 (15)	19 (20)	54 (17)
Severe Pneumonia — no. (%)	3 (3·2)	3 (2·4)	2 (2·1)	8 (2·6)
Diarrhoea — no. (%)	72 (76)	113 (92)	92 (96)	277 (88)
Malaria (RDT positive) — no. (%)	0	0	0	0
Severe anaemia — no. (%)[a]	1 (1·1)	2 (1·6)	2 (2·1)	5 (1·6)
Abnormal blood glucose — no. (%)[b]	1 (1·1)	4 (3·3)	9 (9·4)	14 (4·5)
HIV — no. (%)				
Negative	95 (100)	123 (100)	96 (100)	314 (100)
Untested	0	0	0	0
HIV infected	0	0	0	0
HIV exposed	0	0	0	0
Chronic conditions — no. (%)[c]	1 (1·1)	0	0	1 (1·1)
Assets index — no. (%)				
Quintile 1 (Lowest)	7 (7·4)	21 (17)	13 (14)	41 (13)
Quintile 2	26 (27)	41 (33)	30 (31)	97 (31)
Quintile 3	36 (38)	32 (26)	27 (28)	95 (30)
Quintile 4	15 (16)	19 (15)	16 (17)	50 (16)
Quintile 5 (Highest)	11 (12)	10 (8·1)	10 (10)	31 (9·9)
Household food insecurity — no. (%)				
Low	95 (100)	122 (99)	94 (98)	311 (99)
Medium	0	1 (0·8)	2 (2·1)	3 (1·0)
High	0	0	0	
Distance to the nearest health facility (km)	5·19 (3·25–6·78)	5·51 (3·63–8·0)	3·68 (1·87–5·99)	5·02 (3·02–6·89)
Means of travel to hospital — no. (%)				
Bus/ambulance/car/train	2 (2·1)	5 (4·1)	2 (2·1)	9 (2·9)
Walking/Motorcycle/Tukutuku/rickshaw	93 (98)	118 (96)	94 (98)	305 (97)
Travel cost — no. (%)				
<1 US dollar	16 (17)	15 (12)	8 (8·3)	39 (12)
1 to 5 US dollars	50 (53)	59 (48)	45 (47)	154 (49)
≥ 5 US dollars	29 (31)	49 (40)	43 (45)	121 (39)
Travel time — no. (%)				
< 1 hour	38 (40)	31 (25)	16 (17)	85 (27)
1 to 2 hours	31 (33)	43 (35)	42 (44)	116 (37)
≥ 2 hours	26 (27)	49 (40)	38 (40)	113 (36)

Abbreviation: SIRS; Systemic inflammatory response syndrome, IQR; Interquartile range.

[a]Severe anaemia: <7•0 g/dL,

[b]Abnormal blood glucose defined as blood glucose <3 or 10mmol/l,

[c]Chronic conditions includes thalassemia, cerebral palsy, sickle cell disease, congenital cardiac disease and known TB (on treatment)

Table S15. Karachi Civil Hospital, Pakistan baseline characteristics.

	NW (N=134)	MW (N=75)	SWK (N=140)	Site population (N=349)
Age — months median (IQR)	10·4 (6·0–15·6)	9·2 (5·3–14·3)	7·5 (4·3–13·9)	8·6 (5·3–14·6)
Sex (female) — no. (%)	53 (40)	37 (49)	69 (49)	159 (46)
Distance to study hospital (Km) median (IQR)	3·15 (1·77–8·81)	4·06 (2·06–8·53)	3·50 (2·16–9·46)	3·43 (1·99–9·11)
SIRS — no. (%)	39 (29)	22 (29)	45 (32)	106 (30)
Severe Pneumonia — no. (%)	75 (56)	41 (55)	45 (32)	161 (46)
Diarrhoea — no. (%)	36 (27)	25 (33)	82 (59)	143 (41)
Malaria (RDT positive) — no. (%)	6 (4·5)	3 (4·0)	10 (7·1)	19 (5·4)
Severe anaemia — no. (%)[a]	6 (4·5)	11 (15)	24 (17)	41 (12)
Abnormal blood glucose — no. (%)[b]	8 (6·0)	2 (2·7)	9 (6·4)	19 (5·4)
HIV — no. (%)				
Negative	133 (99)	73 (97)	132 (94)	338 (97)
Untested	0	2 (2·7)	7 (5·0)	9 (2·6)
HIV infected	0	0	0	0
HIV exposed	1 (0·8)	0	1 (0·7)	2 (0·6)
Chronic conditions — no. (%)[c]	28 (21)	19 (25)	54 (39)	101 (29)
Assets index — no. (%)				
Quintile 1 (Lowest)	0	1 (1·3)	0	1 (0·3)
Quintile 2	2 (1·5)	2 (2·7)	0	4 (1·2)
Quintile 3	5 (3·7)	5 (6·7)	10 (7·1)	20 (5·7)
Quintile 4	30 (22)	22 (29)	46 (33)	98 (28)
Quintile 5 (Highest)	97 (72)	45 (60)	84 (60)	226 (65)
Household food insecurity — no. (%)				
Low	75 (56)	35 (47)	64 (46)	174 (50)
Medium	48 (36)	28 (37)	43 (31)	119 (34)
High	11 (8·2)	12 (16)	33 (24)	56 (16)
Distance to the nearest health facility (km)	0·45 (0·27–0·80)	0·52 (0·30–0·79)	0·50 (0·33–0·89)	0·49 (0·30–0·83)
Means of travel to hospital — no. (%)				
Bus/ambulance/car/train	27 (20)	13 (17)	22 (16)	62 (18)
Walking/Motorcycle/Tukutuku/rickshaw	107 (80)	62 (83)	118 (84)	287 (82)
Travel cost — no. (%)				
<1 US dollar	92 (67)	37 (49)	72 (51)	201 (58)
1 to 5 US dollars	40 (30)	38 (51)	64 (46)	142 (41)
≥ 5 US dollars	2 (1·5)	0	4 (2·9)	6 (1·7)
Travel time — no. (%)				
< 1 hour	102 (76)	69 (92)	102 (73)	273 (78)
1 to 2 hours	30 (22)	6 (8·0)	36 (26)	72 (21)
≥ 2 hours	1 (0·8)	0	1 (0·7)	4 (1·2)

Abbreviation: SIRS; Systemic inflammatory response syndrome, IQR; Interquartile range.

[a]Severe anaemia: <7•0 g/dL,

[b]Abnormal blood glucose defined as blood glucose <3 or 10mmol/l,

[c]Chronic conditions includes thalassemia, cerebral palsy, sickle cell disease, congenital cardiac disease and known TB (on treatment)

Loss-to-follow-up & withdrawal

Table S16. Lost-to-follow-up and withdrawal from admission to 180 days after discharge.

	N	LTFU & Withdrawals, N (%)	Rate per 1000 child-months	Incidence rate ratios (95% CI)[a]	P-value
All participants	3101	116 (3·7)	7·02 (5·84–8·43)	-	-
**Nutritional status**					
Severely wasted or Kwashiorkor	1218	28 (2·3)	4·72 (3·26–6·84)	0·73 (0·46–1·14)	0·16
Moderate wasted	763	25 (3·3)	6·03 (4·07–8·92)	0·92 (0·56–1·50)	0·73
Not wasted	1120	63 (5·6)	9·90 (7·70–12·7)	Reference	

[a] Rate ratios estimated using the Mantel-Cox method and was adjusted for sex, age and site.

Table S17. Lost-to-follow-up and withdrawals from admission to 180 days after discharge by site.

	N	Not wasted	Moderately wasted	Severely wasted or Kwashiorkor	Total LTFU & Withdrawals
Sites – N (%)					
Kilifi - Kenya	245	7 (6·0)	4 (7·7)	3 (4·0)	14 (5·7)
Mbagathi - Kenya	279	5 (5·7)	1 (1·2)	3 (2·7)	9 (3·2)
Migori - Kenya	280	1 (0·9)	0	0	1 (0·4)
Kampala - Uganda	476	1 (0·9)	4 (3·7)	2 (0·9)	7 (1·5)
Blantyre - Malawi	333	39 (22)	8 (15)	15 (14)	62 (19)*
Banfora - Burkina Faso	431	2 (1·3)	3 (2·7)	1 (0·6)	6 (1·4)
Dhaka - Bangladesh	394	0	0	1 (0·6)	1 (0·3)
Matlab - Bangladesh	314	1 (1·1)	3 (2·4)	0	4 (1·3)
Karachi -Pakistan	349	7 (5·2)	2 (2·7)	3 (2·1)	12 (3·4)

*The high LTFU from Malawi was attributable to community myths during study period alleging research blood samples were used for some religious rituals. This led to some of the study participant to withdrawal consent. The study was temporarily halted until the rumours stopped.

Follow-up time & mortality

Table S18. Follow-up time and mortality rates at pre-specified time points by enrolment strata.

	N	Deaths N (%)	1000 Child-months	Mortality rate (95% CI) per 1000 child-months[a]
**Admission to 30 days**				
Not wasted	1120	26 (2·3)	1·11	24·1 (16·5–36·4)
Moderately wasted	763	40 (5·2)	0·74	55·0 (40·5–76·5)
Severely wasted/kwashiorkor	1218	168 (14)	1·09	156 (134–183)
**Discharge to 180 days post discharge**				
Not wasted	1072	17 (1·6)	6·40	2·72 (1·72–4·57)
Moderately wasted	724	30 (4·3)	4·22	7·32 (5·16–10·7)
Severely wasted/kwashiorkor	1078	121 (11)	5·89	21·0 (17·6–25·3)
**Admission to 180 days**				
Not wasted	1120	39 (3·5)	6·56	6·05 (4·42–8·49)
Moderately wasted	763	62 (8·1)	4·27	14·9 (11·6–19·5)
Severely wasted or Kwashiorkor	1218	249 (20)	6·05	42·2 (37·0–48·3)

[a]Adjusted for inverse weights of LTFU/withdrawal

Discharge Characteristics

Table S19. Participants selected characteristics at index discharge.

	NW (N=1072)	MW (N=724)	SWK (N=1078)
**Demographics**			
Age — months median (interquartile range)	11·4 (7·5–16·3)	10·9 (7·3–14·8)	10·7 (6·41–16·2)
Gender (female) — no. (%)	410 (38)	317 (44)	512 (48)
Discharged against medical advice — no. (%)	60 (5.6)	39 (5·4)	106 (9·8)
Hospital admission duration (days) median (IQR)%	3 (2–5)	4 (2–6)	7 (4–12)
Change in undernutrition at discharge — no. (%)[a]			
No change	1034 (96)	599 (83)	814 (76)
Improved	0	90 (12)	264 (24)
Worsened	38 (3·5)	35 (4·8)	0
Acute illness at discharge			
Systemic inflammatory response syndrome (SIRS) — no. (%)	146 (14)	98 (14)	171 (16)
Severe Pneumonia — no. (%)	61 (5·7)	36 (5·0)	70 (6·4)
Any sign of shock — no. (%)	149 (14)	91 (13)	225 (21)
Neurological (AVPU >A) — no. (%)	1 (0·09)	2 (0·3)	0
Anaemia — no. (%)			
None	151 (14)	92 (13)	114 (11)
Mild	326 (30)	170 (23)	215 (20)
Moderate	522 (49)	405 (56)	659 (61)
Severe	73 (6·8)	57 (7·8)	90 (8·4)
**Anthropometry**			
Nutritional edema — no. (%)	1 (0·09)	0	44 (4·1)
MUAC (CM) — mean (sd)	13·6 ±1·0	12·0 ±0·4	10·8 ±1·3
Weight-for-length z score — mean (sd)[b]	-0·5 ±1·1	-2·1 ±1·0	-2·8 ±1·4
Weight-for-age z score — mean (sd)[b]	-1·1 ±1·1	-2·6 ±1·0	-3·8 ±1·3
Length-for-age z score — mean (sd)	-1·2 ±1·3	-2·0 ±1·3	-3·2 ±1·6

[a]Change in nutritional status at discharge was defined as no change (no change in the NW, MW, SWK groups), improved (moved from SWK to MW/NW or from MW to NW) and worsened (moved from NW to MW/SWK or from MW to SWK),

[b]Children with edema are excluded from these values.

Causes of death

Table A20-A. Attributed causes of death (all deaths).

Attributed causes of death (all deaths) Immediate cause corresponding cause "1.a" on standard WHO death certification forms. Underlying causes correspond to item "1.b" – "1.d" on WHO death certification forms.

	SWK deaths = 249 n (% of deaths)	MW deaths = 62 n (% of deaths)	NW deaths = 39 n (% of deaths)	Total deaths = 350 n (% of deaths)
Immediate Cause of Death (ICD-10 code)				
Severe Sepsis (R65·2)	95(38%)	24 (39%)	6 (15%)	125 (36%)
Pneumonia, unspecified organism (J18·9)	70 (28%)	21 (34%)	16 (41%)	107 (31%)
Diarrhoea, unspecified (R19·7)	47 (19%)	8 (13%)	3 (8%)	58 (17%)
Malaria, unspecified (B54)	7 (3%)	7 (11%)	10 (26%)	24 (7%)
Meningitis, unspecified (G03·9)	5 (2%)	0 (0%)	3 (8%)	8 (2%)
Anaemia, unspecified (D64·9)	5 (2%)	1 (2%)	0 (0%)	6 (2%)
Other[a]	20 (8%)	1 (2%)	1 (0%)	22 (6%)
Underlying Causes of Death (ICD-10 code)				
Severe protein-calorie malnutrition, unspecified (E43)[b]	209 (84%)	12 (19%)	2 (5%)	223 (64%)
HIV disease (B20)	40 (16%)	11 (18%)	1 (3%)	52 (15%)
Anaemia, unspecified (D64·9)	9 (4%)	5 (8%)	7 (18%)	21 (6%)
Severe Sepsis (R65·2)	9 (4%)	3 (5%)	3 (8%)	15 (4%)
Diarrhoea, unspecified (R19·7)	6 (2%)	3 (5%)	3 (8%)	12 (3%)
Pneumonia, unspecified organism (J18·9)	4 (2%)	5 (8%)	2 (5%)	11 (3%)
Other[c]	3 (2%)	0 (0%)	1 (3%)	4 (1%)

[a]Other (immediate): Liver failure (K72·9) = 2, Measles (B05) = 2, Poisoning (T50·901A) = 2, Renal failure (N17·9) = 1, Sickle cell disease (D57·41) = 1, Unknown (R99) = 14.

[b]Severe malnutrition defined according to WHO criteria at last study contact prior to death.

[c]Other (underlying): Down's Syndrome (Q90·0) = 1, Encephalitis (G04·8) = 1, Malaria unspecified (B54) = 2, Measles (B05) = 4, Meningitis unspecified (G03·9) =1, Tuberculosis (A15 & A18) = 2, Unknown (R99) = 2.

Table S20-B. Attributed causes of death (death during index admission).

Attributed causes of death (death during index admission) Immediate cause corresponding cause "1.a" on standard WHO death certification forms. Underlying causes correspond to item "1.b" – "1.d" on WHO death certification forms.

	SWK deaths = 128 n (% of deaths)	MW deaths = 32 n (% of deaths)	NW deaths = 22 n (% of deaths)	Total deaths = 182 n (% of deaths)
Immediate Cause of Death (ICD-10 code)				
Pneumonia, unspecified organism (J18·9)	31 (24%)	10 (31%)	10 (45%)	51 (28%)
Severe Sepsis (R65·2)	43 (34%)	7 (22%)	0 (0%)	50 (27%)
Diarrhoea, unspecified (R19·7)	36 (28%)	7 (22%)	1 (5%)	44 (24%)
Malaria, unspecified (B54)	7 (5%)	7 (22%)	9 (41%)	23 (13%)
Other[a]	11 (9%)	1 (3%)	2 (9%)	14 (8 %)
Underlying Causes of Death (ICD-10 code)				
Severe protein-calorie malnutrition, unspecified (E43)[b]	122 (95%)	7 (22%)	0 (0%)	129 (71%)
HIV disease (B20)	22 (17%)	7 (22%)	1 (5%)	30 (16%)
Anaemia, unspecified (D64·9)	8 (6%)	3 (9%)	5 (23%)	16 (9%)
Severe Sepsis (R65·2)	5 (4%)	3 (9%)	1 (5%)	11 (6%)
Diarrhoea, unspecified (R19·7)	5 (4%)	2 (6%)	3 (14%)	10 (5%)
Pneumonia, unspecified organism (J18·9)	2 (2%)	2 (6%)	2 (9%)	6 (3%)
Other[c]	3 (2%)	2 (6%)	2 (9%)	7 (4%)

[a]Other (immediate): Anaemia (D64·9) = 3, Liver failure (K72·9) = 1, Measles (B05) = 2, Meningitis unspecified (G03·9) = 3, Poisoning (T50·901A) = 2, Sickle cell disease (D57·41) = 1, Unknown (R99) = 2.

[b]Severe malnutrition defined according to WHO criteria at last study contact prior to death

[c]Other (underlying): Down's Syndrome (Q90·0) = 1, Encephalitis (G04·8) = 1, Malaria unspecified (B54) = 1, Measles (B05) = 2, Meningitis unspecified (G03·9) =1, Tuberculosis (A15 & A18) = 1.

Table S20-C. Attributed causes of death (death within 30 days of date of index admission)

Attributed causes of death (death within 30 days of date of index admission) Immediate cause corresponding cause "1.a" on standard WHO death certification forms. Underlying causes correspond to item "1.b" – "1.d" on WHO death certification forms.

	SWK deaths = 168 n (% of deaths)	MW deaths = 40 n (% of deaths)	NW deaths = 26 n (% of deaths)	Total deaths = 234 n (% of deaths)
Immediate Cause of Death (ICD-10 code)				
Pneumonia, unspecified organism (J18·9)	42 (25%)	15 (38%)	11 (42%)	68 (29%)
Severe Sepsis (R65·2)	59 (35%)	10 (25%)	1 (4%)	70 (30%)
Diarrhoea, unspecified (R19·7)	41 (24%)	7 (18%)	2 (8%)	50 (21%)
Malaria, unspecified (B54)	7 (4%)	7 (18%)	9 (35%)	23 (10%)
Meningitis, unspecified (G03·9)	4 (2%)	0 (0%)	2 (8%)	6 (3%)
Anaemia, unspecified (D64·9)	4 (2%)	1 (3%)	0 (0%)	5 (2%)
Other[a]	11 (7%)	0 (0%)	1 (4%)	12 (5%)
Underlying Causes of Death (ICD-10 code)				
Severe protein-calorie malnutrition, unspecified (E43)[b]	151 (90%)	7 (18%)	0 (0%)	158 (68%)
Anaemia, unspecified (D64·9)	8 (5%)	4 (10%)	5 (19%)	17 (7%)
Severe Sepsis (R65·2)	7 (4%)	3 (8%)	3 (12%)	13 (6%)
HIV disease (B20)	27 (16%)	7 (18%)	1 (4%)	35 (15%)
Diarrhoea, unspecified (R19·7)	6 (4%)	2 (5%)	3 (12%)	11 (5%)
Pneumonia, unspecified organism (J18·9)	4 (2%)	2 (5%)	2 (8%)	8 (3%)
Other[c]	6 (4%)	1 (3%)	2 (8%)	9 (4%)

[a]Other (immediate): Liver failure (K72·9) = 2, Measles (B05) = 2, Poisoning (T50·901A) = 2, Sickle cell disease (D57·41) = 1, Unknown (R99) = 5.

[b]Severe malnutrition defined according to WHO criteria at last study contact prior to death.

[c]Other (underlying): Down's Syndrome (Q90·0) = 1, Malaria unspecified (B54) = 1, Measles (B05) = 3, Meningitis unspecified (G03·9) =1, Tuberculosis (A15 & A18) = 1, Unknown (R99) = 2.

Table S20-D. Attributed causes of death (death after discharge from index admission)

Attributed causes of death (death after discharge from index admission) Immediate cause corresponding cause "1.a" on standard WHO death certification forms. Underlying causes correspond to item "1.b" – "1.d" on WHO death certification forms.

	SWK deaths = 121 n (% of deaths)	MW deaths = 30 n (% of deaths)	NW deaths = 17 n (% of deaths)	Total deaths = 168 n (% of deaths)
Immediate Cause of Death (ICD-10 code)				
Severe Sepsis (R65·2)	52 (43%)	17 (57%)	6 (35%)	75 (45%)
Pneumonia, unspecified organism (J18·9)	39 (32%)	11 (37%)	6 (35%)	56 (33%)
Diarrhoea, unspecified (R19·7)	11 (9%)	1 (3%)	2 (12%)	14 (8%)
Meningitis, unspecified (G03·9)	3 (2%)	0 (0%)	2 (12%)	5 (3%)
Severe protein-calorie malnutrition, unspecified (E43)[a]	4 (3%)	0 (0%)	0 (0%)	4 (2%)
Other[b]	16 (13%)	1 (3%)	1 (6%)	18 (11%)
Underlying Causes of Death (ICD-10 code)				
Severe protein-calorie malnutrition, unspecified (E43)[a]	85 (70%)	5 (17%)	2 (12%)	68 (40%)
HIV disease (B20)	18 (15%)	4 (13%)	0 (0%)	22 (13%)
Severe Sepsis (R65·2)	4 (3%)	0 (0%)	0 (0%)	4 (2%)
Pneumonia, unspecified organism (J18·9)	2 (2%)	3 10%)	0 (0%)	5 (3%)
Anaemia, unspecified (D64·9)	1 (1%)	2 (7%)	2 (12%)	5 (3%)
Other[c]	6 (5%)	1 (3%)	1 (6%)	8 (5%)

[a]Severe malnutrition defined according to WHO criteria at last study contact prior to death.

[b]Other (immediate): Anaemia unspecified (D64·9) = 3, Liver failure (K72·9) = 1, Renal failure (N17·9) = 1, Unknown (R99) = 13.

[c]Other (underlying): Diarrhoea, unspecified (R19·7) = 2, Encephalitis (G04·8) = 1, Malaria unspecified (B54) = 1, Measles (B05) = 1, Tuberculosis (A15 & A18) = 1, Unknown (R99) = 2.

Deaths by admission clinical syndrome

Table S21. Deaths by admission clinical syndrome

	NW (N=1120)	MW (N=763)	SWK (N=1218)	Risk ratio (95% CI)[a] MW vs. NW	SWK vs. NW
**Severe pneumonia at admission – N (%)**					
During index admission	15 (5·2)	16 (8·9)	39 (19)	1·60 (0·93–2·75)	3·52 (2·44–5·07)
Within 30 days	16 (5·6)	17 (9·5)	45 (22)	1·63 (1·01–2·62)	3·85 (2·41–6·15)
Post-discharge	3 (1·1)	11 (6·8)	28 (17)	5·84 (1·85–18·5)	15·5 (5·76–41·9)
**Diarrhoea at admission – N (%)**					
During index admission	8 (1·5)	21 (4·3)	87 (12)	3·31 (2·43–4·50)	8·48 (6·02–12·0)
Within 30 days	10 (1·9)	25 (5·2)	112 (16)	3·04 (2·13–4·34)	8·30 (6·21–11·1)
Post-discharge	7 (1·4)	13 (2·8)	58 (9·4)	2·21 (1·16–4·20)	6·40 (2·95–13·9)
**SIRS at admission – N (%)**					
During index admission	14 (3·5)	16 (5·6)	72 (19)	1·56 (0·97–2·52)	5·27 (3·28–8·48)
Within 30 days	14 (3·5)	20 (7·0)	87 (23)	1·96 (1·26–3·05)	6·27 (3·74–10·5)
Post-discharge	7 (1·9)	17 (6·4)	47 (15)	3·44 (2·05–5·77)	8·03 (3·12–20·7)
**Severe anaemia at admission – N (%)**					
During index admission	5 (3·7)	5 (4·1)	27 (15)	1·10 (0·77–1·57)	4·06 (1·48–11·1)
Within 30 days	6 (4·4)	8 (6·5)	35 (19)	1·39 (0·98–1·95)	4·17 (1·47–11·8)
Post-discharge	6 (4·7)	7 (5·9)	21 (13)	1·28 (0·91–1·80)	2·66 (1·07–6·59)
**Malaria at admission – N (%)**					
During index admission	9 (4·5)	7 (6·6)	10 (7·6)	1·15 (0·69–1·93)	1·59 (0·91–2·78)
Within 30 days	10 (5·0)	8 (7·6)	10 (7·6)	1·18 (0·55–2·54)	1·42 (0·75–2·69)
Post-discharge	5 (2·7)	1 (1·0)	5 (4·2)	0·29 (0·03–3·40)	1·49 (0·67–3·31)
**HIV exposed/infected at admission – N (%)**					
During index admission	2 (2·8)	7 (13)	38 (24)	4·55 (2·28–9·10)	8·61 (5·18–14·3)
Within 30 days	2 (2·8)	7 (13)	45 (28)	4·42 (2·23–8·74)	10·1 (6·35–16·0)
Post-discharge	1 (1·5)	4 (8·3)	26 (21)	5·63 (0·88–35·9)	15·2 (1·94–119)

[a]Risk ratios from multilevel log-binomial regression models adjusted for age, sex with site as random effects.

Location of post-discharge deaths

Table S22. Location of post-discharge deaths.

	N	All deaths N (%)	Place of death: Deaths during hospital readmission N (% of deaths)	Place of death: Deaths in community N (% of deaths)
All	2874	168 (5·9)	78 (46)	90 (54)
Not wasted	1072	17 (1·6)	11 (65)	6 (35)
Moderately wasted	724	30 (4·1)	17 (57)	13 (43)
Severely wasted/Kwashiorkor	1078	121 (11)	50 (41)	71 (59)

Deaths by site

Table S23. Inpatient, 30-day and post-discharge deaths stratified by site and anthropometric strata.

	Inpatient deaths			30-day deaths			Post-discharge deaths		
Sites	NW (N=22)	MW (N=32)	SWK (N=128)	NW (N=26)	MW (N=40)	SWK (N=168)	NW (N=17)	MW (N=30)	SWK (N=121)
**Kilifi - Kenya**	3 (2·6)	1 (1·9)	8 (11)	3 (2·6)	2 (3·9)	8 (11)	1 (0·9)	2 (4·1)	7 (10)
**Mbagathi- Kenya**	2 (2·3)	7 (8·6)	14 (13)	3 (3·4)	7 (8·6)	15 (14)	2 (2·4)	6 (8·1)	14 (15)
**Migori- Kenya**	5 (4·7)	8 (16)	34 (28)	5 (4·7)	10 (20)	38 (31)	5 (5·0)	7 (16)	14 (16)
**Kampala- Uganda**	2 (1·5)	4 (3·7)	23 (9·8)	2 (1·5)	5 (4·6)	28 (12)	0	3 (2·9)	21 (9·9)
**Blantyre- Malawi**	2 (1·2)	2 (3·9)	17 (16)	2 (1·2)	2 (3·9)	23 (22)	0	2 (4·4)	16 (19)
**Banfora- Burkina Faso**	6 (4·0)	8 (7·2)	19 (11)	7 (4·7)	9 (8·1)	24 (14)	3 (2·1)	4 (3·9)	15 (9·9)
**Dhaka- Bangladesh**	0	1 (0·9)	7 (4·4)	1 (0·8)	2 (1·8)	11 (6·9)	2 (1·6)	3 (2·8)	7 (4·6)
**Matlab- Bangladesh**	1 (1·1)	1 (0·8)	1 (1·0)	1 (1·1)	1 (0·8)	4 (4·2)	0	0	4 (4·2)
**Karachi -Pakistan**	1 (0·8)	0	5 (3·6)	2 (1·5)	2 (2·7)	17 (12)	4 (3·1)	3 (4·0)	23 (17)

Distribution of domain scores used in the multivariable regression analysis.

Table S24. Distribution of domain classifications by admission anthropometry.

Domains	30-day model			Post-discharge model		
	NW (1120)	MW (763)	SWK (1218)	NW (1072)	MW (724)	SWK (1078)
**Severity of illness at admission**						
Low abnormal	340 (30)	263 (34)	507 (42)	329 (31)	258 (36)	472 (44)
Mid abnormal	383 (34)	227 (30)	353 (29)	374 (35)	220 (30)	325 (30)
High abnormal	397 (36)	273 (36)	358 (29)	369 (34)	246 (34)	281 (26)
**Severity of illness at discharge**						
Low abnormal	[a]	[a]	[a]	878 (82)	618 (85)	886 (82)
Mid abnormal	[a]	[a]	[a]	173 (16)	96 (13)	153 (14)
High abnormal	[a]	[a]	[a]	21 (2·0)	10 (1·4)	39 (3·6)
**Underlying medical conditions**						
Low abnormal	584 (52)	275 (36)	190 (16)	570 (53)	261 (36)	176 (16)
Mid abnormal	424 (38)	300 (39)	358 (29)	396 (37)	279 (39)	308 (29)
Severe abnormal	112 (10)	188 (25)	670 (55)	106 (9·9)	184 (25)	594 (55)
**Child-level nutritional risk exposures**						
Low abnormal	935 (83)	421 (55)	404 (33)	893 (83)	396 (55)	366 (34)
Mid abnormal	74 (6·6)	120 (16)	124 (10)	72 (6·7)	118 (16)	116 (11)
Severe abnormal	111 (9·9)	222 (29)	690 (57)	107 (10)	210 (29)	596 (55)
**Caregiver characteristics**						
Low abnormal	458 (41)	323 (42)	410 (34)	448 (42)	315 (44)	380 (35)
Mid abnormal	347 (31)	241 (32)	423 (35)	322 (30)	227 (31)	370 (34)
Severe abnormal	315 (28)	199 (26)	385 (31)	302 (28)	182 (25)	328 (31)
**Household-level risk exposures**						
Low abnormal	408 (36)	270 (35)	367 (30)	400 (37)	259 (36)	350 (32)
Mid abnormal	345 (31)	261 (34)	423 (35)	325 (30)	249 (34)	376 (35)
Severe abnormal	367 (33)	232 (30)	428 (35)	347 (33)	216 (30)	352 (33)
**Access to health care**						
Low abnormal	419 (37)	239 (31)	376 (31)	395 (37)	223 (31)	334 (31)
Mid abnormal	369 (33)	262 (34)	403 (33)	361 (34)	253 (35)	364 (34)
Severe abnormal	332 (30)	262 (34)	439 (36)	316 (29)	248 (34)	380 (35)

The predicted scores are presented as the N and proportions of participants in each category

[a]No scores computed because the domains computation use discharge data.

Survival regression analysis: characteristics associated with death.

Table S25. Base model controlling for both sampling and LTFU weights.

	Admission to 30 days		Post-discharge	
	**HR (95% CI)**	**P-value**	**HR (95% CI)**	**P-value**
**Nutritional status**				
Not wasted	Reference		Reference	
Moderately wasted	2·45 (1·68–3·59)	<0·001	2·81 (1·64–4·82)	<0·001
Severely wasted/kwashiorkor	6. ·37 (3·90–10·4)	<0·001	7·49 (3·62–15·5)	<0·001
Age in months (Log)	0·74 (0·63–0·87)	<0·001	0·74 (0·62–0·87)	<0·001
Sex: Female	1·12 (0·85–1·47)	0·41	1·33 (0·86–2·06)	0·20
Recruitment site variance	0·26 (0·08–0·92)		0·17 (0·03–0·89)	
AUC	0·76 (0·73–0·79)		0·76 (0·73–0·80)	

Abbreviations: HR; Hazard ratios, AUC; area under receiver operating characteristics.
Hazards ratio from mixed effect Weibull survival parametric model with site as a random effect controlling for sampling and LTFU weights.

Table S26. Base model without sample weights, with LTFU weights only.

	Admission to 30 days		Post-discharge	
	**HR (95% CI)**	**P-value**	**HR (95% CI)**	**P-value**
Nutritional status				
Not wasted	Reference		Reference	
Moderately wasted	2·57 (1·76–3·76)	<0·001	2·98 (1·70–5·22)	<0·001
Severely wasted/kwashiorkor	6·55 (4·00–10·7)	<0·001	7·82 (3·68–16·6)	<0·001
Age in months (Log)	0·77 (0·65–0·91)	0·002	0·72 (0·58–0·89)	0·002
Sex				
Female	1·05 (0·86–1·27)	0·64	1·21 (0·81–1·82)	0·35
Recruitment site variance	0·35 (0·12–1·04)		0·28 (0·07–1·04)	
AUC	0·76 (0·73–0·79)		0·77 (0·74–0·80)	

Abbreviations: HR; Hazard ratios, AUC; area under receiver operating characteristics.
HR from mixed effect Weibull survival parametric model with site as a random effect and controlling for LTFU weights only.

Table S27. Base model with nutritional status defined using weight-for-length z score.

	Admission to 30 days		Post-discharge	
	**HR (95% CI)**	**P-value**	**HR (95% CI)**	**P-value**
**Nutritional status**				
Not wasted	Reference		Reference	
Moderately wasted	2·29 (1·78–2·93)	<0·001	2·34 (1·20–4·55)	0·01
Severely wasted/kwashiorkor	4·09 (2·77–6·02)	<0·001	4·26 (2·13–8·48)	<0·001
Age in months (Log)	0·65 (0·53–0·80)	<0·001	0·59 (0·46–0·74)	<0·001
Sex				
Female	1·19 (0·97–1·47)	0·09	1·43 (0·96–2·12)	0·08
Recruitment site variance	0·34 (0·12–0·97)		0·26 (0·07–0·94)	
AUC	0·74 (0·71–0·77)		0·73 (0·70–0·77)	

Abbreviations: HR; Hazard ratios, AUC; area under receiver operating characteristics.
Hazards ratio from mixed effect Weibull survival parametric model with site as a random effect controlling for sampling and LTFU weights.

Table S28. Base model with nutritional status defined using weight-for-age z score.

	**Admission to 30 days**		**Post-discharge**	
	**HR (95% CI)**	**P-value**	**HR (95% CI)**	**P-value**
**Nutritional status**				
Not underweight	Reference		Reference	
Moderate underweight	2·54 (1·63–3·96)	<0·001	1·97 (1·05–3·69)	0·03
Severe underweight/kwashiorkor	4·67 (3·10–7·04)	<0·001	4·95 (2·50–9·81)	<0·001
Age in months (Log)	0·73 (0·60–0·88)	0·001	0·66 (0·53–0·82)	<0·001
Sex				
Female	1·24 (1·01–1·51)	0·04	1·44 (0·98–2·10)	0·06
Recruitment site variance	0·44 (0·15–1·27)		0·35 (0·10–1·25)	
AUC	0·74 (0·71–0·78)		0·75 (0·72–0·79)	

Abbreviations: HR; Hazard ratios, AUC; area under receiver operating characteristics.
Hazards ratio from mixed effect Weibull survival parametric model with site as a random effect controlling for sampling and LTFU weights.

Table S29. Base model with nutritional status defined using length-for-age z score.

	**Admission to 30 days**		**Post-discharge**	
	**HR (95% CI)**	**P-value**	**HR (95% CI)**	**P-value**
**Nutritional status**				
Not stunted	Reference		Reference	
Moderate stunted	2·05 (1·34–3·13)	0·001	1·41 (0·89–2·23)	0·14
Severe stunted	2·80 (1·78–4·40)	<0·001	3·52 (2·45–5·07)	<0·001
Age in months (Log)	0·71 (0·59–0·84)	<0·001	0·65 (0·52–0·82)	<0·001
Sex				
Female	1·23 (1·00–1·51)	0·05	1·46 (1·01–2·10)	0·05
Recruitment site variance	0·38 (0·13–1·11)		0·26 (0·07–0·98)	
AUC	0·71 (0·68–0·74)		0·73 (0·69–0·77)	

Abbreviations: HR; Hazard ratios, AUC; area under receiver operating characteristics.
Hazards ratio from mixed effect Weibull survival parametric model with site as a random effect controlling for sampling and LTFU weights.

Table S30. Characteristics associated with 30 day and post discharge mortality without sampling weights, but with LTFU weights only.

	30-day mortality		Post-discharge mortality	
	aHR (95% CI)[a]	P-value	aHR (95% CI)[a]	P-value
**Base model**				
Anthropometry at admission				
Not wasted	Reference		Reference	
Moderately wasted	2·15 (1·45–3·18)	<0·001	2·65 (1·52–4·61)	0·001
Severely wasted/Kwashiorkor	4·83 (2·98–7·81)	<0·001	4·39 (2·20–8·77)	<0·001
Age in months (log)	0·81 (0·68–0·97)	0·02	0·72 (0·54–0·97)	0·03
Sex: female	1·09 (0·84–1·40)	0·53	1·22 (0·81–1·83)	0·34
**Individual variables included in multivariable model**				
Discharged against medical advice	[b]		2·30 (1·38–3·83)	0·001
Admission days (log)	[b]		1·58 (0·97–2·57)	0·07
Change in undernutrition at discharge				
No change	[b]		Reference	
Improved	[b]		0·73 (0·57–0·95)	0·02
Worsened	[b]		0·67 (0·18–2·42)	0·54
HIV status				
Negative	Reference		Reference	
Untested	0·78 (0·40–1·51)	0·46	1·90 (0·89–4·06)	0·10
Exposed	1·58 (1·16–2·15)	0·004	1·94 (1·22–3·09)	0·005
Infected	2·16 (1·08–4·30)	0·03	1·85 (0·95–3·61)	0·07
**Domain scores**				
**Signs of illness severity at admission**				
Low	Reference		Reference	
Medium	1·32 (0·84–2·08)	0·23	1·24 (0·83–1·86)	0·30
High	3·70 (2·51–5·46)	<0·001	1·72 (0·97–3·05)	0·06
**Signs of illness severity at discharge**				
Low	[b]		Reference	
Medium	[b]		1·30 (0·91–1·87)	0·16
High	[b]		4·08 (1·81–9·18)	0·001
**Underlying medical conditions**				
Low	Reference		Reference	
Medium	2·11 (1·14–3·89)	0·02	0·89 (0·51–1·55)	0·69
High	1·71 (1·08–2·70)	0·02	1·43 (0·91–2·24)	0·13
**Child-level nutritional risk exposures**				
Low	Reference		Reference	
Medium	0·70 (0·43–1·15)	0·16	0·91 (0·43–1·92)	0·81
High	1·25 (0·87–1·80)	0·22	1·17 (0·77–1·79)	0·46
**Caregiver characteristics**				
Least adverse	Reference		Reference	
Moderately adverse	1·72 (1·19–2·49)	0·004	1·15 (0·65–2·03)	0·63
Most adverse	1·42 (0·98–2·07)	0·07	1·79 (1·03–3·11)	0·04
**Household-level exposures**				
Least adverse	Reference		Reference	
Moderately adverse	1·55 (1·00–2·42)	0·05	1·01 (0·79–1·28)	0·96
Most adverse	1·48 (1·05–2·09)	0·03	0·96 (0·59–1·56)	0·86
**Access to health care**				

Least adverse	Reference		Reference	
Moderately adverse	1·08 (0·74–1·57)	0·69	0·95 (0·60–1·49)	0·82
Most adverse	1·73 (1·08–2·79)	0·02	0·93 (0·53–1·61)	0·79
Recruitment site variance (95% CI)	0·16 (0·04–0·58)		0·06 (0·02–0·17)	
Bootstrapped AUC (95% CI)	0·82 (0·79–0·85)		0·82 (0·79–0·85)	

[a]aHR-adjusted Hazard ratios for all predictors in the multivariable model
[b]not included in the multivariable model, data available only at discharge.
HR from multilevel multivariable parametric (Weibull distribution) survival model with site as random effect.

Sensitivity analyses

Table S31. Characteristics associated with 30 day and post discharge mortality with low sampling weights (NW=82%, MW=8% and SWK=10%).

	30-days mortality		Post-discharge mortality	
	**aHR (95% CI)[a]**	**P-value**	**aHR (95% CI)[a]**	**P-value**
**Base model**				
Anthropometry at admission				
Not wasted	Reference		Reference	
Moderately wasted	1·95 (1·37–2·77)	0·001	2·53 (1·40–4·57)	0·002
Severely wasted/ Kwashiorkor	4·19 (2·47–7·16)	<0·001	4·32 (2·24–8·35)	<0·001
Age in months (log)	0·69 (0·53–0·91)	0·008	0·72 (0·51–1·01)	0·06
Sex: female	1·39 (0·88–2·18)	0·15	1·58 (0·92–2·70)	0·10
**Individual variables included in multivariable model**				
Discharged against medical advice	[b]		1·84 (1·07–3·19)	0·03
Admission days (log)	[b]		1·44 (0·91–2·26)	0·12
Change in undernutrition at discharge				
No change	[b]		Reference	
Improved	[b]		0·72 (0·56–0·94)	0·02
Worsened	[b]		1·08 (0·12–9·87)	0·95
HIV status				
Negative	Reference		Reference	
Untested	0·71 (0·25–2·06)	0·53	1·73 (0·52–5·74)	0·37
Exposed	1·46 (0·99–2·16)	0·06	1·59 (1·03–2·45)	0·04
Infected	2·56 (1·47–4·45)	0·001	1·41 (0·69–2·88)	0·35
**Domain scores**				
**Signs of illness severity at admission**				
Low	Reference		Reference	
Medium	1·17 (0·55–2·47)	0·69	1·56 (0·75–3·25)	0·24
High	3·92 (2·15–7·17)	<0·001	1·72 (0·78–3·79)	0·18
**Signs of illness severity at discharge**				
Low	[b]		Reference	
Medium	[b]		1·26 (0·89–1·79)	0·20
High	[b]		3·75 (1·22–11·5)	0·02
**Underlying medical conditions**				
Low	Reference		Reference	
Medium	3·11 (1·33–7·26)	0·009	0·94 (0·62–1·42)	0·77
High	2·92 (1·38–6·15)	0·005	1·37 (0·90–2·07)	0·14
**Child-level nutritional risk exposures**				
Low	Reference		Reference	
Medium	0·40 (0·24–0·69)	0·001	0·63 (0·32–1·25)	0·19
High	0·98 (0·57–1·68)	0·95	1·27 (0·75–2·15)	0·38
**Caregiver characteristic**				
Least adverse	Reference		Reference	
Moderately adverse	2·63 (1·49–4·63)	0·01	1·83 (1·11–3·02)	0·02
Most adverse	1·48 (0·74–2·97)	0·26	2·80 (1·39–5·63)	0·004
**Household-level exposures**				
Least adverse	Reference		Reference	
Moderately adverse	3·14 (1·92–5·12)	<0·001	1·08 (0·73–1·59)	0·70
Most adverse	3·12 (1·84–5·29)	<0·001	0·99 (0·63–1·56)	0·97
**Access to health care**				
Least adverse	Reference		Reference	
Moderately adverse	1·15 (0·69–1·93)	0·60	1·54 (0·88–2·71)	0·13

Most adverse	2·06 (1·41–3·01)	<0·001	1·61 (0·89–2·90)	0·11
Recruitment site variance (95% CI)	6·35e^{-34} (1·60e^{-41}–2·51e^{-26})		2·13e^{-31} (2·64e^{-38}–1·72e^{-24})	
Bootstrapped AUC (95% CI)	0·79 (0·76–0·82)		0·79 (0·76–0·82)	

[a]aHR-adjusted Hazard ratios for all predictors in the multivariable model
[b]not included in the multivariable model, data available only at discharge.
All model results were weighted using sampling and lost to follow up weights.
HR from multilevel multivariable parametric (Weibull distribution) survival model with site as random effect.

Table S32. Characteristics associated with 30 day and post discharge mortality with high sampling weights (NW=46%, MW=24% and SWK=30%).

	**30-days mortality**		**Post-discharge mortality**	
	**aHR (95% CI)[a]**	**P-value**	**aHR (95% CI)[a]**	**P-value**
Base model				
Anthropometry at admission				
Not wasted	Reference		Reference	
Moderately wasted	2·12 (1·44–3·12)	<0·001	2·61 (1·50–4·55)	0·001
Severely wasted/Kwash	4·76 (2·95–7·67)	<0·001	4·24 (2·19–8·21)	<0·001
Age in months (log)	0·78 (0·66–0·93)	0·06	0·71 (0·55–0·93)	0·01
Sex: female	1·10 (0·83–1·47)	0·50	1·26 (0·83–1·89)	0·28
**Individual variables included in multivariable model**				
Discharged against medical advice	[b]		2·26 (1·37–3·71)	0·001
Admission days (log)	[b]		1·63 (1·00–2·66)	0·05
Change in undernutrition at discharge				
No change	[b]		Reference	
Improved	[b]		0·76 (0·61–0·94)	0·01
Worsened	[b]		0·67 (0·17–2·69)	0·58
HIV status				
Negative	Reference		Reference	
Untested	0·81 (0·40–1·62)	0·55	2·01 (0·87–4·63)	0·10
Exposed	1·61 (1·22–2·14)	0·001	1·88 (1·18–3·02)	0·008
Infected	2·27 (1·12–4·57)	0·02	1·78 (0·89–3·57)	0·11
**Domain scores**				
**Signs of illness severity at admission**				
Low	Reference		Reference	
Medium	1·33 (0·82–2·16)	0·25	1·31 (0·87–1·98)	0·20
High	3·79 (2·52–5·71)	<0·001	1·73 (0·97–3·09)	0·06
**Signs of illness severity at discharge**				
Low	[b]		Reference	
Medium	[b]		1·28 (0·92–1·79)	0·14
High	[b]		4·06 (1·72–9·58)	0·001
**Underlying medical conditions**				
Low	Reference		Reference	
Medium	2·21 (1·15–4·22)	0·02	0·88 (0·54–1·42)	0·60
High	1·81 (1·14–2·89)	0·01	1·41 (0·95–2·10)	0·09
**Child-level nutritional risk exposures**				
Low	Reference		Reference	
Medium	0·63 (0·38–1·04)	0·07	0·89 (0·43–1·86)	0·76
High	1·19 (0·81–1·77)	0·37	1·15 (0·73–1·81)	0·55
**Caregiver characteristic**				
Least adverse	Reference		Reference	

Moderately adverse	1·86 (1·27–2·72)	0·002	1·21 (0·72–2·04)	0·48
Most adverse	1·48 (1·02–2·13)	0·04	1·90 (1·07–3·36)	0·03
**Household-level exposures**				
Least adverse	Reference		Reference	
Moderately adverse	1·73 (1·13–2·66)	0·01	1·06 (0·84–1·34)	0·61
Most adverse	1·67 (1·14–2·42)	0·008	1·03 (0·66–1·61)	0·88
**Access to health care**				
Least adverse	Reference		Reference	
Moderately adverse	1·06 (0·70–1·59)	0·79	0·98 (0·61–1·58)	0·95
Most adverse	1·72 (1·07–2·76)	0·03	1·00 (0·57–1·74)	0·96
Recruitment site variance (95% CI)	0·12 (0·02–0·63)		0·05 (0·01–0·23)	
Bootstrapped AUC (95% CI)	0·81 (0·79–0·84)		0·82 (0·79–0·85)	

[a]aHR-adjusted Hazard ratios for all predictors in the multivariable model
[b]not included in the multivariable model, data available only at discharge.
All model results were weighted using sampling and lost to follow up weights.
HR from multilevel multivariable parametric (Weibull distribution) survival model with site as random effect.

Table S33. Characteristics associated with 30 day and post discharge mortality with anthropometric strata.

	30-days mortality		Post-discharge mortality	
	**aHR (95% CI)[a]**	**P-value**	**aHR (95% CI)[a]**	**P-value**
**Base model**				
Anthropometry at admission				
Not wasted	Reference		Reference	
Moderately wasted	2·04 (1·41–2·95)	<0·001	2·61 (1·53–4·42)	<0·001
Severely wasted	4·18 (2·44–7·14)	<0·001	4·48 (2·19–9·18)	<0·001
Kwashiorkor	6·19 (4·53–8·45)	<0·001	3·98 (1·71–9·28)	0·001
Age in months (log)	0·73 (0·61–0·86)	<0·001	0·72 (0·47–1·10)	0·13
Sex: female	1·29 (0·98–1·70)	0·07	1·33 (0·90–1·97)	0·16
**Individual variables included in multivariable model**				
Discharged against medical advice	[b]		2·16 (1·45–3·23)	<0·001
Admission days (log)	[b]		1·47 (0·92–2·33)	0·10
Change in undernutrition at discharge				
No change	[b]		Reference	
Improved	[b]		0·68 (0·48–0·95)	0·03
Worsened	[b]		0·80 (0·14–4·64)	0·80
HIV status				
Negative	Reference		Reference	
Untested	0·70 (0·32–1·52)	0·37	1·66 (0·67–4·15)	0·28
Exposed	1·64 (1·12–2·42)	0·01	1·85 (1·14–3·00)	0·01
Infected	2·35 (1·46–3·79)	<0·001	1·76 (1·00–3·10)	0·05
**Domain scores**				
**Signs of illness severity at admission**				
Low	Reference		Reference	
Medium	1·34 (0·76–2·35)	0·31	1·32 (0·87–1·99)	0·20
High	4·07 (2·55–6·50)	<0·001	1·87 (1·11–3·15)	0·02
**Signs of illness severity at discharge**				
Low	[b]		Reference	
Medium	[b]		1·27 (0·88–1·85)	0·21
High	[b]		4·57 (1·94–10·7)	0·001
**Underlying medical conditions**				
Low	Reference		Reference	
Medium	2·60 (1·49–4·55)	0·001	1·01 (0·64–1·60)	0·97
High	2·05 (1·34–3·12)	0·001	1·67 (1·22–2·30)	0·001

**Child-level nutritional risk exposures**				
Low	Reference		Reference	
Medium	0·64 (0·41–1·01)	0·05	0·81 (0·38–1·73)	0·59
High	1·10 (0·85–1·43)	0·46	1·14 (0·69–1·88)	0·62
**Caregiver characteristic**				
Least adverse	Reference		Reference	
Moderately adverse	1·96 (1·24–3·10)	0·004	1·26 (0·77–2·07)	0·36
Most adverse	1·69 (1·09–2·61)	0·02	2·21 (1·45–3·38)	<0·001
**Household-level exposures**				
Least adverse	Reference		Reference	
Moderately adverse	1·87 (1·34–2·60)	<0·001	0·94 (0·64–1·38)	0·77
Most adverse	2·12 (1·44–3·10)	<0·001	0·92 (0·66–1·27)	0·60
**Access to health care**				
Least adverse	Reference		Reference	
Moderately adverse	1·14 (0·74–1·78)	0·55	1·07 (0·65–1·79)	0·78
Most adverse	1·69 (1·01–2·85)	0·05	1·00 (0·57–1·75)	0·98
Recruitment site variance (95% CI)	0·02 (0·001–0·58)		0·02 (0·002–0·23)	
Bootstrapped AUC (95% CI)	0·81 (0·77–0·84)		0·81 (0·78–0·85)	

[a]aHR-adjusted Hazard ratios for all predictors in the multivariable model
[b]not included in the multivariable model, data available only at discharge.
All model results were weighted using sampling and lost to follow up weights.
HR from multilevel multivariable parametric (Weibull distribution) survival model with site as random effect.

Table S34: Characteristics associated with early (within 30 days from discharge) and late (after 30 days from discharge) post-discharge mortality.

	**Early post-discharge deaths**		**Late post-discharge deaths**	
	aHR (95% CI)[a]	P-value	aHR (95% CI)[a]	P-value
**Base model**				
Anthropometric strata at admission				
Not wasted	Reference		Reference	
Moderately wasted	3·07 (0·84–11·3)	0·09	2·43 (1·23–4·80)	0·01
Severely wasted or Kwashiorkor	6·17 (2·27–16·8)	<0·001	3·72 (1·78–7·78)	<0·001
Age months (log)	0·60 (0·45–0·82)	0·001	0·81 (0·56–1·17)	0·26
Sex female	1·30 (0·88–1·91)	0·19	1·34 (0·78–2·32)	0·29
Discharged against medical advice	3·01 (1·63–5·58)	<0·001	1·68 (0·93–3·04)	0·09
Admission duration days (log)	1·32 (0·82–2·15)	0·26	1·68 (0·93–3·04)	0·09
Change in anthropometry at discharge				
No change	Reference		Reference	
Improved	0·64 (0·35–1·16)	0·14	0·78 (0·57–1·05)	0·10
Worsened	2·36 (0·17–32·4)	0·52	0·26 (0·03–2·24)	0·22
**Exposure Domains**				
**Signs of illness severity at admission**				
Low	Reference		Reference	
Medium	1·56 (0·69–3·52)	0·29	1·22 (0·56–2·67)	0·62
High	1·47 (0·79–2·70)	0·22	1·89 (0·89–4·03)	0·09
**Signs of illness severity at discharge**				
Low	Reference		Reference	
Medium	1·55 (1·08–2·23)	0·02	1·18 (0·71–1·96)	0·51
High	4·37 (1·28–14·9)	0·02	3·87 (1·62–9·25)	0·002
**HIV status**				
Negative	Reference		Reference	

Untested	0·48 (0·07–3·38)	0·46	3·00 (1·08–8·30)	0·03
Exposed	1·48 (0·91–2·41)	0·11	1·92 (1·10–3·35)	0·02
Infected	1·41 (0·80–2·48)	0·24	2·10 (0·87–5·03)	0·09
**Underlying medical conditions**				
Low	Reference		Reference	
Medium	1·47 (0·60–3·56)	0·40	0·69 (0·44–1·07)	0·09
High	1·68 (0·88–3·21)	0·12	1·26 (0·68–2·33)	0·45
**Child-level nutritional risk exposures**				
Low	Reference		Reference	
Medium	1·04 (0·28–3·89)	0·95	0·76 (0·34–1·67)	0·49
High	1·68 (0·81–3·48)	0·17	0·98 (0·63–1·52)	0·93
**Caregiver characteristics**				
Least adverse	Reference		Reference	
Moderately adverse	1·16 (0·62–2·16)	0·64	1·56 (0·74–3·28)	0·24
Most adverse	1·22 (0·50–2·99)	0·67	2·88 (1·39–5·99)	0·005
**Household-level exposures**				
Least adverse	Reference		Reference	
Moderately adverse	0·80 (0·42–1·50)	0·49	1·22 (0·98–1·51)	0·07
Most adverse	0·61 (0·29–1·28)	0·19	1·36 (0·89–2·07)	0·15
**Access to health care**				
Least adverse	Reference		Reference	
Moderately adverse	1·41 (0·73–2·71)	0·31	0·95 (0·54–1·68)	0·87
Most adverse	1·57 (0·74–3·35)	0·24	0·92 (0·53–1·56)	0·72
Site variance (95% CI)	1·45^{e-32} (1·39^{e-33}–1·51^{e-31})		0·01 (1·77^{e06}–56·3)	
Bootstrapped AUC (95% CI)	0·83 (0·78−0·88)		0·81 (0·77−0·85)	

[a]aHR-adjusted Hazard ratios for all predictors in the multivariable model
All model results were weighted using sampling and lost to follow up weights.
HR from multilevel multivariable parametric (Weibull distribution) survival model with site as random effect.
Early deaths are post-discharge deaths in first month after index discharge (n=62).
Late deaths are post-discharge deaths after the first month following index discharge (n=106).

Mortality across quintiles of regression model predictions

Table S35. 30-day mortality across quintiles of predicted mortality from the final 30-day regression model including sample weights and LTFU.

Quintiles	Admissions	Died	% Died	Median (IQR) days after admission to death
1st	621	5	0·8	8 (3–14)
2nd	620	12	1·9	10 (5–18)
3rd	620	25	4·0	8 (3–10)
4th	620	46	7·4	10 (3–20)
5th	620	146	24	2 (1–9)

Table S36. Post-discharge mortality across quintiles of predicted mortality from the final post-discharge regression model including sample weights and LTFU.

Quintiles	Discharges	Died	% Died	Median (IQR) days after discharge to death
1st	575	5	0·9	33 (9–42)

2nd	575	7	1·2	102 (78–154)
3rd	575	17	3·0	44 (12–83)
4th	575	38	6·6	46 (18–90)
5th	574	101	18	45 (17–107)

Table S37. Discharge against medical advice across quintiles of predicted mortality from the final post-discharge regression model including sample weights and LTFU.

Quintiles	Discharges	Discharged against advice	Died among children discharged against advice	% Died among children discharged against advice
1st	575	6	0	0
2nd	575	16	0	0
3rd	575	30	1	3·3
4th	575	31	2	6·4
5th	574	74	19	26

Table S38. Structural Equation Model full estimation results of the relationships between pre-existing characteristics, characteristics at admission, and mortality within 30 days

OUTCOME:	Mortality within 30 days			Nutritional strata at admission			Signs of illness severity at admission			HIV status			Underlying medical conditions			Child-level nutritional risk exposures			Caregiver characteristics			Access to healthcare		
				Characteristics at admission						Pre-existing characteristics														
	aHR*	95% CI	*P*	aOR*	95% CI	*P*	aOR*	95% CI	*P*	aOR*	95% CI	*P*	aOR*	95% CI	*P*	aOR*	95% CI	*P*	aOR*	95% CI	*P*	aOR*	95% CI	*P*
Base model																								
Nutritional strata at admission** →																								
NW	Reference						Reference			¶			¶			¶			¶			¶		
MW	2·07	1·42-3·04	<·001				1·04	0·77-1·39	0·81	¶			¶			¶			¶			¶		
SWK	4·56	2·63-7·90	<·001				0·63	0·45-0·88	0·01	¶			¶			¶			¶			¶		
Signs of illness severity at admission →																								
Low	Reference			¶						¶			¶			¶			¶			¶		
Medium	1·25	0·71-2·22	0·44	¶						¶			¶			¶			¶			¶		
High	3·67	2·35-5·75	<·001	¶						¶			¶			¶			¶			¶		
Age in months (log) →	0·75	0·62-0·90	0·002	0·53	0·41-0·69	<·001	0·66	0·61-0·72	<·001	0·81	0·59-1·12	0·2	1·29	1·01-1·65	0·04	1·34	1·14-1·57	<·001	1·17	1·00-1·37	0·04	1·03	0·93-1·14	0·57
Sex: female →	1·21	0·85-1·73	0·29	1·64	1·36-1·97	<·001	1·04	0·91-1·20	0·55	0·94	0·82-1·08	0·4	0·71	0·61-0·84	<·001	1·29	1·07-1·56	0·008	0·97	0·81-1·17	0·78	1·12	0·98-1·28	0·11
HIV status →																								
Negative	Reference			Reference			Reference						Reference			Reference			¶			¶		
Untested	0·68	0·31-1·48	0·33	0·47	0·25-0·90	0·02	1·26	0·60-2·66	0·54				1·34	0·65-2·75	0·43	0·84	0·51-1·41	0·52	¶			¶		
Exposed	1·51	1·11-2·06	0·009	1·23	0·87-1·75	0·24	0·96	0·60-1·53	0·87				1·66	1·06-2·58	0·03	1·14	0·73-1·79	0·56	¶			¶		
Infected	2·27	1·25-4·12	0·007	2·04	1·03-4·05	0·04	1·16	0·65-2·07	0·63				3·2	2·09-4·89	<·001	2·69	1·41-5·11	0·003	¶			¶		
Underlying medical conditions →																								
Low	Reference			Reference			Reference			¶						¶			¶			¶		
Medium	2·48	1·21-5·08	0·01	2·04	1·58-2·63	<·001	1·1	0·85-1·43	0·47	¶						¶			¶			¶		
High	2·06	1·17-3·60	0·01	10·26	6·89-15·28	<·001	1·23	0·86-1·78	0·26	¶						¶			¶			¶		
Child-level nutritional risk exposures →																								
Low	Reference			Reference			Reference			¶			Reference						¶			¶		
Medium	0·55	0·34-0·90	0·02	3·24	2·01-5·22	<·001	0·84	0·58-1·24	0·39	¶			1·47	0·92-2·33	0·1				¶			¶		
High	1·13	0·79-1·60	0·5	8·82	5·09-15·28	<·001	0·86	0·54-1·39	0·54	¶			2·49	1·89-3·27	<·001				¶			¶		
Caregiver characteristic →																								
Least adverse	Reference			Reference			Reference			Reference			¶			Reference						¶		
Moderately adverse	2·15	1·27-3·65	0·004	0·97	0·61-1·53	0·89	1·38	0·82-2·33	0·22	0·93	0·61-1·40	0·72	¶			1·65	0·95-2·88	0·08				¶		
Most adverse	1·54	0·87-2·75	0·14	1·04	0·65-1·68	0·86	1·19	0·71-1·99	0·51	1·71	1·26-2·31	0·001	¶			1·32	0·85-2·03	0·22				¶		
Household-level exposures →																								
Least adverse	Reference			Reference			Reference			Reference			Reference			Reference			Reference			Reference		
Moderately adverse	2·33	1·51-3·58	<·001	1·23	0·90-1·67	0·19	0·92	0·66-1·28	0·61	1·85	0·90-3·82	0·1	0·89	0·72-1·11	0·31	1·15	0·92-1·44	0·21	1·79	1·12-2·87	0·02	1·5	0·86-2·63	0·15
Most adverse	2·34	1·45-3·79	0·001	1·09	0·71-1·68	0·69	0·79	0·53-1·19	0·26	1·76	0·62-5·02	0·29	0·71	0·54-0·93	0·01	1·38	0·95-2·00	0·1	2·09	0·86-5·06	0·11	2·44	1·34-4·47	0·004
Access to health care →																								
Least adverse	Reference			Reference			Reference			Reference			¶			¶			¶					
Moderately adverse	1·16	0·73-1·85	0·52	1·26	0·99-1·59	0·06	0·98	0·76-1·26	0·85	1·24	0·89-1·73	0·21	¶			¶			¶					
Most adverse	2·13	1·32-3·45	0·002	1·33	0·98-1·80	0·07	1·01	0·78-1·31	0·95	1·5	0·99-2·26	0·054	¶			¶			¶					
Site variance	0·06	0·0003-11·01																						
Bootstrapped AUC	0·81	0·78-0·84																						

Notes: *Adjusted hazard ratios (aHR) are given for the primary outcome of mortality (time-to-event, survival, Weibull) and adjusted odds ratios (aOR) are given for intermediate outcomes (ordinal logistic regression for three outcome levels). **NW = Not Wasted, MW = moderate wasting, SWK = severe wasting or Kwashiorkor. ¶Variable was not included as predictor for the respective outcome. Household-level risk exposures, age, and sex were not an outcome in this Structural Equation Model. Each outcome was modelled with a random intercept at the site level. Model results were weighted using sampling and lost to follow up weights.

Table S39· Structural Equation Model results for the relationships between pre-existing characteristics, characteristics at admission, and post-discharge mortality

				Characteristics at discharge												Characteristics at admission						Pre-existing characteristics														
**OUTCOME:**	**Post-discharge mortality**			**Change in nutritional strata at discharge**			**Signs of illness severity at discharge**			**Admission duration**			**Discharged against medical advice**			**Nutritional strata at admission**			**Signs of illness severity at admission**			**HIV status**			**Underlying medical conditions**			**Child-level nutritional risk exposures**			**Caregiver characteristics**			**Access to healthcare**		
	aHR*	95% CI	*P*	aOR*	95% CI	*P*	aOR*	95% CI	*P*	aOR*	95% CI	*P*	aOR*	95% CI	*P*	aOR*	95% CI	*P*	aOR*	95% CI	*P*	aOR*	95% CI	*P*	aOR*	95% CI	*P*	aOR*	95% CI	*P*	aOR*	95% CI	*P*	aOR*	95% CI	*P*
Base model																																				
Change in nutritional strata at discharge →																																				
Improved	0·7	0·56-0·89	0·003				¶			¶			¶			¶			¶			¶			¶			¶			¶			¶		
No change	Reference						¶			¶			¶			¶			¶			¶			¶			¶			¶			¶		
Worsened	0·85	0·16-4·44	0·85				¶			¶			¶			¶			¶			¶			¶			¶			¶			¶		
Signs of illness severity at discharge →																																				
Low	Reference			¶						¶			¶			¶			¶			¶			¶			¶			¶			¶		
Medium	1·24	0·89-1·74	0·2	¶						¶			¶			¶			¶			¶			¶			¶			¶			¶		
High	3·68	1·56-8·67	0·003	¶						¶			¶			¶			¶			¶			¶			¶			¶			¶		
Admission duration →																																				
Days (log)	1·44	0·90-2·30	0·13	1·1	0·77-1·56	0·6	0·85	0·64-1·12	0·25				¶			¶			¶			¶			¶			¶			¶			¶		
Discharged against medical advice →																																				
Yes	2·05	1·25-3·35	0·004	1·93	1·11-3·35	0·02	5·63	1·57-20·21	0·01	0·72	0·63-0·83	<·001				¶			¶			¶			¶			¶			¶			¶		
Nutritional strata at admission** →																																				
NW	Reference			Reference			Reference			Reference			Reference						Reference			¶			¶			¶			¶			¶		
MW	2·65	1·49-4·70	0·001	0·07	0·02-0·23	<·001	0·88	0·69-1·12	0·3	1·22	1·10-1·34	<·001	0·92	0·50-1·70	0·79				1·02	0·77-1·36	0·87	¶			¶			¶			¶			¶		
SWK	4·45	2·21-8·96	<·001	0·01	0·01-0·02	<·001	1·13	0·77-1·64	0·54	1·9	1·47-2·46	<·001	1·63	0·68-3·93	0·28				0·6	0·43-0·84	0·002	¶			¶			¶			¶			¶		
Signs of illness severity at admission →																																				
Low	Reference			Reference			Reference			Reference			Reference			¶						¶			¶			¶			¶			¶		
Medium	1·32	0·83-2·10	0·24	1·11	0·72-1·19	0·55	1·42	1·02-1·99	0·04	1·01	0·94-1·08	0·85	1·34	0·95-1·90	0·1	¶						¶			¶			¶			¶			¶		
High	1·7	0·97-3·00	0·07	0·47	0·86-1·49	0·37	2·81	1·79-4·43	<·001	1·13	1·02-1·24	0·02	1·46	0·99-2·15	0·06	¶						¶			¶			¶			¶			¶		
Age in months (log) →	0·72	0·56-0·93	0·01	0·47	0·33-0·66	<·001	1·1	0·98-1·23	0·09	0·97	0·91-1·03	0·32	0·94	0·75-1·17	0·57	0·52	0·39-0·68	<·001	0·7	0·64-0·77	<·001	0·85	0·59-1·22	0·37	1·28	0·96-1·70	0·09	1·25	1·05-1·49	0·01	1·16	0·99-1·37	0·06	1·01	0·93-1·10	0·75
Sex female →	1·33	0·86-2·05	0·19	1·11	0·80-1·53	0·54	1	0·81-1·23	1	0·95	0·94-1·08	0·85	1·21	0·84-1·74	0·31	1·66	1·35-2·04	<·001	1·03	0·89-1·19	0·69	0·99	0·88-1·13	0·92	0·71	0·63-0·81	<·001	1·32	1·11-1·57	0·001	0·95	0·79-1·13	0·55	1·12	0·99-1·28	0·08
HIV status →																																				
Negative	Reference			Reference			Reference			Reference			Reference			Reference			Reference						Reference			Reference			¶			¶		
Untested	1·75	0·73-4·20	0·21	2·32	1·00-5·37	0·05	1·37	0·74-2·55	0·32	0·93	0·74-1·17	0·54	0·76	0·43-1·37	0·36	0·52	0·29-0·94	0·03	1·33	0·56-3·14	0·52				1·39	0·69-2·78	0·36	0·89	0·57-1·38	0·6	¶			¶		
Exposed	1·63	1·09-2·44	0·02	0·93	0·54-1·61	0·8	0·63	0·45-0·88	0·01	0·95	0·88-1·03	0·19	0·63	0·20-1·97	0·43	1·09	0·71-1·66	0·7	0·91	0·59-1·40	0·67				1·53	0·97-2·41	0·07	1·15	0·70-1·89	0·57	¶			¶		
Infected	1·63	0·84-3·17	0·15	1·4	0·81-2·39	0·23	0·92	0·56-1·50	0·73	1·15	1·03-1·28	0·01	1·2	0·41-3·47	0·74	1·76	0·78-3·95	0·17	1·06	0·54-2·06	0·87				3·06	2·03-4·61	<·001	2·73	1·39-5·35	0·004	¶			¶		
Underlying medical conditions →																																				
Low	Reference			Reference			Reference			Reference			Reference			Reference			Reference			¶						¶			¶			¶		
Medium	0·89	0·64-1·25	0·51	1·35	1·05-1·74	0·02	1·09	0·81-1·45	0·58	1·04	0·97-1·13	0·29	1·01	0·86-1·19	0·91	2·12	1·62-2·77	<·001	1·02	0·76-1·37	0·9	¶						¶			¶			¶		
High	1·37	1·04-1·82	0·03	2·88	2·12-3·90	<·001	1·22	0·68-2·19	0·5	1	0·93-1·07	0·96	1·01	0·55-1·86	0·97	11	7·73-15·64	<·001	1·14	0·77-1·67	0·52	¶						¶			¶			¶		
Child-level nutritional risk exposures →																																				
Low	Reference			Reference			Reference			Reference			Reference			Reference			Reference			¶			Reference						¶			¶		
Medium	0·84	0·42-1·67	0·62	2·17	1·42-3·34	<·001	0·94	0·65-1·36	0·74	0·97	0·86-1·10	0·64	1·31	0·92-1·87	0·14	3·11	1·92-5·05	<·001	0·9	0·63-1·27	0·54	¶			1·64	1·04-2·58	0·03				¶			¶		
High	1·22	0·77-1·95	0·4	1·73	1·23-2·42	0·001	1·01	0·74-1·38	0·95	1·17	1·03-1·33	0·02	1·63	0·86-3·08	0·13	8·55	5·16-14·17	<·001	0·85	0·52-1·38	0·51	¶			2·57	1·97-3·36	<·001				¶			¶		
Caregiver characteristic →																																				
Least adverse	Reference			Reference			Reference			Reference			Reference			Reference			Reference			Reference			¶			Reference						¶		
Moderately adverse	1·36	0·92-2·02	0·13	1·14	0·66-1·98	0·65	0·91	0·67-1·25	0·56	1·07	0·91-1·17	0·64	0·85	0·51-1·41	0·53	1	0·61-1·61	0·99	1·24	0·67-1·22	0·53	0·69	0·35-1·35	0·28	¶			1·7	0·95 to 3·03	0·07				¶		
Most adverse	2	1·16-3·46	0·01	1·03	0·66-1·62	0·89	0·82	0·63-1·08	0·17	1·07	0·79-1·05	0·18	0·72	0·49-1·05	0·09	0·99	0·60-1·66	0·98	1·18	0·53-1·19	0·27	1·59	1·15-2·20	0·005	¶			1·34	0·88 to 2·05	0·18				¶		
Household-level exposures →																																				
Least adverse	Reference			Reference			Reference			Reference			Reference			Reference			Reference			Reference			Reference			Reference			Reference			Reference		
Moderately adverse	1·06	0·71-1·60	0·77	0·85	0·63-1·13	0·26	1·06	0·76-1·49	0·72	1·03	0·91-1·17	0·64	0·47	0·36-0·62	<·001	1·26	0·93-1·71	0·14	0·91	0·67-1·22	0·53	2	0·85-4·71	0·11	0·87	0·71-1·07	0·19	1·13	0·88-1·46	0·35	1·88	1·17-3·02	0·009	1·28	0·66-2·48	0·47
Most adverse	1·03	0·72-1·47	0·88	0·7	0·43-1·14	0·15	1	0·54-1·86	0·99	0·91	0·79-1·05	0·18	0·38	0·15-0·99	0·047	1·01	0·68-1·52	0·95	0·79	0·53-1·19	0·27	2·33	0·57-9·57	0·24	0·68	0·55-0·84	<·001	1·32	0·90-1·94	0·16	2·21	0·82-5·95	0·12	2·03	0·94-4·36	0·07
Access to health care →																																				
Least adverse	Reference			Reference			Reference			Reference			Reference			Reference			Reference			Reference			¶			¶			¶					
Moderately adverse	1·14	0·68-1·92	0·62	0·9	0·57-1·43	0·66	1·27	1·00-1·62	0·05	1·08	1·03-1·14	0·001	1·04	0·66-1·64	0·87	1·25	1·00-1·57	0·05	0·97	0·78-1·21	0·78	1·07	0·76-1·51	0·69	¶			¶			¶					
Most adverse	1·27	0·66-2·42	0·47	1·1	0·75-1·61	0·63	1·08	0·65-1·79	0·76	1·08	1·00-1·17	0·06	1·23	0·45-3·40	0·69	1·39	0·98-1·97	0·06	0·99	0·78-1·25	0·9	1·22	0·69-2·16	0·49	¶			¶			¶					
Site variance	0·02	0·004-1·51																																		
Bootstrapped AUC	0·81	0·77-0·84																																		

Notes: *Adjusted hazard ratios (aHR) are given for the primary outcome of mortality (time-to-event, survival, Weibull) and adjusted odds ratios (aOR) are given for intermediate outcomes (ordinal logistic regression for three outcome levels). **NW = Not Wasted, MW = moderate wasting, SWK = severe wasting or Kwashiorkor. ¶Variable was not included as predictor for the respective outcome. Household-level risk exposures, age, and sex were not an outcome in this Structural Equation Model. Each outcome was modelled with a random intercept at the site level. Model results were weighted using sampling and lost to follow up weights. Domains that are predictors are shown in rows and domains that are are outcomes are shown in columns.

Supplementary Figures

Figure S1. CHAIN causal framework adapted from UNICEF.

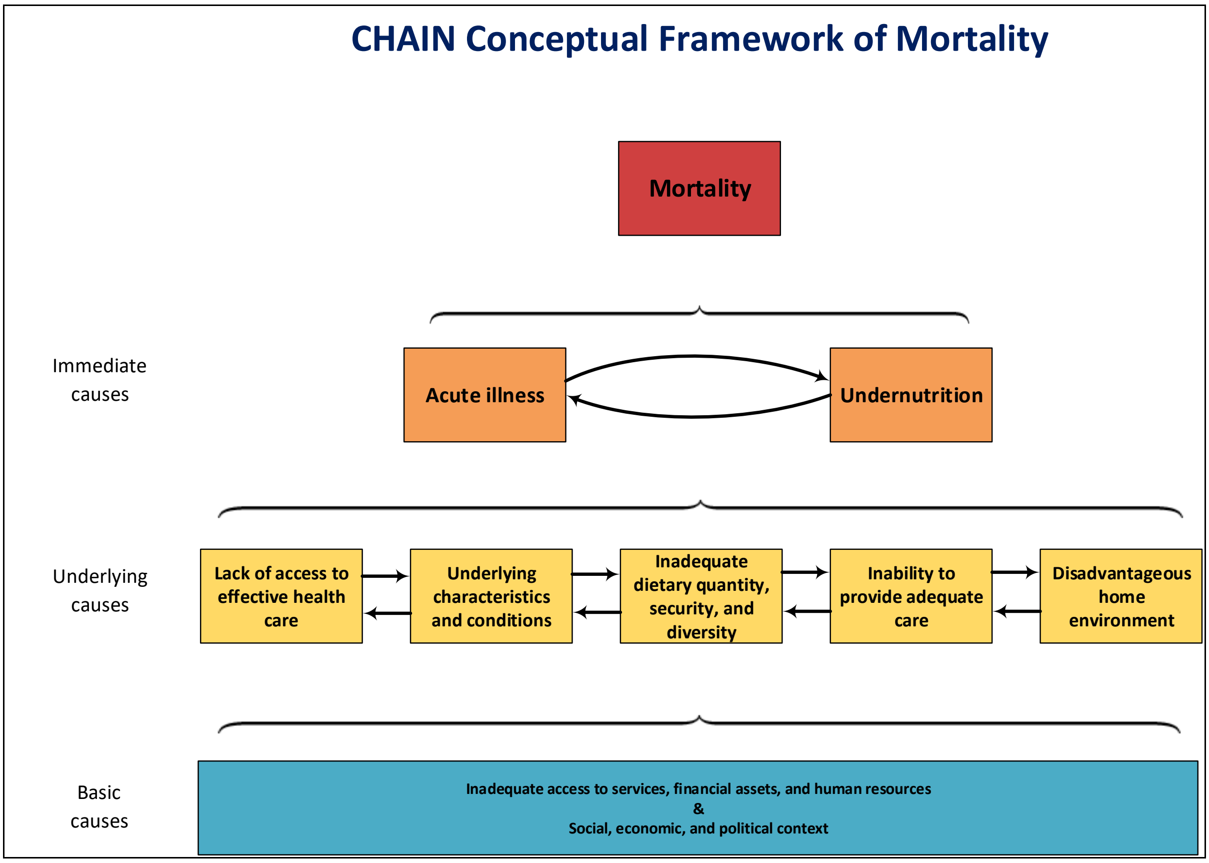

Adapted from: UNICEF (2013). Improving Child Nutrition - The achievable imperative for global progress. UNICEF.

Figure S2. Study Flow

LTFU: Lost to follow-up.

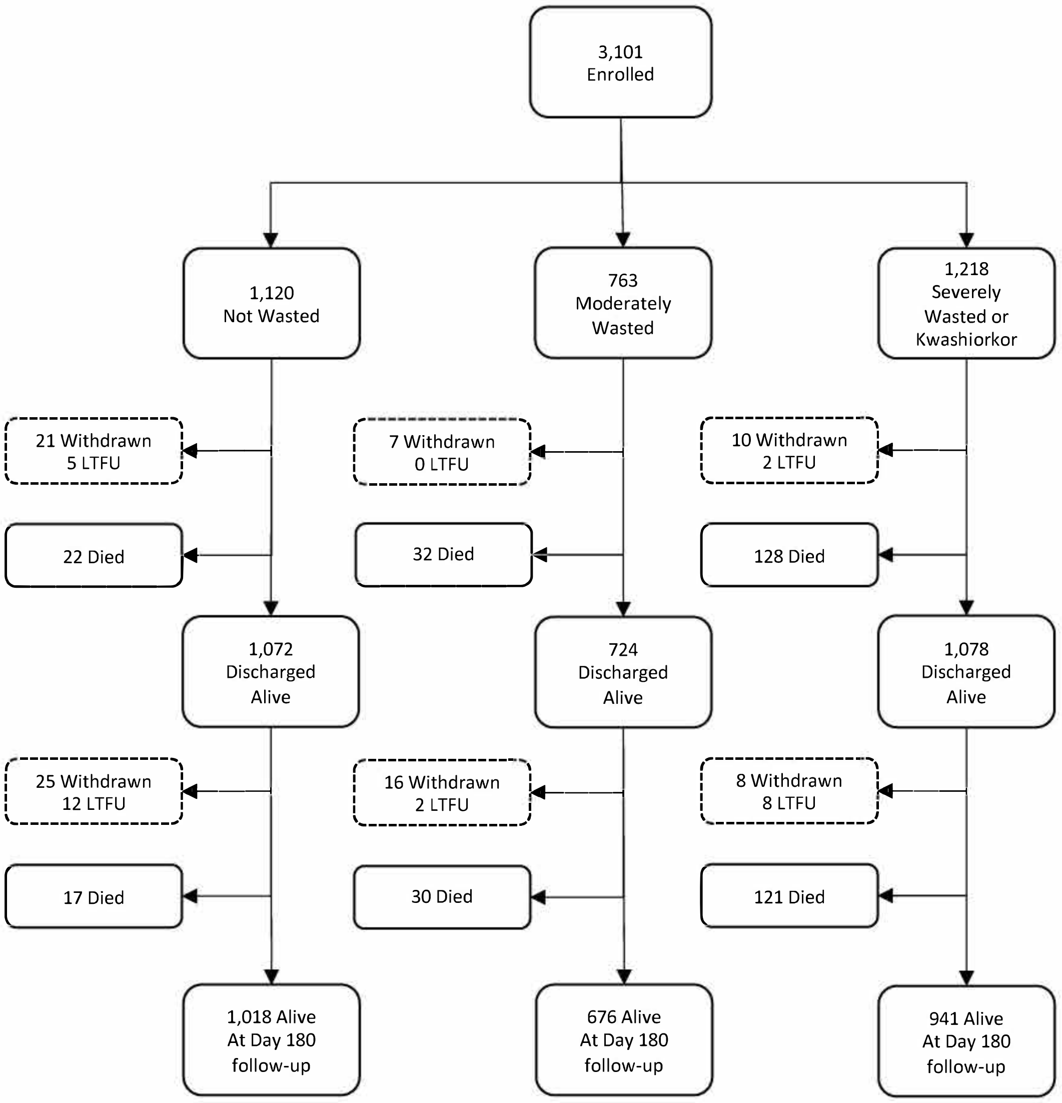

Figure S3. Cox-Snell residual plots.

A-Exponential, B-Weibull, C-Lognormal and D-Log-logistic distributions

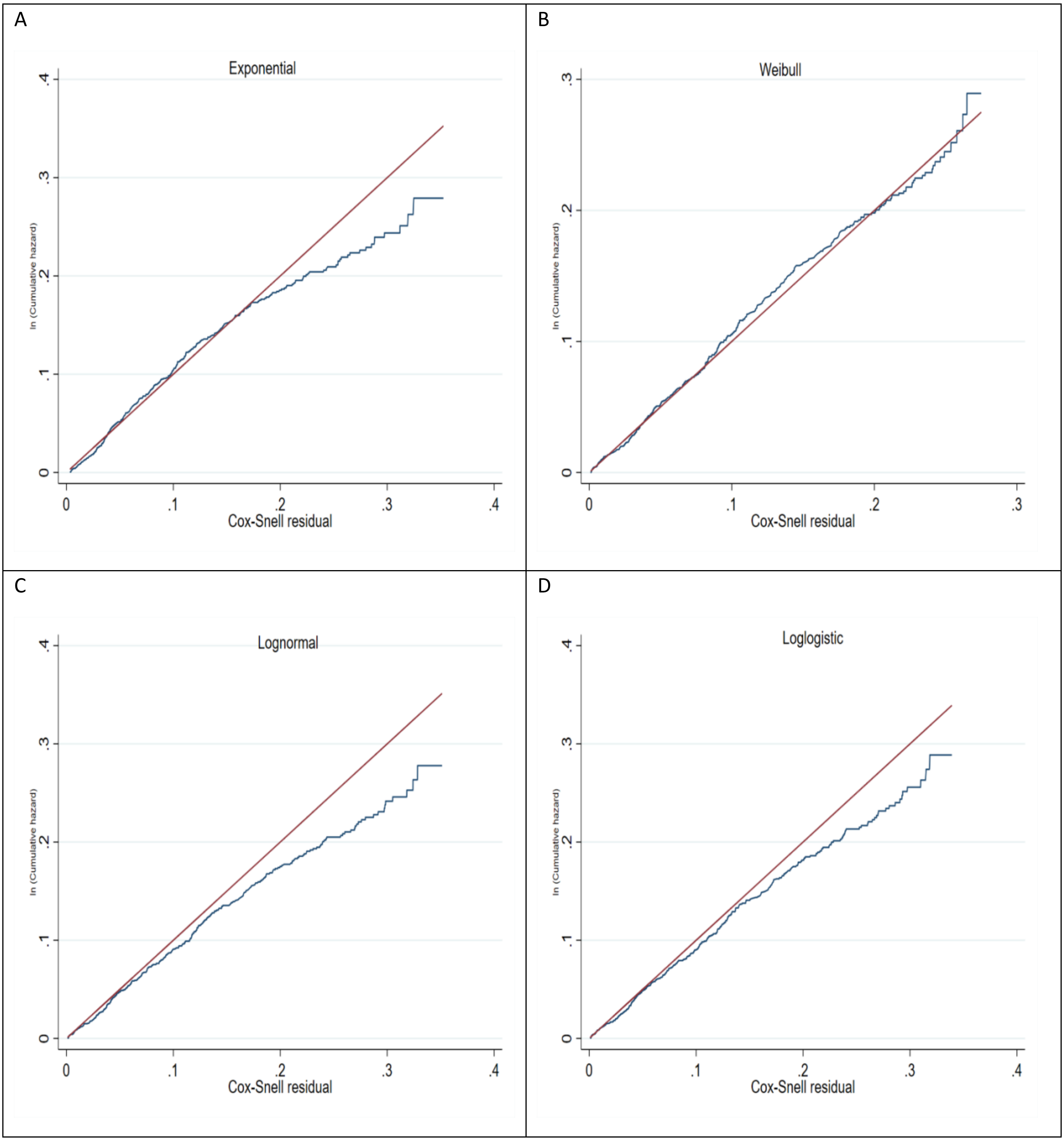

Figure S4. Cumulative hazard curves for LTFU and withdrawals combined.

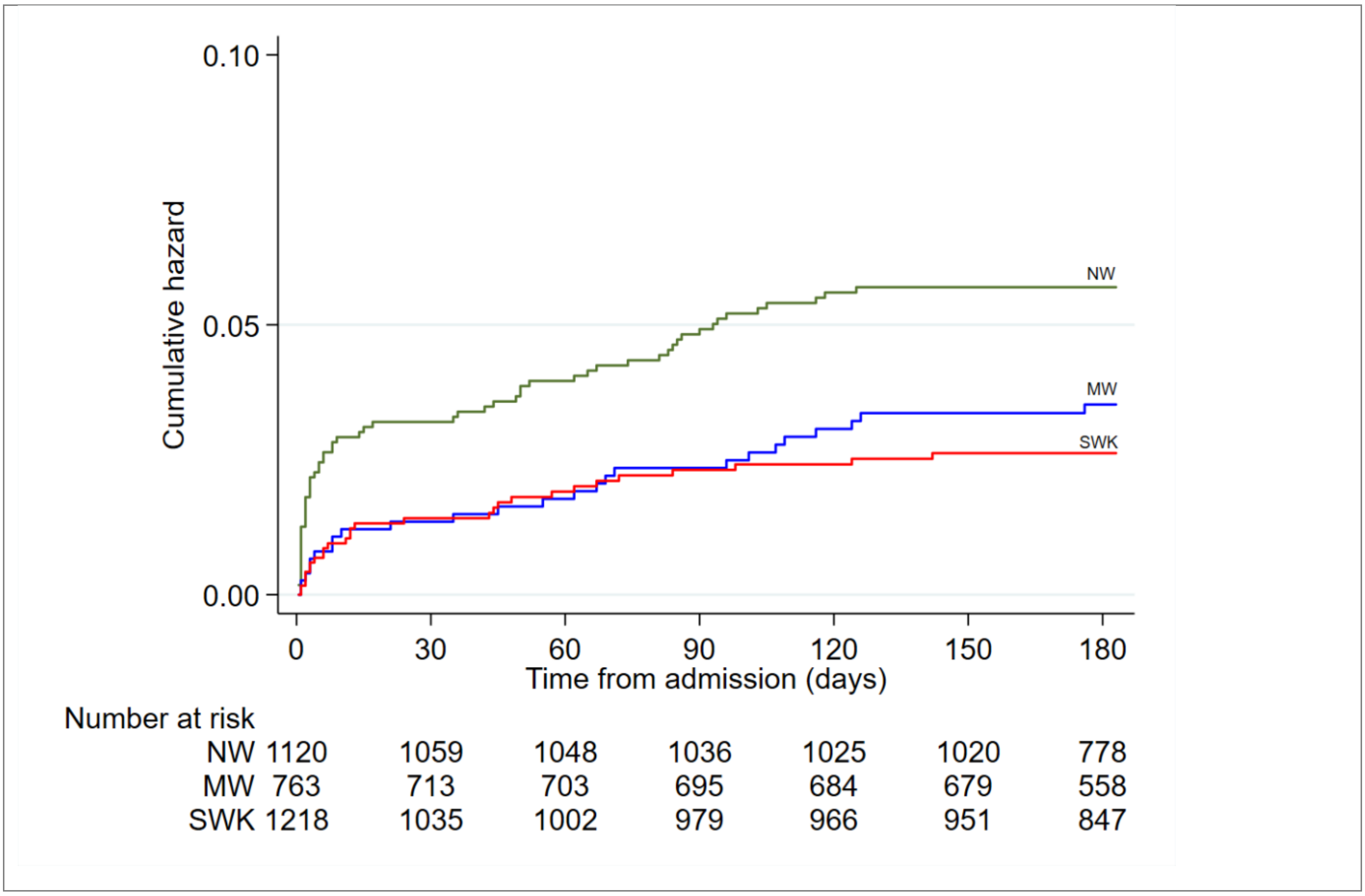

NW: Not wasted, MW: Moderately wasted, SWK: Severely wasted/Kwashiorkor.

Figure S5. Length of inpatient stay.

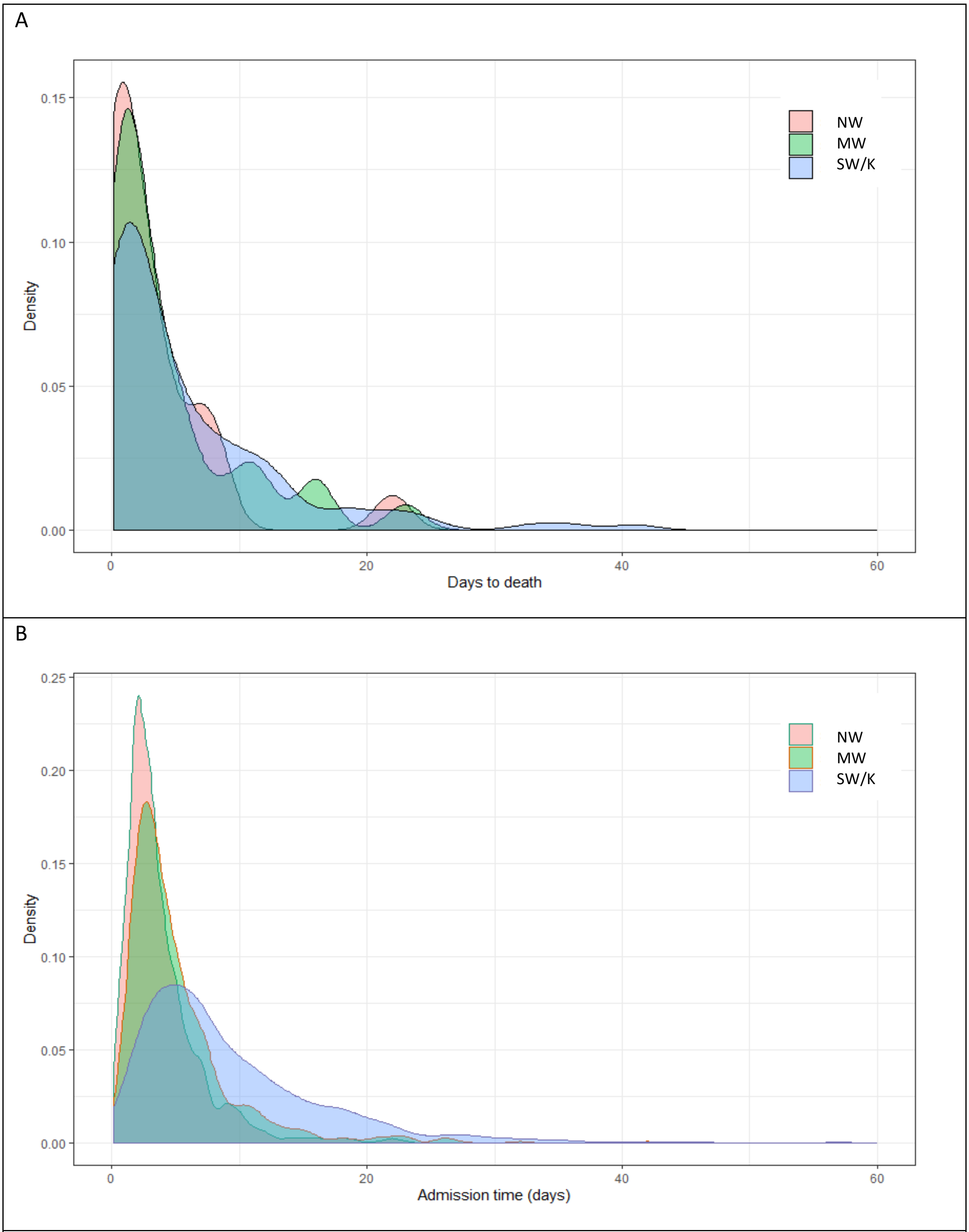

A) Days to index admission deaths and B) Length of stay among index admission survivors.

NW: Not wasted, MW: Moderately wasted, SWK: Severely wasted/Kwashiorkor.

Figure S6. Proportions of index admission and post-discharge deaths by anthropometric strata.

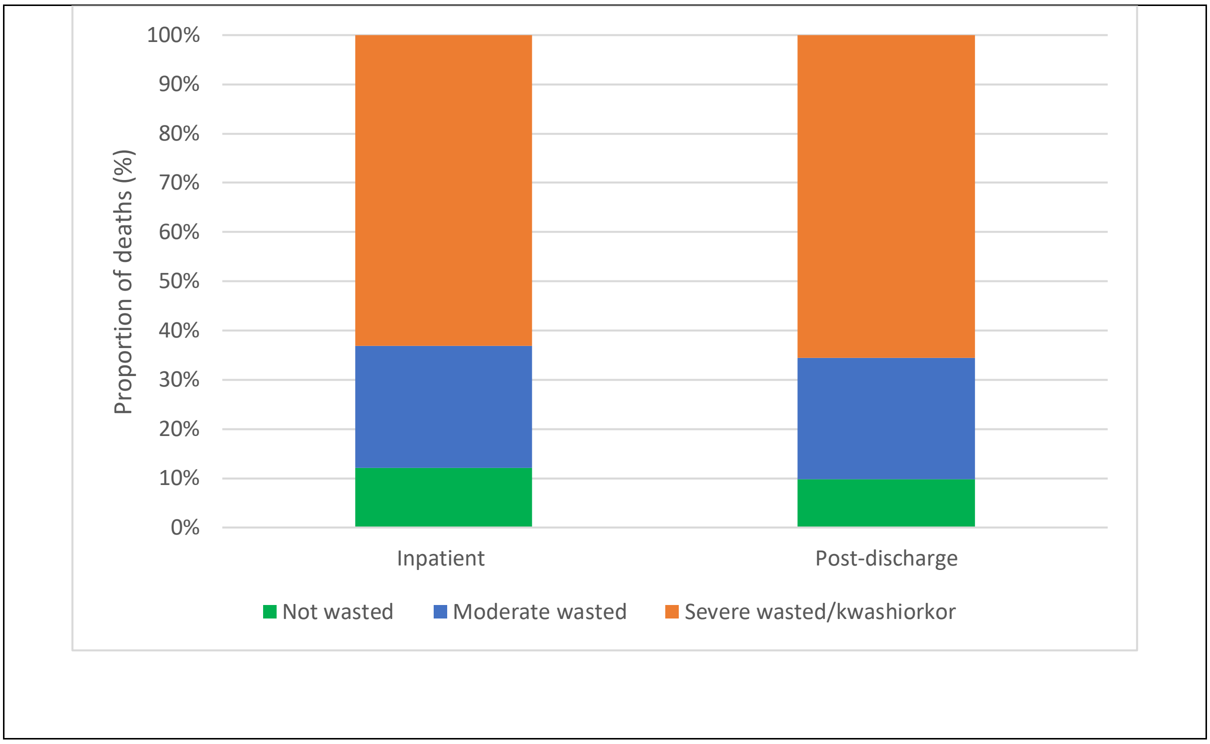

Figure S7. Cumulative hazard curves of mortality in the first 180 days after admission

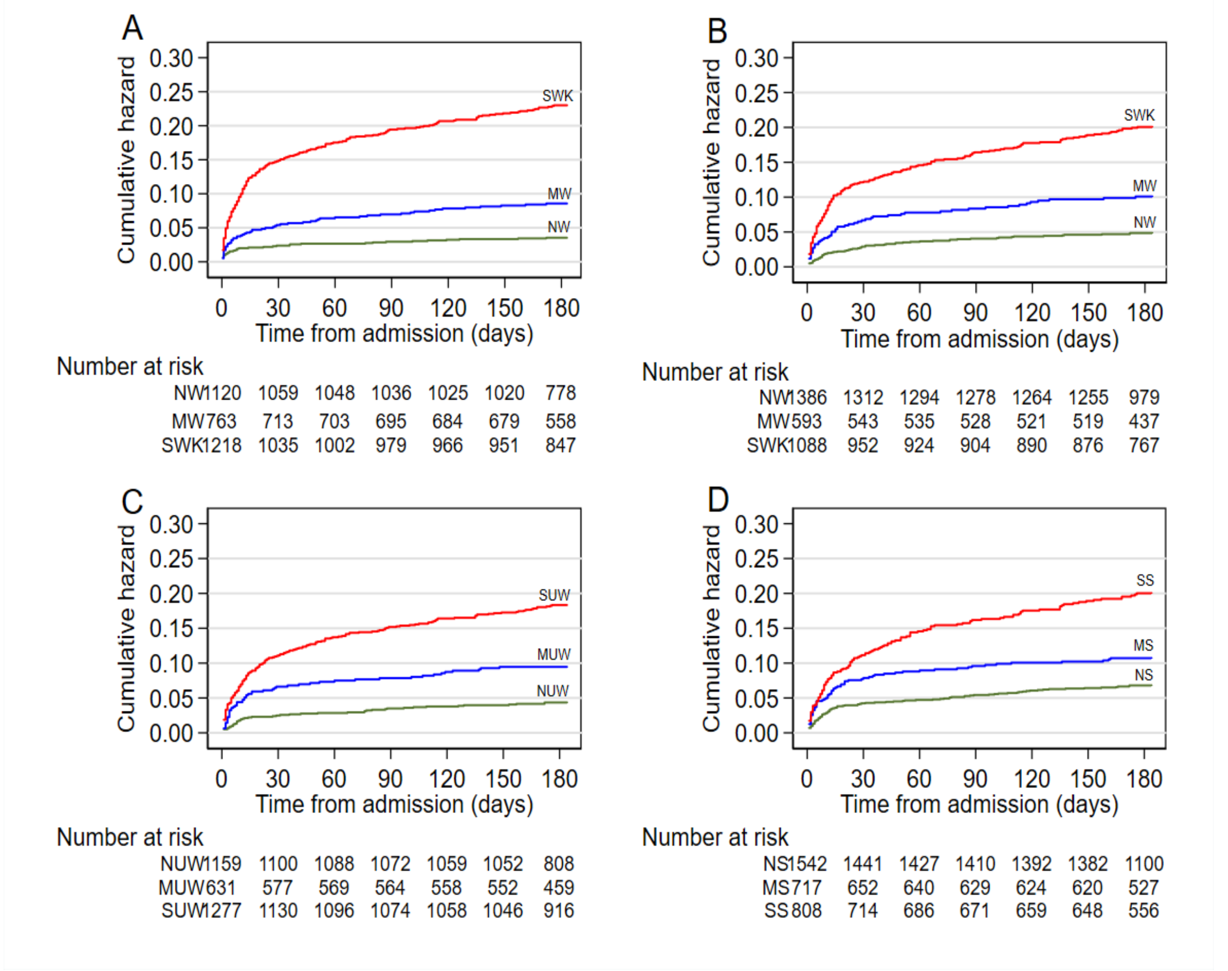

A-stratified by MUAC, B-stratified by weight-for-length z score, C-stratified by weight-for-age z score and D) stratified by length-for-age z score.

NW: Not wasted, MW: Moderately wasted, SWK: Severely wasted/Kwashiorkor; NUW: Not underweight, MUW: Moderately underweight, SUW: Severely underweight; NS: Not stunted, MS: Moderately stunted, SS: Severely stunted.

Figure S8. Predicted mortality by admission illness severity across continuous MUAC values.

A

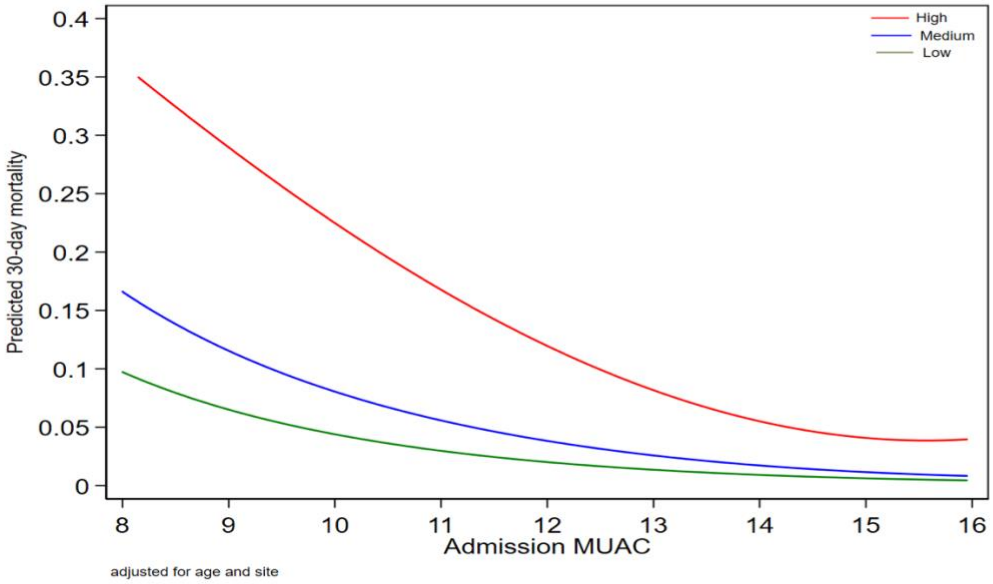

B

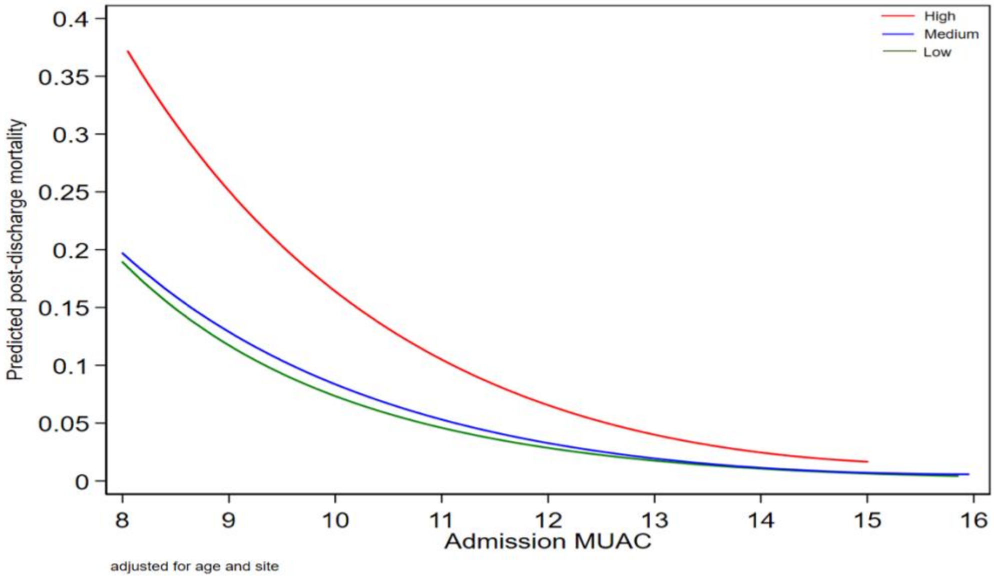

A) 30-day (P-values medium vs. low =0·04, high vs. low <0·001) and b) post-discharge mortality (P-values medium vs. low =0·69, high vs. low =0·001) by admission illness severity tertiles.

Figure S9. Admission Characteristics across quintiles of 30-day mortality regression predictions.

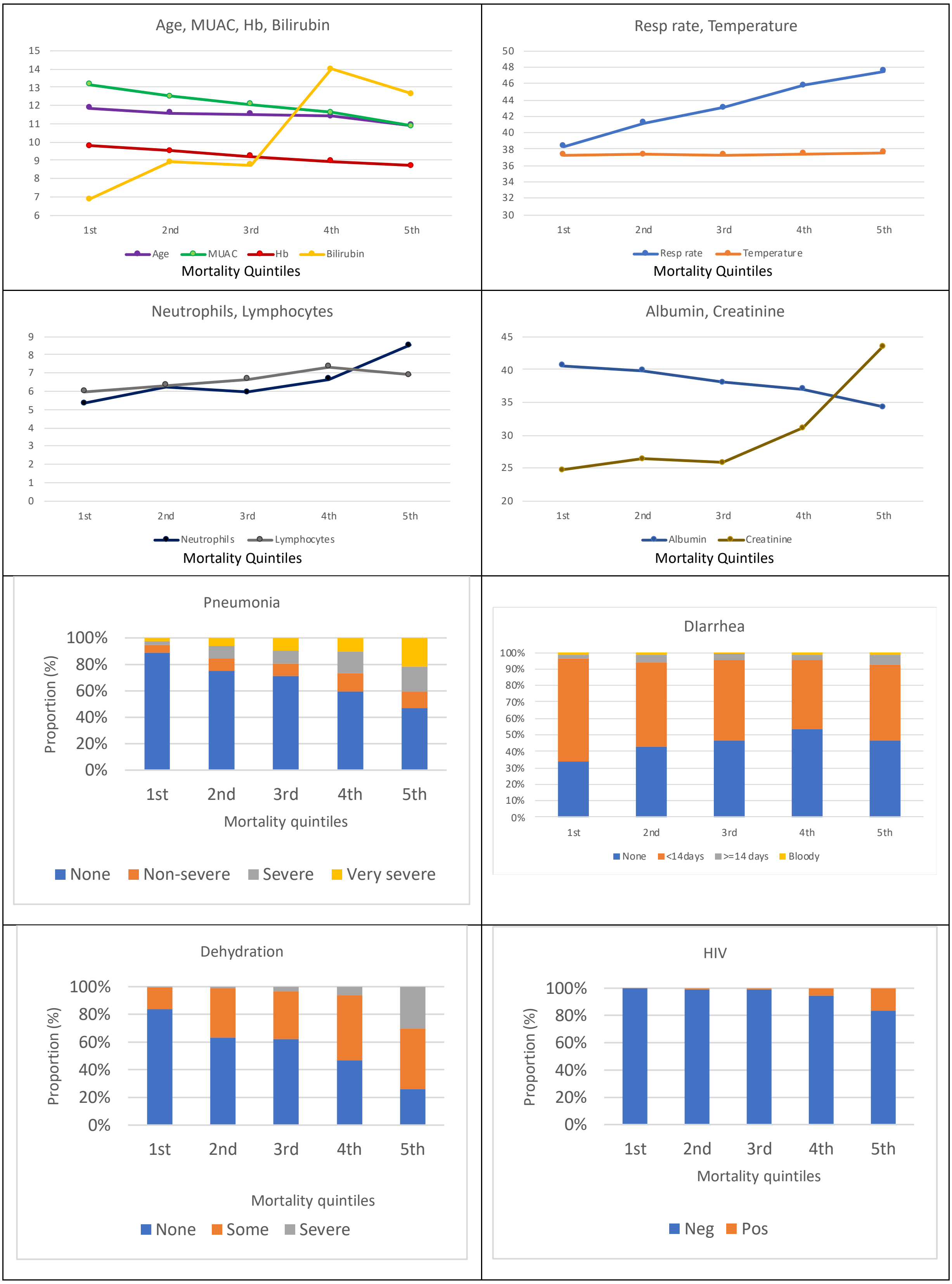

For the line graphs: the y-axis are the means, and the x-axis are the mortality quintiles. For the bar graphs: the y-axis is the proportion of children (%) and the x-axis are the mortality quintiles. Units: Age (months); MUAC (cm), Hb (g/dl), bilirubin (µmol/L), respiratory rate (breaths per minute), temperature (°C), neutrophils (10^9/L), lymphocytes (10^9/L), albumin (g/L), creatinine (µmol/L).

Figure S10. Discharge Characteristics across quintiles of post-discharge mortality regression predictions.

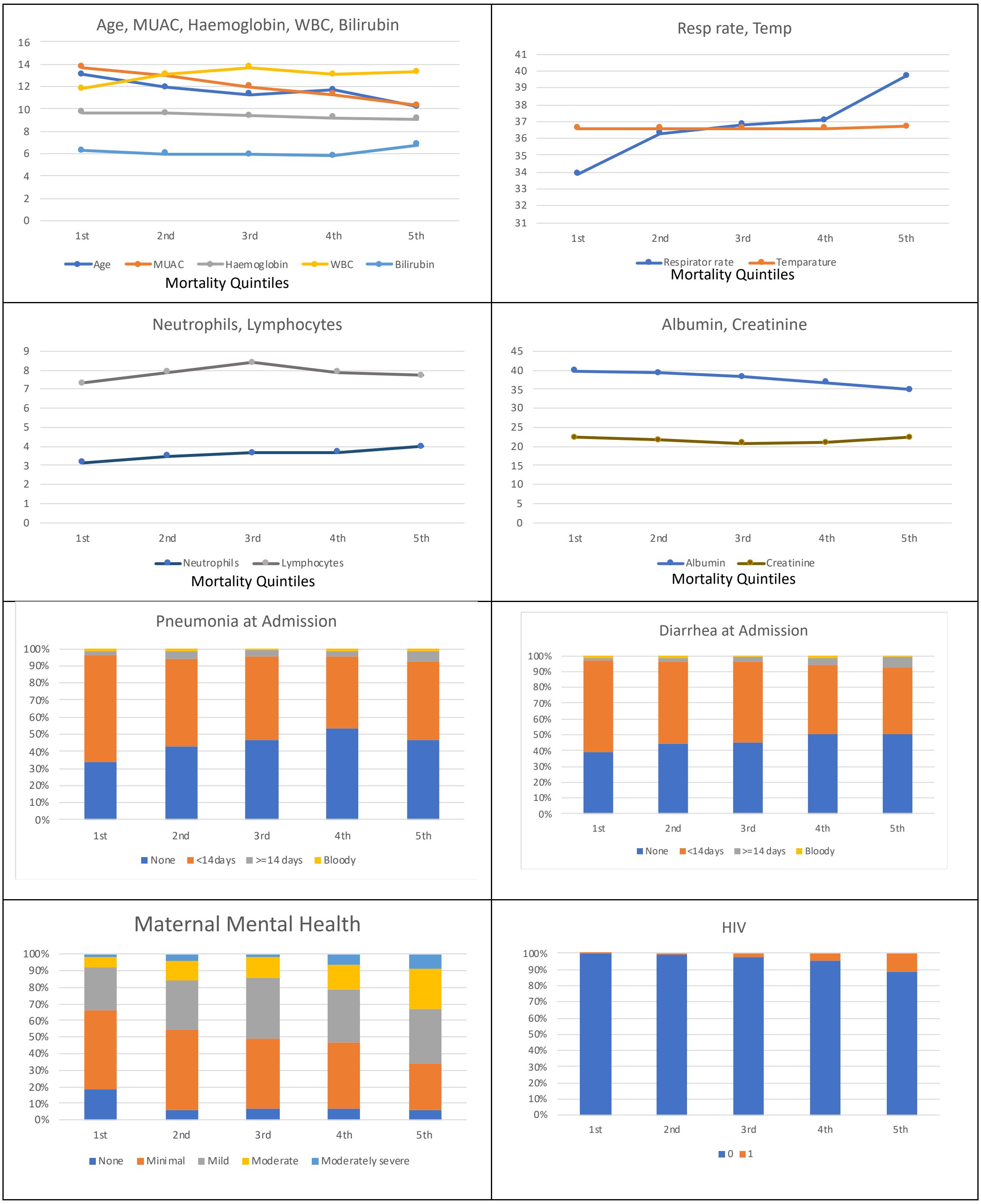

For the line graphs: the y-axis are the means, and the x-axis are the mortality quintiles. For the bar graphs: the y-axis is the proportion of children (%) and the x-axis are the mortality quintiles. Units: Age (months); MUAC (cm), Hb (g/dl), bilirubin (µmol/L), respiratory rate (breaths per minute), temperature (°C), neutrophils (10^9/L), lymphocytes (10^9/L), albumin (g/L), creatinine (µmol/L).

Statistical analysis plan

The Childhood Acute Illness Network

CHAIN Mortality Statistical Analysis Plan

**Version Control**	**Update after any change**
**Version 1·0**	Created by Moses Ngari 06 July 2019
**Version 1·1**	Jay Berkley & Moses Ngari 24 July 2019
**Version 1·2**	Jay Berkley & Moses Ngari 31 July 2019
**Version 1·3**	Jay Berkley & Moses Ngari 02 August 2019
**Version 1·4**	Jay Berkley & Moses Ngari 05 August 2019
**Version 1·5**	Moses Ngari 08 November 2019
**Version 1·6**	Jay Berkley & Moses Ngari 27 November 2019
**Version 1·7**	Jay Berkley, Daniella Brals & Moses Ngari 10 January 2020
**Version 1·8**	Complied all comments by Moses Ngari 20 March 2020.

This statistical analysis plan is prepared according to **STROBE** statement checklist for cohort studies.[1]

General principles

- The analysis plan will be agreed by the Childhood Acute Illness and Nutrition (CHAIN) Network directors and then circulated to the site principal investigators (PIs) for comments before the final analysis is undertaken.
- All queries will be resolved, and data quality control will be completed before analysis.
- The analysis will include all eligible hospitalized participants aged 2 to 23 months on or before 31st January 2019.
- Main analyses, sub-analyses and tests of interactions will be pre-specified.
- The power of any sub-group or secondary analyses may be insufficient.
- Since this is a multicenter stratified cohort (stratified non-proportionately by Mid-Upper Arm Circumference (MUAC) and oedema at admission), all analysis will be stratified by, or adjusted for enrolment anthropometric strata. Unmeasured characteristics of sites will be addressed through multilevel or frailty methods.
- This SAP includes baseline characteristics, Causal pathways analyses using structural equation modelling and survival analyses for the first main paper from CHAIN, focused on mortality.
- Other CHAIN planned analyses will have separate SAPs.

1. Background

Nutritional status is known to be strongly associated with childhood mortality risk, yet mechanisms linking anthropometry to risk are unknown. Among children with acute illness, it is not clear how the type of illness, its severity, background comorbidities, nutritional status and treatment contribute to outcomes and whether these may differ between inpatient and post-discharge mortality. In the post-discharge period, social circumstances and access to care may also be important determinants of risk. In prior studies, differences in design have impaired our ability to make meaningful comparisons between sites. Understanding risks after a standard of care is applied will help identify needs for further characterizing risk and potential new interventions to deliver effective care.

2. Objectives
   2.1. General objective

Paper 1 aims to provide a comprehensive perspective on early and late mortality in relation to nutritional status among acute ill children.

   2.2. Specific objectives

Among children aged 2 to 23 months admitted to hospital with acute illness across enrolment strata we aim to determine:

- Case fatality ratios and mortality rates over defined timescales
- Where and when deaths occurred: during index admission and post-discharge in the community or during readmission, the proportion of deaths during index admission and post-discharge
- Mortality for common clinical syndromes
- Demographic, clinical, laboratory and social exposures associated with mortality
- Causal pathways between ‘underlying causes’ and ‘immediate causes’ associated with mortality (see Figure 1)
- The main drivers of mortality within domains of characteristics
- Which domains and drivers are most amenable to targeted interventions to reduce mortality?

Figure 1: CHAIN Conceptual Framework

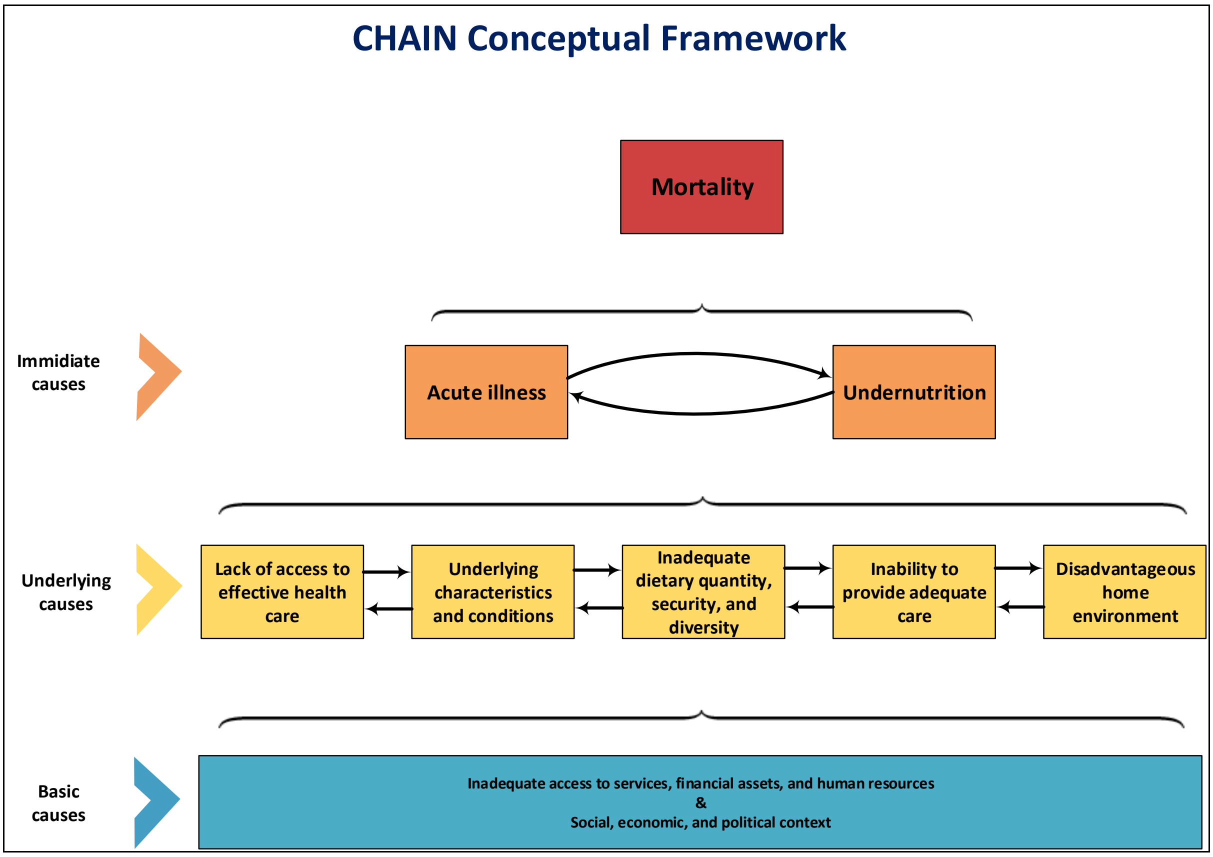

Notes: Adapted from UNICEF Conceptual Framework of Malnutrition.[2]

3. Study Design

The CHAIN Cohort is designed as a prospective non-proportional stratified cohort study. Children were under observation during their index hospital admission which has variable duration and for 180 days after discharge, a fixed duration, allowing analysis of inpatient case fatality ratio, and rates of short-term and post-discharge mortality over defined time periods.

3.1. Setting

Nine sites in low and middle-income countries (LMICs) (Bangladesh: Dhaka Hospital, Matlab Hospital, Burkina Faso: Banfora Referral Hospital, Kenya: Kilifi County Hospital; Mbagathi Sub-County Hospital, Nairobi; Migori County Hospital, Malawi: Queen Elizabeth Hospital, Blantyre, Pakistan: Civil Hospital, Karachi and Uganda: Mulago Hospital, Kampala). These sites all serve vulnerable populations and represent a range of environments, populations, access and levels of background comorbidities such as malaria and HIV.

3.2. Participants

CHAIN recruited children at admission to hospital in three strata to ensure a spectrum of nutritional status. The comprehensive nature of data collection, sampling and follow up meant limiting the rate of enrolment to address data quality and workload. Participants were identified by choosing the first admissions from a specified day each week until the weekly quota for each stratum was met. Children were treated according to current national and international clinical guidelines.

3.2.1. Inclusion criteria

- Aged 2 to 23 months
- Admitted to a study hospital

- Planning to remain in the hospital catchment area for at least 6 months and willing to come for the specified follow up visits
- Informed consent

3.2.2. Exclusion criteria

- Requiring immediate resuscitation at admission defined by ongoing cardiac or pulmonary arrest or judged to be peri-arrest by the attending physician.
- Unable to tolerate oral feeds while in his/her usual state of health
- Underlying terminal illness that in the opinion of the treating physician is likely to lead to death within 6 months (e.g., cancer, congenital heart disease)
- Diagnosed with a condition that in the opinion of the treating physician is likely to require surgery within 6 months
- Diagnosed chromosomal abnormality (syndromically or genetically diagnosed abnormality)
- Primary reason for admission is poisoning, trauma or a surgical condition
- Caregiver plans to move outside of the hospital catchment area within 6 months
- Caregiver is unwilling to attend study visits
- Previously enrolled in this study
- Sibling currently or previously enrolled in this study

3.2.3. Stratification

Children were enrolled in three strata classified at hospitalization:

- Severely wasted/kwashiorkor (SWK): MUAC <11·5cm (MUAC <11·0cm under 6 months old) or kwashiorkor.
- Moderately wasted (MW): MUAC 11·5 to <12·5cm at any age or MUAC 11·0 to <12·0cm under 6 months old
- Not wasted (NW): MUAC ≥12·5cm at any age or (MUAC 12cm or more under 6 months old

3.2.4. Timelines

Recruitment began on 20th November 2016 and ended on 31st January 2019 with 180 days post index hospital discharge follow up and defaulter tracing until 31st August 2019.

3.3. Variables

3.3.1. Outcomes

- Mortality (primary outcome) from index hospital admission until 180 days after discharge from index admission. Vital status and date of deaths were confirmed from hospital records, contact with parents/caregivers at hospital or at home, verbal autopsy, or observing a burial permit or death certificate.
- Causes of death were estimated from all available CHAIN data, inpatient records, inpatient verbal autopsy (VA) community VA using the standard WHO tool by two experience paediatricians.

3.3.2. Exposures

All exposure variables were collected in a standardized Case Report Form (CRF) using study standard operation procedure (SOP)s by trained staff.

- Demographics
- Anthropometry
- Clinical features at enrolment and discharge from the index admission

- Clinician’s diagnosis at enrolment and discharge from the index admission
- Routine laboratory investigations at enrolment and discharge from the index admission including complete blood count, HIV and malaria rapid tests
- Social, economic, maternal health, food security, and household characteristics during index hospital admission (collected after initial admission procedures up to 48 hours after admission)
- Social, economic, food security, and household characteristics collected at a home visit after discharge (excluding inpatient deaths) and used to verify the quality of data collected in hospital
- GPS location collected at a home visit after discharge (excluding inpatient deaths whose locations were estimated by a later visit to the area using a household location description)

3.3.3. Potential confounders and effect modifiers

- Age, sex will be included as *a priori* confounders in all models.
- Sites may have unobserved/unmeasured differences in accessibility, usage, populations, treatments available, staffing, clinical syndromes profile (unmeasured confounding effects). These will tested for and if necessary, multilevel model survival models will be constructed, accounting for non-independence of participants and effects of sites.[3]
- Enrolment strata will be included in in the models to determine their effects and reflect the stratified enrolment when considering effects of other co-variates.

3.4. Bias

Selection bias is recognized due to the enrolment strata known to be associated with mortality being purposively selected. This does not affect the primary analysis of mortality rates but may cause bias in observed effects of covariates interacting with nutritional status. Analyses of associations with mortality will be assessed by sensitivity analyses weighted inversely to estimates of the probability of being selected from all admissions in the study hospitals from available site data and existing literature, and interactions tested. A further sensitivity analysis examining a range of plausible weighting values may be conducted.

Attrition and outcome assessment bias due to loss to follow up or withdrawal (LTFU) was addressed during the study by minimizing LTFU through active follow ups, phone calls and home visits for children missing their scheduled clinical visits or absconding from hospital, including after the specified end of follow up period. Bias resulting from LTFU that may be associated with strata, site or other exposures will be adjusted for in primary and secondary analyses by weighting inversely to the probability of LTFU by site and enrolment strata giving 27 groups. A table of characteristics of individuals by LTFU status, a survival curve by anthropometric strata and LTFU weights will be tabulated or graphed as potential supplementary material.

To reduce reporting bias, we collected data on deaths that occurred outside the study hospitals during verbal autopsies conducted at home.

To reduce measurement bias, we standardized clinical care across the sites, training and assessment of clinical signs and definitions. Identical anthropometry equipment was centrally purchased, scales were calibrated, and measurements were performed by two independent observers and their arithmetic mean used in analysis. Data with implausible (absolute or relative to either other cohort participants or to the same participant measured at difference time points) were referred back to sites for resolution or set to ‘missing’ where unresolvable. A summary of implausible results will be tabulated by strata, site and age.

Missing data will be handled as follows for different type of variables:

Clinical and routine laboratory test: A `missing/test not done’ category will be added such as for routine tests like infant HIV, malaria RDT or variables where pre-defined categories are useful to the analysis, such as haemoglobin.[4] For variables used in continuous form, such as blood glucose, we may include a dummy variable for missing data. For

oxygen saturation, children with unrecordable value despite efforts to measure it or were measured while in oxygen therapy will be classified as having hypoxia.

Anthropometry: The few missing anthropometries at admission (4 weights, 10 heights and 7 head circumferences), will be checked from child's admission records or the daily records collected by the study team. Where height is missing at discharge, admission values will be used where possible. Where admission height is missing, discharge height will be used.

Socioeconomic variables: Children who died early (first 48 hours) did not have socioeconomic data collected because collection of these data was not prioritized at the time of hospitalization. Therefore, missing these data was associated with early death. Thus, for early deaths analysis (first 30 days), we will create a model with 'a missing' category for these data. A sensitivity analysis will also be conducted assuming an optimum value for missing data (best case scenario). The post-discharge analysis will use 'a missing' category for all the missing data.

Laboratory variables: Complete blood count (haemoglobin and white blood cells) will be categorized according to WHO and paediatric sepsis guidelines,[5] with a category for missing values.

3.5. Study Size

The study was designed to have a power of 80% to detect differences in proportion of children who would die post-discharge between non-wasted and moderately wasted children, with α=0.05 and allowing up to 10% loss to follow-up. The final version of the study protocol specified at least 2,600 children to be discharged alive and followed up post-discharge. In the actual study database, 3,101 acutely ill children were enrolled, and 2,868 children were followed post-discharge. Crude proportions of those who died post discharge were:

Group	% died post discharge	P value for Hazards Ratio
**NW**	1·67	Reference
**MW**	4·09	0·002
**SW/K**	11	<0·001

3.6. Quantitative variables

- Small birth size will be defined as either low birth weight (birth weight <2.5kg) or estimated to have been born premature (gestation age <37 weeks) since actual birthweight was unknown by parents/guardians in approximately a third of participants.
- Anthropometric z-scores will be computed using WHO 2006 references and where appropriate categorized into severe (<-3), moderate (-3 to -2) and normal (≥-2).
- Disease syndromes will be constructed following definitions used in WHO treatment guidelines. [6]
- Functional forms of continuous independent variables will be examined using test for linearity and, if needed, fractional polynomial functions or cubic splines. Independent variables with non-linear relationship with mortality will either be transformed using appropriate methods or grouped into biologically plausible categories or categories by WHO standard thresholds (e.g. haemoglobin).[4]
- Haemoglobin and oxygen saturation will be corrected for altitude[7].
- Systemic inflammatory response syndrome (SIRS) will be defined by two or more symptoms including hypothermia or fever, tachypnoea, tachycardia and change in white blood cells count[5].
- Water sanitation and hygiene (WASH) variables will be grouped into improved and not improved following WHO and Demographic and Health Survey (DHS) criteria [8].
- An asset index will be derived using principal component analysis (PCA) by including household assets ownership and housing structure variables and categorized into five quintiles [9,10].
- Data on caregiver's mental health will be summarized into a total score by summing the responses of all the nine questions in the PHQ-9 [11,12].This will further be categorized into three groups indicating the degree of features of depression for analysis; 1) minimal (0 to 9), 2) moderate (10 to 14) and 3) severe (≥15).

- A food insecurity variable will be derived using normalized scores and categorized into three groups: low, medium and high [13-16].
- Appropriate diet variable was defined as: exclusively breastfed for children <6 months, more than or equal to two food groups and breastmilk for children 6 to 9 months and more than or equal to four food groups plus breastmilk for children 10 to 23 months.
- GPS location and estimates made from the population density, distance to the study hospital and nearest health facility using standard methods.

4. Statistical methods
   4.1. Participants

A flow chart (Figure 1) will detail the numbers of (stratified by enrolment strata):

Patients screened
Excluded
Enrolled
Withdrew before discharge from the index admission
LTFU before discharge from the index admission
Died before discharge from the index admission
Discharged alive from the index admission and followed up
Withdrew after discharge from the index admission
LTFU after discharge from the index admission
Died after discharge from the index admission
Alive at day 180 after discharge from the index admission

4.2. Baseline characteristics

Baseline characteristics by enrolment strata will be described using N and proportions. For continuous variables including age and anthropometric measurements, mean (sd) and median (IQR), depending on distribution (Table 1) will be reported. The count and proportion of variables with missing data used in the analysis will be reported. Baseline characteristics by site will be provided as supplemental materials. Similar characteristics at discharge of index admission will be provided in the supplemental materials.

4.3. Follow up

Total participants, percentage (LTFU + withdrawn separately) and rate of LTFU (LTFU + withdrawn) per stratum will be reported and tested for differences across the enrolment strata.

Person-time follow up for the periods given in section 5.6 below will be calculated and mortality rates computed per 1000 child-months.

4.4. Outcome data

Primary outcome, mortality will be analyzed as summarized in the table in section 5.6 below.

4.5. Main results

- Unadjusted numbers of events and proportions
- Unadjusted rates, incidence rate ratios by enrolment strata adjusted for age, sex and site, weighted for observed LTFU

- Table of causes of death, place and timing of post-discharge deaths.
- Table of mortality by common clinical syndromes including Malaria, HIV, severe pneumonia, diarrhoea, sepsis and severe anaemia.
- Test modification of effects of enrolment strata on mortality by site using Mantel-Cox or similar method.
- Multivariable (multilevel) survival models to determine factors associated with outcomes during specific periods:
  - Model 1: Early mortality; time at risk from study enrolment to day 30 using enrolment data
  - Model 2: Post-discharge mortality; time at risk from index hospital discharge to 180 days later using discharge data, social data and enrolment data
- Interactions between key variables and continuous MUAC, e.g., illness severity (SIRS/organ dysfunction), stunting, HIV (supplementary)

4.6. Summary of analyses

	Time period	Numeric outcome	Rate and person-days observed	HR for anthropometric strata	Factors associated with mortality
**All deaths from admission to 180 days after discharge**	Variable	N (%) of participants who died at any time during follow up	No	No	No
**Deaths during index admission**	Variable	N (%) of participants who died at any time during index admission	No (give median IQR of duration of hospitalisation)	No	No
**Deaths during first 2 days**	Fixed	N (%) of participants who died <3 days after enrolment	No	No	No
**Deaths during first 30 days**	Fixed	N (%) of participants who died <31 days after enrolment	Yes	Yes	Yes (M1)
**Deaths from discharge to 180 days later**	Fixed	N (%) of participants who died at any time after discharge from the index admission and proportion of deaths occurring post-discharge	Yes	Yes	Yes (M2) (including admission and discharge data)
**Deaths during first 180 days (supplementary)**	Fixed	N (%) of participants who died <181 days after enrolment	Yes	Yes	No (less useful for policy & time varying exposures)

4.7. Regression models - methods

4.7.1. Survival analysis

For the evaluation of the primary outcome of mortality in relation to enrolment strata (Groups SWK and MW, with NW as the reference), the initial **base model** will include *a priori* measured confounders. We will test for evidence of unobserved heterogeneity across the sites using likelihood ratio test and account for it as a random effect (if necessary). If there is no evidence of unobserved heterogeneity across the sites, we will account for clustering within sites. The *priori* confounders are:

- Age (continuous)
- Sex
- Site as a random effect
- Change in MUAC between index admission and discharge (for post-discharge analyses)
- Index discharge kwashiorkor (for post-discharge analyses)

The multilevel multivariate regression model building will entail four stages:

a) Within each domain, univariate model for every independent variable adjusting only for the *priori* confounders will be built. Individual independent variable with a P-value <0.1 in the univariate model will be selected for inclusion in the next stage.
b) All the individual independent variables selected in each domain will be included in the domain multivariate model. The domain multivariate model will exclude the *priori* confounders because they will already have been adjusted for. However, the models will use multilevel approach with sites as random effect.
c) After running each domain multivariate model, the predicated risk of mortality will be calculated and converted into five quintiles scores.
d) The final regression model will include the enrolment strata (NW, MW and SWK), the *priori* confounders and all the domain quintiles scores with site as a random effect.

We will assess the multivariate regression models' goodness of fit using bootstrapped area under the receiver operating curves (AUROC) with a probit model, resampled 100 times with replacement.

Initially, Cox proportional hazard regression model will be assessed for its proportional hazard assumption using the Schoenfeld residuals method and ability to deal with sampling weights, control for site shared unobserved characteristics and lack of independence of observations within sites. Then, parametric multilevel survival analysis regression models will be considered by assessing their fit with specific parametric probability distribution functions and their robustness to deal with sampling weights, control for site shared unobserved characteristics and lack of independence of observations within sites. The common survival analysis parametric models (exponential, gamma, Weibull, and log-normal distribution) fit with the underlying probability distribution will be examined by comparing their predicated Cox–Snell residuals to assess concordance with the failure time or log failure time distribution and identify outlier observations. Additionally, the Akaike information criterion (AIC) and the Log likelihood test will be used, with the parametric model returning lowest AIC and highest Log likelihood value being selected.

4.7.2. Structural Equation Modelling

Figure 1 will form the basis for building the empirical SEM. Three different levels of mortality determinants can be defined according to this conceptual framework, namely (i) **basic causes** (consisting of the domains: inadequate access to services, financial assets, and human resources; and social, economic, and political context), (ii) **underlying causes** (consisting of the domains: underlying characteristics and conditions; inadequate dietary quantity, security and diversity; inability to provide adequate care; disadvantageous home environment; and lack of access to effective health care), and (iii) **immediate causes** (consisting of the domains: acute illness and undernutrition). As the CHAIN study was not designed to measure basic causes other than those pooled at site level, the analysis will focus on the underlying and immediate causes. Since CHAIN recruited children from nine hospitals across Africa and Asia, the varying unobserved heterogeneity in access to health care, a proxy of the basic causes, will be adjusted for in the analysis. As opposed to the survival analysis (see 5.7.1)–where we will investigate which underlying and immediate variables are predictors of mortality–the SEM approach will include (inter)relationships between variables and identify complex pathways leading up to mortality.

SEM building will consist of the following steps:

I. Review relevant literature to support model specification (see Figure 1):
II. Model specification (see an example specification in Figure 2):
    a. It is assumed that only immediate causes will lead directly to mortality, but other paths leading directly to mortality not passing through acute illness or nutritional status will also be investigated (since acute illness and nutritional status are both only measured at a single time point);
III. Select measures for the domains/variables represented in the model
    a. Underlying and immediate domains will be included as latent variables/factors (see the ovals in Figure 2);
    b. Latent variables/factors will be estimated by using Explanatory and Confirmatory Factor Analyses, were we estimate the paths that link each observed variable/item to their corresponding (unobserved) latent variable/factor;

c. The a-priori confounders (age and sex) will be included in the *underlying conditions* latent class (where the study was specifically designed to include only children <2 yrs. at the time of index admission);
d. We will adjust for basic causes by either:
    1. Clustering within site;
    2. Adding site as a random effect in a hierarchical SEM;

IV. Conduct preliminary descriptive statistical analysis (e.g., scaling, missing data, collinearity issues, outlier detection, linearity, multivariate normality)
V. Estimate the SEM parameters (steps V. – VII. may be repeated several times);
VI. Assess model fit [17];
VII. Re-specify the model if necessary (e.g. only retain significant paths in the SEM, see the arrows in Figures 2);
VIII. Interpret and present results (i.e. add values to the paths in Figures 2, as well as sub-figures detailing the paths to latent variables/factors, and make a supplementary table with the full estimation results);
IX. Compare changes in predicted mortality probabilities per child when dropping a latent variable/class from the SEM, compared to the full SEM. This will inform in which domain potential interventions are going to make the biggest difference in survival.

As we want the SEM findings to complement the survival analysis results, we will build a single SEM for 30-day mortality (binary outcome) and a single SEM for 180-day mortality (binary outcome). In addition, we will try to estimate the above two SEM models using time to mortality as outcome (instead of binary mortality as outcome). In addition, we will consider building a SEM for mortality at end line (uniting the two single SEMs), taking specifically the time dimension into account.

Figure 2: Example of a SEM framework

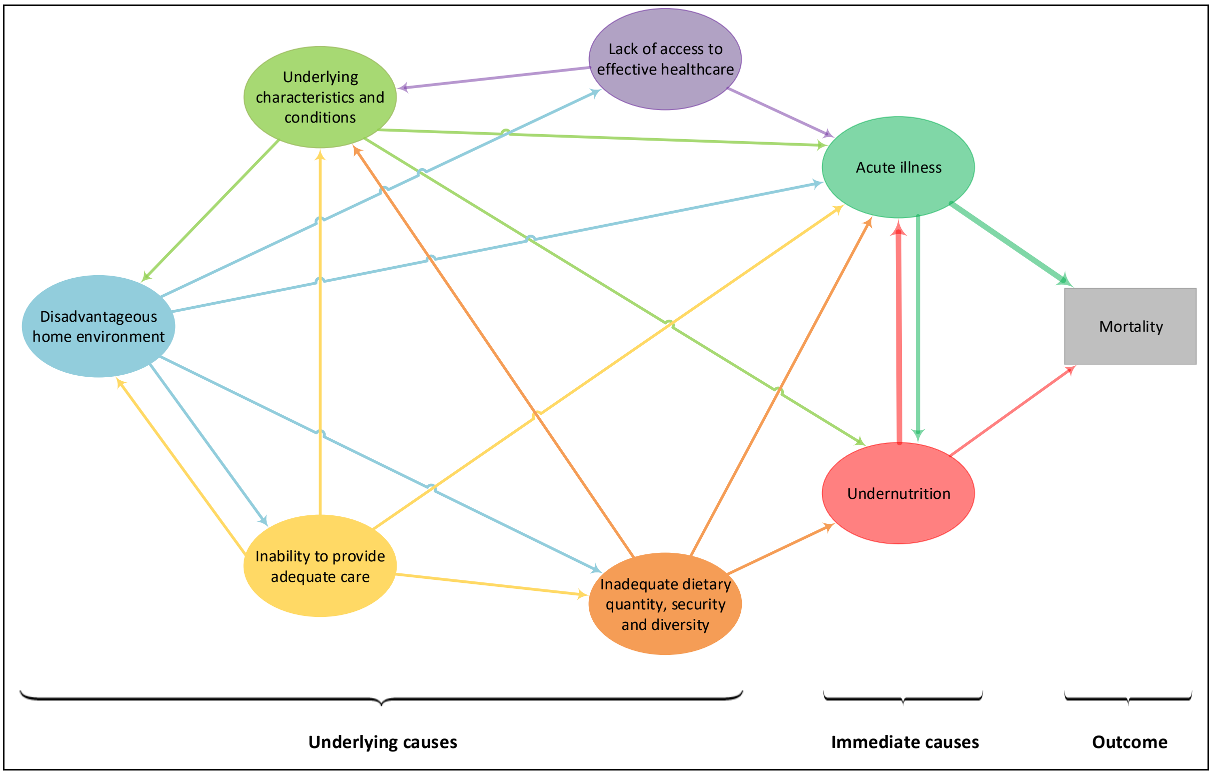

4.8. Prespecified sub analyses

a) Effect modification of nutritional status on relationship between sites and mortality will be tested using Mantel-Cox or likelihood ratio test methods.

b) To test the effect of HIV status on the association between admission nutritional status and mortality, we will include terms of interactions and compare models with and without interaction term using likelihood-ratio χ^2 tests.

c) The SWK group consist of children who are severely wasted (low MUAC only) and those with kwashiorkor who might have varying risk of mortality. We therefore, will have a separate sub analysis with four nutrition strata; NW, MW, severely wasted (low MUAC only) and Kwashiorkor.

SAP References

1. von Elm E, Altman DG, Egger M, Pocock SJ, Gotzsche PC, Vandenbroucke JP, et al. The Strengthening the Reporting of Observational Studies in Epidemiology (STROBE) statement: guidelines for reporting observational studies. J Clin Epidemiol. 2008;61(4):344-9.
2. UNICEF. Improving Child Nutrition: The achievable imperative for global progress. 2013.
3. Martins A, Aerts M, Hens N, Wienke A, Abrams S. Correlated gamma frailty models for bivariate survival time data. Stat Methods Med Res. 2018:962280218803127.
4. (WHO) WHO. Haemoglobin concentrations for the diagnosis of anaemia and assessment of severity 2011 Available from: http://apps.who.int/iris/bitstream/10665/85839/3/WHO_NMH_NHD_MNM_11.1_eng.pdf
5. Comstedt P, Storgaard M, Lassen AT. The Systemic Inflammatory Response Syndrome (SIRS) in acutely hospitalised medical patients: a cohort study. Scand J Trauma Resusc Emerg Med. 2009;17:67.
6. WHO. Pocket book of Hospital Care for Children: WHO Press; 2013. Available from: http://www.who.int/maternal_child_adolescent/documents/child_hospital_care/en/.
7. Sullivan KM, Mei Z, Grummer-Strawn L, Parvanta I. Haemoglobin adjustments to define anaemia. Tropical medicine & international health : TM & IH. 2008;13(10):1267-71.
8. World Health Organization, UNICEF. Water for life: Making it happen. Geneva2005.
9. Rutstein S, Kiersten J. The DHS Wealth Index. DHS Comparative Reports No. Calverton, Maryland2004. p. 1-77.
10. Vyas S, Kumaranayake L. Constructing socio-economic status indices: how to use principal components analysis. Health Policy and Planning. 2006;21(6):459-68.
11. Kroenke K, Spitzer RL. The PHQ-9: a new depression diagnostic and severity measure. Psychiatry Annals. 2002;32:509-21.
12. Kroenke K, Spitzer RL, Williams JB. The PHQ-9: validity of a brief depression severity measure. J Gen Intern Med. 2001;16(9):606-13.
13. Ballard TJ, Kepple AW, Cafiero C. The Food Insecurity Experience Scale: Development of a Global Standard for Monitoring Hunger Worldwide Rome: FAO; 2013 [Available from: http://www.fao.org/economic/ess/ess-fs/voices/en/.
14. Ballard TJ, Kepple AW, Cafiero C, Schmidhuber J. Better measurement of food insecurity in the context of enhancing nutrition. Emahrungs Umschau. 2014;61(2):38-41.
15. Cafiero C, Viviani S, Nord M. Food security measurement in a global context: The food insecurity experience scale. Measurement. 2017.
16. Wambogo EA, Ghattas H, Leonard KL, Sahyoun NR. Validity of the Food Insecurity Experience Scale for Use in Sub-Saharan Africa and Characteristics of Food-Insecure Individuals. Current Developments in Nutrition. 2018:1-10.
17. Randall E. Schumacker RGL. A Beginner's Guide to Structural Equation Modeling: Lawrence Erlbaum Associates, 2004; 1996. 498 p.

SAP Appendix 1: Domains

Domain	Variables included
**Base model**	Nutritional status (NW, MW, SWK)<br>Age in months<br>Sex (Male, Female)<br>Change in MUAC from admission to discharge<br>Discharge Kwashiorkor
**Underlying characteristics and conditions**	Birth size<br>HIV status<br>Stunting level<br>Prior hospitalization<br>Sickle cell disease<br>Population density
**Acute illness**	SIRS<br>Respiratory distress (none, moderate, severe)<br>Circulation distress (shock) (None, some, and all signs of shock)<br>Neurological (AVPU)<br>Dehydration (none, some, severe)<br>Blood glucose (normal, low, high)<br>Severe anaemia
**Access to health care**	Travel time to the hospital (30-day mortality)<br>Distance to the nearest health facility<br>Travel to hospital cost (US dollars)<br>Means of travel to hospital<br>Walking/ Tuktuk/ Rickshaw/ Motorbike<br>Ambulance<br>Private means<br>Public means<br>Others
**Household-level exposures**	Recommended adequate diet (No, yes)<br>Assets quintiles (5 levels)<br>Food insecurity (low, medium, high)<br>Type of toilet (improved, not improved)<br>Water availability (No, yes)<br>Keep livestock (No, yes)
**Caregiver characteristics**	Biological mother as primary caregiver<br>Caregiver age in years<br>Caregiver education level<br>Caregiver employment<br>Mother mental health<br>Mother sick or pregnant

SIRS; Systemic Inflammatory Response Syndrome was derived from temperature, heart rate, respiratory rate and white blood counts at admission.

STROBE Statement

SAP Appendix 2

STROBE Statement—Checklist of items that should be included in reports of ***cohort studies***

	Item No	Recommendation
Title and abstract	1	(*a*) Indicate the study's design with a commonly used term in the title or the abstract (P1)
		(*b*) Provide in the abstract an informative and balanced summary of what was done and what was found (ABSTRACT P2)
Introduction		
Background/rationale	2	Explain the scientific background and rationale for the investigation being reported (INTRODUCTION P6)
Objectives	3	State specific objectives, including any prespecified hypotheses (INTRODUCTION P7 & STATISTICAL ANALYSIS PLAN)
Methods		
Study design	4	Present key elements of study design early in the paper (METHODS P7)
Setting	5	Describe the setting, locations, and relevant dates, including periods of recruitment, exposure, follow-up, and data collection (METHODS P7/8)
Participants	6	(*a*) Give the eligibility criteria, and the sources and methods of selection of participants. Describe methods of follow-up (METHODS P8, TEXT BOX)
		(*b*) For matched studies, give matching criteria and number of exposed and unexposed N/A
Variables	7	Clearly define all outcomes, exposures, predictors, potential confounders, and effect modifiers. Give diagnostic criteria, if applicable (METHODS P6/7, TABLE 1, SUPPLEMENTARY APPENDIX P2-5)
Data sources/ measurement	8*	For each variable of interest, give sources of data and details of methods of assessment (measurement). Describe comparability of assessment methods if there is more than one group (METHODS P9/10, TABLE 1, SUPPLEMENTARY APPENDIX P2-5)
Bias	9	Describe any efforts to address potential sources of bias (METHODS P9, STATISTICAL ANALYSIS PLAN)
Study size	10	Explain how the study size was arrived at (METHODS P9)
Quantitative variables	11	Explain how quantitative variables were handled in the analyses. If applicable, describe which groupings were chosen and why (METHODS P9/10, STATISTICAL ANALYSIS PLAN)
Statistical methods	12	(*a*) Describe all statistical methods, including those used to control for confounding (METHODS P9/10, STATISTICAL ANALYSIS PLAN)
		(*b*) Describe any methods used to examine subgroups and interactions (METHODS P10, STATISTICAL ANALYSIS PLAN)
		(*c*) Explain how missing data were addressed (SUPPLEMENTARY APPENDIX P10, TABLE S1, STATISTICAL ANALYSIS PLAN)
		(*d*) If applicable, explain how loss to follow-up was addressed (METHODS P8, STATISTICAL ANALYSIS PLAN)
		(*e*) Describe any sensitivity analyses (METHODS P10, STATISTICAL ANALYSIS PLAN)
Results		
Participants	13*	(a) Report numbers of individuals at each stage of study—eg numbers potentially eligible, examined for eligibility, confirmed eligible, included in the study, completing follow-up, and analysed (RESULTS P11, STUDY FLOW FIGURE S2)
		(b) Give reasons for non-participation at each stage (RESULTS P11, STUDY FLOW FIGURE S2)
		(c) Consider use of a flow diagram (STUDY FLOW FIGURE S2)
Descriptive data	14*	(a) Give characteristics of study participants (eg demographic, clinical, social) and information on exposures and potential confounders (TABLE 1, SUPPLEMENTARY TABLES S7-S15)
		(b) Indicate number of participants with missing data for each variable of interest (TABLE 1)
		(c) Summarise follow-up time (eg, average and total amount) (RESULTS P11, FIGURE 1)
Outcome data	15*	Report numbers of outcome events or summary measures over time (RESULTS P11/12)

	16	(*a*) Give unadjusted estimates and, if applicable, confounder-adjusted estimates and their precision (eg, 95% confidence interval). Make clear which confounders were adjusted for and why they were included **(TABLE 3, TABLES S24-S26)**
		(*b*) Report category boundaries when continuous variables were categorized **(TABLE 1, SUPPLEMENTARY APPENDIX P5)**
		(*c*) If relevant, consider translating estimates of relative risk into absolute risk for a meaningful time period **(RESULTS P11-13)**
Other analyses	17	Report other analyses done—eg analyses of subgroups and interactions, and sensitivity analyses **(RESULTS P14, TABLES S25-S30)**
**Discussion**		
Key results	18	Summarise key results with reference to study objectives **(DISCUSSSION P16)**
Limitations	19	Discuss limitations of the study, taking into account sources of potential bias or imprecision. Discuss both direction and magnitude of any potential bias **(DISCUSSSION P15)**
Interpretation	20	Give a cautious overall interpretation of results considering objectives, limitations, multiplicity of analyses, results from similar studies, and other relevant evidence **(DISCUSSSION P14/15)**
Generalisability	21	Discuss the generalisability (external validity) of the study results **(DISCUSSSION P17/18)**
**Other information**		
Funding	22	Give the source of funding and the role of the funders for the present study and, if applicable, for the original study on which the present article is based **(FUNDING P19)**

*Give information separately for exposed and unexposed groups.

Note: An Explanation and Elaboration article discusses each checklist item and gives methodological background and published examples of transparent reporting. The STROBE checklist is best used in conjunction with this article (freely available on the Web sites of PLoS Medicine at http://www.plosmedicine.org/, Annals of Internal Medicine at http://www.annals.org/, and Epidemiology at http://www.epidem.com/). Information on the STROBE Initiative is available at http://www.strobe-statement.org.

Supplementary References

1. Tickell KD, Mangale DI, Tornberg-Belanger SN, et al. A mixed method multi-country assessment of barriers to implementing paediatric inpatient care guidelines. PLoS One 2019; 14(3)
2. WHO Multicentre Growth Reference Study Group. WHO child growth standards : length/height-for-age, weight-for-age, weight-for-length, weight-forheight and body mass index-for-age : methods and development. 2006.
3. Kroenke K, Spitzer RL. The PHQ-9: A new depression diagnostic and severity measure. Psychiatr. Ann. 2002; ***32***(9), 509–515.
4. Kroenke K, Spitzer RL, Williams JBW. The PHQ-9: Validity of a brief depression severity measure. J Gen Intern Med 2001; 16(9)
5. Green EP, Tuli H, Kwobah E, Menya D, Chesire I, Schmidt C. Developing and validating a perinatal depression screening tool in Kenya blending Western criteria with local idioms: A mixed methods study. J Affect Disord 2018; 228:49-59.
6. Altaf A, Khan M, Shah SR, et al. Sociodemographic pattern of depression in urban settlement of Karachi, Pakistan. J Clin Diagnostic Res 2015; 9(6)
7. Gelaye B, Williams MA, Lemma S, et al. Validity of the patient health questionnaire-9 for depression screening and diagnosis in East Africa. Psychiatry Res 2013; 210(2)
8. Monahan PO, Shacham E, Reece M, et al. Validity/reliability of PHQ-9 and PHQ-2 depression scales among adults living with HIV/AIDS in Western Kenya. J Gen Intern Med 2009; 24(2)
9. Ballard T, Kepple A, Cafiero C. The food insecurity experience scale: development of a global standard for monitoring hunger worldwide. Technical Paper. Rome, FAO 2013. (available at http://www.fao.org/economic/ess/ess-fs/voices/en/).
10. Cafiero C, Viviani S, Nord M. Food security measurement in a global context: The food insecurity experience scale. *Measurement*, 116, February 2018, pp. 146-152. (available at https://www.sciencedirect.com/science/article/pii/S0263224117307005)
11. Wambogo EA, Ghattas H, Leonard KL, Sahyoun NR. Validity of the Food Insecurity Experience Scale for use in sub-Saharan Africa and characteristics of food-insecure individuals. Curr Dev Nutr 2018; 2(9)
12. WHO. Global Nutrition monitoring framework. Operational guidance for tracking progress in meeting targets for 2025. Geneva: World Health Organization; 2017. (available at https://www.who.int/publications/i/item/9789241513609)
13. UNICEF, WHO. Water for life: making it happen. Jt Monit Progr Water Sanit 2005. (available at https://www.who.int/water_sanitation_health/monitoring/jmp2005/en/)
14. Florey L, Taylor C. Using household survey data to explore the effects of improved housing conditions on malaria infection in children in Sub-Saharan Africa. 2016. (avaliable at https://dhsprogram.com/publications/publication-as61-analytical-studies.cfm)
15. Rysankova D, Putti VR, Hyseni B, Kammila S, Kappen JF. Clean and improved cooking in sub-Saharan Africa: A landscape report. 2014. (available at https://openknowledge.worldbank.org/handle/10986/22521)
16. Rutstein, S. O., Johnson, K. The DHS Wealth Index. DHS Comparative Reports No. 6. Calverton, Maryl ORC Macro 2004. (avaiable at https://dhsprogram.com/publications/publication-cr6-comparative-reports.cfm)
17. Josse J, Husson F. Handling missing values in exploratory multivariate data analysis methods. J la Société Française Stat 2012.
18. Josse J, Husson F. missMDA: A package for handling missing values in multivariate data analysis. J Stat Softw 2016.

19. Rutstein SO, Staveteig S. Making the Demographic and Health Surveys Wealth Index comparable. 2014. (available at https://dhsprogram.com/publications/publication-mr9-methodological-reports.cfm)

20. Current and Historical Rate Tables [Internet]. (available at https://www.xe.com/currencytables/)

21. Global Health Cost Consortium (GHCC). Reference case for estimating the costs of global health services and interventions. [Internet]. 2017. (available at https://ghcosting.org/pages/standards/reference_case)

22. Goldstein B, Giroir B, Randolph A. International paediatric sepsis consensus conference: Definitions for sepsis and organ dysfunction in paediatrics. In: Paediatric Critical Care Medicine. 2005. 6(1):2-8.

23. WHO. Pocket Book of Hospital Care for Children: Guidelines for the Management of Common Childhood Illnesses. 2013. (avaibale at https://www.who.int/maternal_child_adolescent/documents/child_hospital_care/en/)

24. WHO. Haemoglobin concentrations for the diagnosis of anaemia and assessment of severity. Vitamin and Mineral Nutrition Information System. Geneva, World Health Organization, 2011 (avaiable at http://www.who.int/vmnis/indicators/haemoglobin.pdf), acc. 2011)

25. Harris PA, Taylor R, Thielke R, Payne J, Gonzalez N, Conde JG. Research electronic data capture (REDCap)-A metadata-driven methodology and workflow process for providing translational research informatics support. J Biomed Inform 2009; 42(2): 377–381.

26. Bartlett Ms. Tests Of Significance In Factor Analysis. Br J Stat Psychol 1950; Pages 77-85

27. Kaiser HF. A second generation little jiffy. Psychometrika 1970; **35**, pages401–415

28. Nardi A, Schemper M. Comparing Cox and parametric models in clinical studies. Stat Med 2003; 22(23):3597-610.

29. Davison AC, Hinkley D V. Bootstrap Methods and their Application. 1997. 94(445) Cambridge University Press
